# Optimal information loading into working memory explains dynamic coding in the prefrontal cortex

**DOI:** 10.1073/pnas.2307991120

**Published:** 2023-11-20

**Authors:** Jake P. Stroud, Kei Watanabe, Takafumi Suzuki, Mark G. Stokes, Máté Lengyel

**Affiliations:** ^a^Computational and Biological Learning Lab, Department of Engineering, University of Cambridge, Cambridge CB2 1PZ, United Kingdom; ^b^Graduate School of Frontier Biosciences, Osaka University, Osaka 565-0871, Japan; ^c^Center for Information and Neural Networks, National Institute of Communication and Information Technology, Osaka 565-0871, Japan; ^d^Department of Experimental Psychology, University of Oxford, Oxford OX2 6GG, United Kingdom; ^e^Oxford Centre for Human Brain Activity, Wellcome Centre for Integrative Neuroimaging, Department of Psychiatry, University of Oxford, Oxford OX3 9DU, United Kingdom; ^f^Center for Cognitive Computation, Department of Cognitive Science, Central European University, Budapest H-1051, Hungary

**Keywords:** working memory, dynamic coding, attractor networks, recurrent neural networks, task-optimized networks

## Abstract

Working memory involves the short-term maintenance of information and is critical in many tasks. The neural circuit dynamics underlying working memory remain poorly understood, with different aspects of prefrontal cortical (PFC) responses explained by different putative mechanisms. By mathematical analysis, numerical simulations, and using recordings from monkey PFC, we investigate a critical but hitherto ignored aspect of working memory dynamics: information loading. We find that, contrary to common assumptions, optimal loading of information into working memory involves inputs that are largely orthogonal, rather than similar, to the late delay activities observed during memory maintenance, naturally leading to the widely observed phenomenon of dynamic coding in PFC. Using a theoretically principled metric, we show that PFC exhibits the hallmarks of optimal information loading. We also find that optimal information loading emerges as a general dynamical strategy in task-optimized recurrent neural networks. Our theory unifies previous, seemingly conflicting theories of memory maintenance based on attractor or purely sequential dynamics and reveals a normative principle underlying dynamic coding.

## Significance

The prefrontal cortex (PFC) is known to play a key role in working memory maintenance. However, the PFC has been shown to exhibit unexpectedly rich and complex dynamics during even the simplest working memory tasks—a puzzling phenomenon known as “dynamic coding.” Using mathematical analyses and simulations of task-optimized neural networks, we develop a theory of optimal loading of stimulus information for working memory maintenance and show that dynamic coding in fact naturally arises from this principle. We develop a direct neural measure of optimal information loading, with which we confirm a key prediction of the theory in neural recordings from monkey PFC. Our results show that dynamic coding is a fundamental and functionally useful feature of working memory maintenance.

**Fig. 1.**
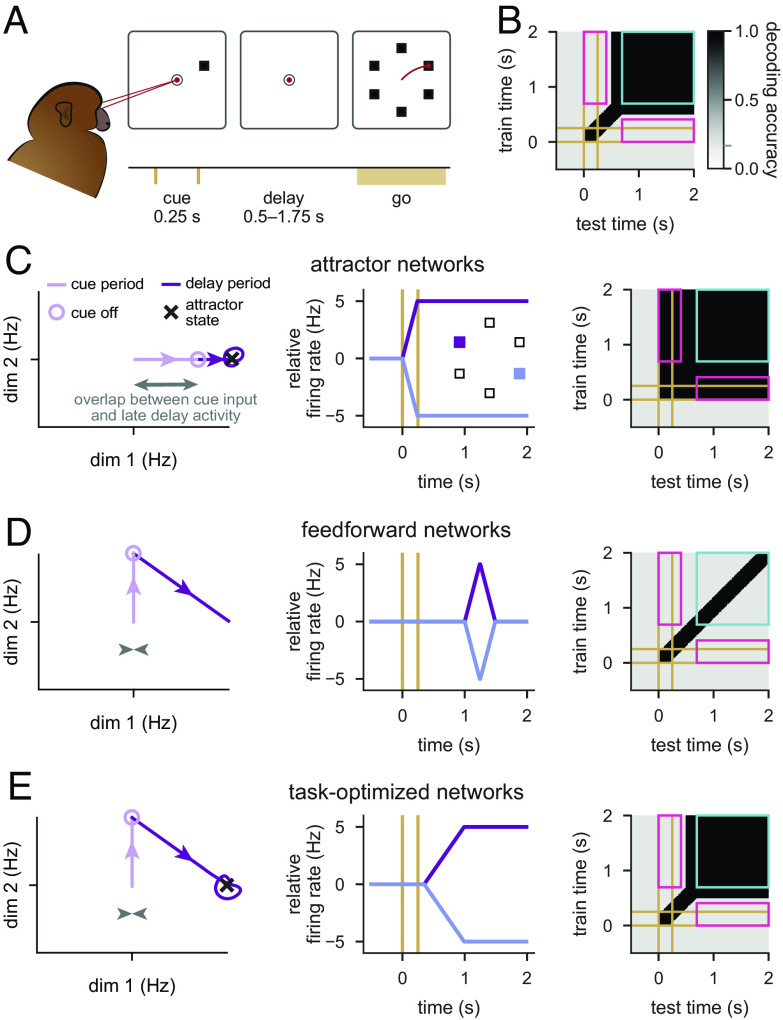
Neural dynamics during working memory: data and model sketch. (*A*) Illustration of the memory-guided saccade task. Time line of task events in a trial (*Bottom*), with the corresponding displays (*Top*). *Top*: The black circle and squares show the fixation ring and the arrangement of visually cued saccade target locations, respectively (not to scale); red dots and the line illustrate gaze positions during fixations and saccade, respectively. *Bottom*: Yellow ticks show the timing of stimulus cue onset and offset; the yellow bar shows the interval within which the go cue can occur. (*B*) Schematic pattern of cross-temporal decoding when applied to neural recordings from the lPFC during working memory tasks (8, 10, 14–16, 24). The grayscale map shows accuracy of decoding cue identity (one out of 6) when the decoder is trained on neural activities recorded at a particular time in the trial (y-axis) and tested at another time (x-axis). Yellow lines indicate cue onset and offset times. Note poor generalization between time points inside the pink rectangle (i.e., dynamic coding), but good generalization between time points inside the cyan square (i.e., stable coding). The gray tick on the color bar indicates chance-level decoding. (*C*) Schematic of neural network dynamics in an attractor network performing the task shown in *A* (see also *SI Appendix*, Fig. S1 *A* and *B*). *Left*: Trajectory in a low-dimensional projection of neural state space in a single cue condition during the cue period (pale purple line, ending in pale purple circle) and delay period (dark purple line). Purple arrowheads indicate the direction of travel along the trajectory, the black cross shows attractor state, and the gray arrow shows overlap between cue input and late delay activity. *Center*: Time course of firing rates (relative to across-condition mean) of a neuron aligned with dim 1 from the *Left* panel for two cue conditions (purple vs. blue, see also *Inset*). Yellow lines indicate cue onset and offset times. *Right*: Cross-temporal decoding of neural activity in the network (cf. *B*; see also *SI Appendix*, Fig. S1 *A* and *B*). (*D* and *E*) Same as *C*, but for a feedforward network that generates sequential activities (20, 26) (*D*; see also *SI Appendix*, Fig. S1*D*) and for a network optimized to perform the task shown in *A* (*E*; see also *SI Appendix*, Fig. S1*E*).

Working memory requires the ability to temporarily hold information in mind, and it is essential to performing cognitively demanding tasks (1, 2). A widely observed neural correlate of the maintenance of information in working memory is selective persistent activity. For example, in the paradigmatic memory-guided saccade task (3–13), subjects must maintain the location of one out of several cues during a delay period after which they must respond with a saccade to the correct location (Fig. 1*A*). Cells in the lateral prefrontal cortex (lPFC) show elevated levels of activity that persist during the delay period and that is selective to the location of the now-absent cue (3–5, 9). However, neurons typically only reach a steady, persistent level of activity late in the delay period of a trial (6, 8, 10, 11, 14–19). In contrast, during the cue and early delay period, neurons in lPFC often exhibit strong transient dynamics during a variety of working memory tasks (3, 8, 10, 11, 14–23).

It remains unknown what mechanism underlies the combination of steady and dynamically changing neural activities in lPFC—especially in light of recent population-level analyses. These analyses, using the technique of “cross-temporal decoding,” place particularly stringent constraints on any candidate neural mechanism of working memory maintenance. Cross-temporal decoding measures how well information about the cue location can be decoded from neural responses when a decoder is trained and tested on any pair of time points during a trial, (refs. (8, 10, 11, 14, 15, 24, 25); Fig. 1*B*). These analyses reveal a consistent but somewhat puzzling set of results. First, when decoder training and testing times are identical, decodability is high (Fig. 1*B*, dark along the diagonal), confirming that information about cue location is indeed present in the population at all times. Decodability is also high when both training and testing occurs during the late delay period, suggesting that even if there are changes in neural responses during this period, the coding of cue location remains stable (Fig. 1*B*, black inside cyan square). However, decoding performance remains low when a decoder is trained during the cue or early delay period and tested during the late delay period, and vice versa (Fig. 1*B*, light gray inside pink rectangles). This demonstrates that the neural code for cue location undergoes substantial change between these these two periods—a phenomenon that has been called “dynamic coding” (8, 10, 14–16, 24).

Classically, the neural mechanism of working memory maintenance is thought to rely on attractor network dynamics. Attractor networks (5, 7, 12, 27–32) and closely related “integrator” networks (33, 34), naturally account for selective persistent activity (Fig. 1*C*, *Left* and *Middle*). However, in these models, neurons show limited transient activity during the delay period, and cross-temporal decoding reveals stable coding throughout the whole trial, lacking the characteristic dynamic coding seen in experimental data (compare Fig. 1 *B* and *C*, *Right*). This behavior emerges across several variants of attractor networks, whether they express a continuum of persistent activity patterns (“ring” or “bump” attractor networks) or a finite number of discrete patterns (*SI Appendix*, Fig. S1 *A* and *B* and S4). Critically, even when external inputs were specifically chosen so that neural activity showed longer transient dynamics (6, 35), these inputs still relied on a large overlap with the desired persistent state (*SI Appendix*, Fig. S1 *C*, *Left*). As a result, these models also exhibited strongly stable stimulus coding over time (*SI Appendix*, Fig. S1 *C*, *Right*), and the transient dynamics were regarded as being purely epiphenomenal (6, 35).

To capture transient dynamics more naturally, a very different class of models has been developed based on mechanisms that generate neural activity sequences. These models typically rely either on effectively feedforward network connectivity (20, 26) or chaotic network dynamics (23, 36–38). The dynamics of such models rapidly transition between orthogonal subspaces over time (Fig. 1*D*, *Left*); thus, cross-temporal decoding is high only between neighbouring time-points (Fig. 1*D*, black along diagonal). Although such models are ideally suited to capturing transient neural responses (Fig. 1*D*, *Center*), they fail to exhibit persistent activities and stable coding during the late delay period (Fig. 1*D*, *Right*; gray inside blue square). Therefore, previous work leaves open two interrelated key questions: How can a neural circuit exhibit early sequential dynamics followed by stable late-delay dynamics and, more importantly, why would it use such a counterintuitive dynamical regime?

In order to study the network mechanisms underlying the combination of dynamic and stable neural activities during working memory, we build on recent advances in using task-optimized neural networks (13, 17, 19, 23, 36, 39–41). Thus, instead of starting from strong prior assumptions about either attractor or sequential dynamics underlying working memory, we train networks for the task of working memory maintenance.

We find that the behaviour of such task-optimized networks unifies attractor and sequential activity models, showing both early dynamic activities and late persistent activities, in line with neural recordings (Fig. 1*E*). To understand the principles and functional significance of this dynamical behavior, we focus on a hitherto ignored aspect of the operation of attractor networks: optimal information loading. That is, we study what transient inputs during the cue period allow a network to most efficiently maintain stimulus information in a stable attractor state subsequently, during the delay period. Through numerical simulations and mathematical analyses, we show that optimal inputs tend to be near-orthogonal to the subsequent attractor state (Fig. 1*E*, *Left*). Critically, this results in an initial period of strong transient dynamics with dynamic coding (Fig. 1*E*, *Right*), which are thus fundamental and functionally useful features of attractor dynamics when used with optimal inputs. Based on our theoretical results, we develop a specific neural measure for assessing whether a network uses optimal information loading. Using this measure, we demonstrate key signatures of optimal information loading in neural recordings from lPFC. Finally, we show that optimal information loading emerges naturally in task-optimized neural networks with a variety of architectures, including linear integrators, as well as nonlinear discrete and ring attractor models.

Our results offer a normative perspective on a core but hitherto ignored component of attractor network dynamics—information loading—and challenge long-held assumptions about pattern completion-like mechanisms in neural circuits.

## Results

### Pattern Completion and Optimal Information Loading in Attractor Networks.

**Fig. 2.**
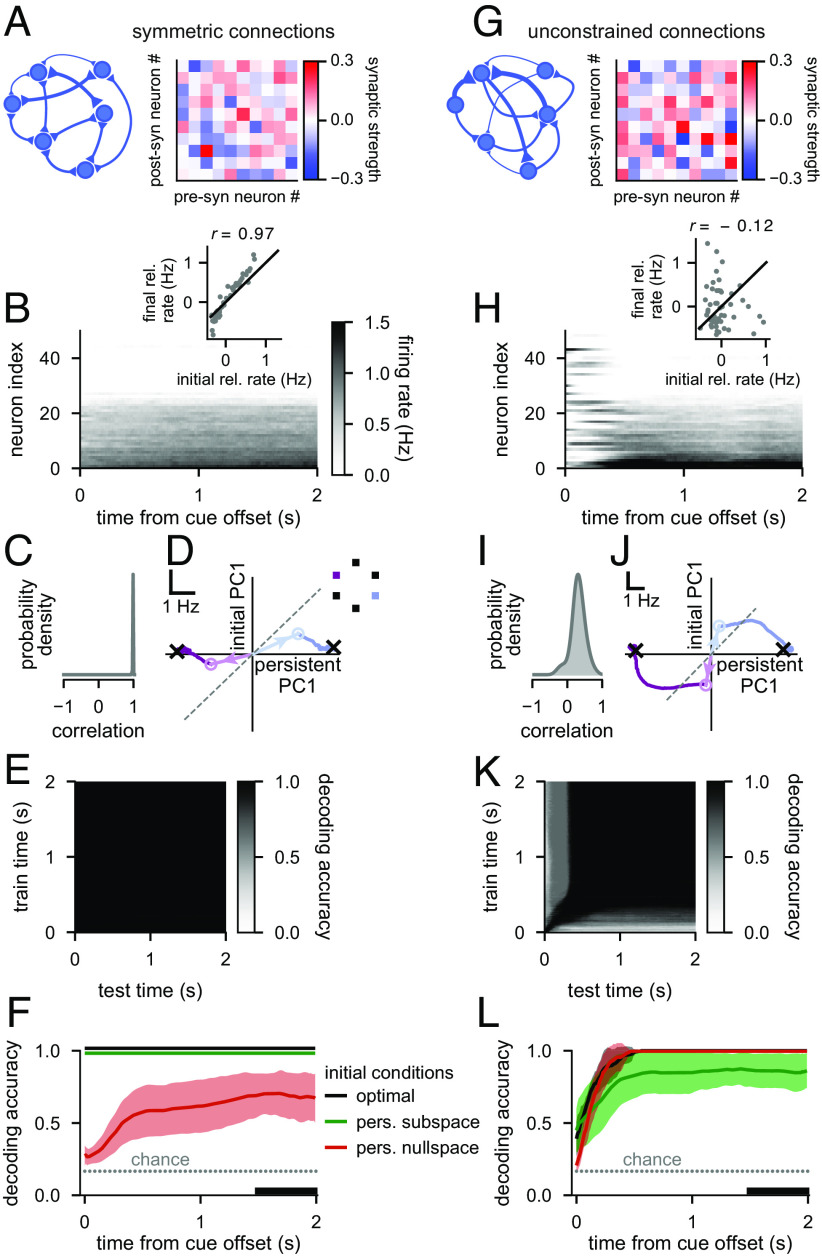
Pattern completion and optimal information loading in attractor networks. (*A*) A network with symmetric connections. *Left*: Network schematic. *Right*: The recurrent weight matrix for 10 of the 50 neurons. (*B*–*F*) Analysis of neural responses in symmetric attractor networks (such as shown in *A*) with optimized initial conditions. (*B*) Firing rates in a representative trial. Neurons are ordered according to their rates at the end of the trial. *Inset* shows initial vs. final firing rates (mean-centered, i.e., relative to the time-dependent but condition-independent mean) across neurons in this trial (gray dots) and their Pearson correlation (r; P<0.001). The black line is the identity line. (*C*) Distribution of Pearson correlations between initial and final mean-centered neural firing rates across all six cue conditions and 10 networks. (*D*) Subthreshold activity for two cue conditions in an example network. The horizontal axis (persistent PC1) shows network activity projected onto the 1st principal component (PC1) of activities at the end of the delay period (across the two conditions shown in the *Inset*), and the vertical axis (initial PC1) shows projection to PC1 of initial activities orthogonalized to persistent PC1. Pale open circles with arrows pointing to them from the origin show the optimized initial conditions (the starting point for network dynamics), dark traces show activity trajectories generated by network dynamics, black crosses show stable fixed points, and the dashed gray line is the identity line. (*E*) Cross-temporal decoding of neural firing rate activity (cf. Fig. 1*B*). (*F*) Performance of a delay-trained decoder (black bar indicates decoding training time period) on neural firing rate activity over time starting from optimized initial conditions with full optimization (black), or for initial conditions optimized but restricted to the 5-dimensional subspace spanning the six cue-specific attractors (persistent subspace, green), or the subspace orthogonal to that (persistent nullspace, red). Note that decoding is always performed in the full state space. Solid lines and shading indicate mean ± 1 s.d. across all six cue conditions and 10 networks. The gray dotted line shows chance level decoding. Green and black lines are slightly offset vertically to aid visualization. (*G*–*L*) Same as *A*–*F*, for attractor networks with unconstrained connections. The Pearson correlation in *H* (*Inset*) is not significant (P>0.4).

Traditional approaches to studying working memory with attractor networks used models in which the connectivity between neurons was constrained to be effectively symmetric (5, 7, 27, 29, 31, 33, 34, 42–45). This was motivated by their mathematical tractability and consequent guarantees on their dynamical and computational properties, such as convergence to attractor states, noise tolerance, and memory capacity (27, 32, 33, 42, 46). Thus, we first replicated results with symmetric networks that were optimized to perform the working memory task shown in Fig. 1*A*. We defined optimal information loading to be achieved by a set of inputs when they maximize the performance of a network in terms of how well the cue can be decoded from its neural activities at the end of the delay period. For simplicity, we only modelled the intrinsic dynamics of the network during the delay period, and the effect of the cue was captured by cue-specific initial neural activities, i.e., neural activities at the beginning of the delay period (see refs. (34, 42, 43); Fig. 2*B*). To achieve optimal information loading, we optimized these initial activities for cue decodability at the end of the delay period (under constraints on their magnitude, below we also show results for biologically more relevant constraints, see also *SI Appendix*, *Materials and Methods*, S2.3.1).

Optimal initial activities gave rise to classical pattern completion dynamics in symmetric networks. First, initial activities were noisy versions of (and in fact highly similar to) the desired persistent patterns (Fig. 2*B*, *Inset*, and *C*). Second, the ensuing dynamics were driven directly into the corresponding persistent state, resulting in only small and gradual changes in activities over the delay period (Fig. 2*B*). Further analysis of these dynamics showed that the optimal initial activities aligned well with directions in neural state space that best distinguished between the desired persistent activities (Fig. 2*D*, “persistent PC1” component of open circles with pale arrows pointing to them; *SI Appendix*, Fig. S2*B*), with only a comparably small component in orthogonal directions specific to these initial activities (Fig. 2*D*, “initial PC1”) which subsequently changed little over time (Fig. 2*D*, dark trajectories). As a result, cross-temporal decoding performance was high for all pairs of times (Fig. 2*E*), and—as a special case—a decoder trained to decode neural activity during the late delay period (i.e., during the steady state of the network), generalized well to all times and was able to decode the cue identity from neural activities with high accuracy throughout the delay period (Fig. 2*F*, black line).

The similarity between initial and persistent activities was critical for these networks. When constrained to use initial activities that were orthogonal in neural state space to persistent activities (i.e., lying in the “persistent nullspace”), these networks performed substantially more poorly (Fig. 2*F*, red line) and activity often did not settle into the correct attractor state (*SI Appendix*, Fig. S2*D*). In contrast, explicitly enforcing these networks to use initial activities that were similar to persistent activities (i.e., lying in the “persistent subspace”) did not compromise their performance (Fig. 2*F*, green line and *SI Appendix*, Fig. S2*C*). Thus, when connectivities were constrained to be symmetric, our approach using explicitly optimized inputs and connectivities recapitulated earlier results obtained with classical attractor networks using hand-crafted inputs and connectivities (5, 7, 12, 27, 29, 32, 42).

Next, we studied attractor networks optimized without a symmetry constraint, as real neural connectivity in the PFC is unlikely to be symmetric, e.g., due to separate classes of excitatory and inhibitory neurons (31, 47, 48). Such unconstrained attractor networks exhibited dynamics distinctly unlike simple pattern completion (Fig. 2*G*–*L*). First, initial activities resembled persistent activities much less than in symmetric networks (Fig. 2*I*), such that their correlation could even be negative (Fig. 2*H*, *Inset*). Second, neural activities often underwent substantial and non-monotonic changes before ultimately settling into an attractor state (Fig. 2*H*). This was also reflected in optimal initial activities (Fig. 2*J*, open circles with pale arrows pointing to them) being near-orthogonal to persistent activities (Fig. 2*J*, black crosses and *SI Appendix*, Fig. S2*F*), with this orthogonality decaying over the delay period (Fig. 2*J*, dark trajectories). Such dynamics are consistent with PFC recordings from primates performing a variety of working memory tasks (8, 17, 21–23, 31, 49). Decoding analyses revealed further similarities with experimental data: a decoder trained on neural activity from the late delay period generalized poorly to early times (Fig. 2*K*, and *L*, black line) and vice versa (Fig. 2*K*), thus exhibiting a fundamental signature of “dynamic coding” (8, 10, 14–16) (cf. Fig. 1*B*). Importantly, we found that the orthogonality of initial conditions in these networks was instrumental for high performance: in a double dissociation from symmetrically constrained networks, restricting initial conditions to be in the persistent subspace (Fig. 2*L*, green line and *SI Appendix*, Fig. S2*G*), but not in the persistent nullspace (Fig. 2*L*, red line and *SI Appendix*, Fig. S2*H*), diminished decodability at the end of the delay period (cf. Fig. 2*F*).

The above results were obtained with networks storing a small number of discrete attractors, corresponding to the six cue conditions. Previous work found that several aspects of working memory dynamics in lPFC are better captured by networks in which instead a large number (or even a continuum) of attractor states form a ring in neural state space (5, 7, 44, 45). Thus, we repeated our analyses on optimized networks while explicitly encouraging such a ring attractor to form (*SI Appendix*, *Materials and Methods* S2.3.4). The pattern of results obtained with these ring attractor networks was highly similar to what we found in discrete attractor networks (*SI Appendix*, Fig. S3).

### Dynamical Analysis of Optimal Information Loading.

To understand why optimal information loading in classical symmetrically constrained versus unconstrained attractor networks is so different and, in particular, why inputs orthogonal to attractor states are optimal for unconstrained networks, we reduced these networks to a canonical minimal model class consisting of only two neurons (34, 47, 48). For analytical tractability, we considered networks with linear dynamics (i.e., in which neurons had linear activation functions). Critically, with the appropriate set of synaptic connections, even linear networks can exhibit persistent activity (6, 33–35, 46, 50)—the key feature of working memory maintenance in attractor networks.

**Fig. 3.**
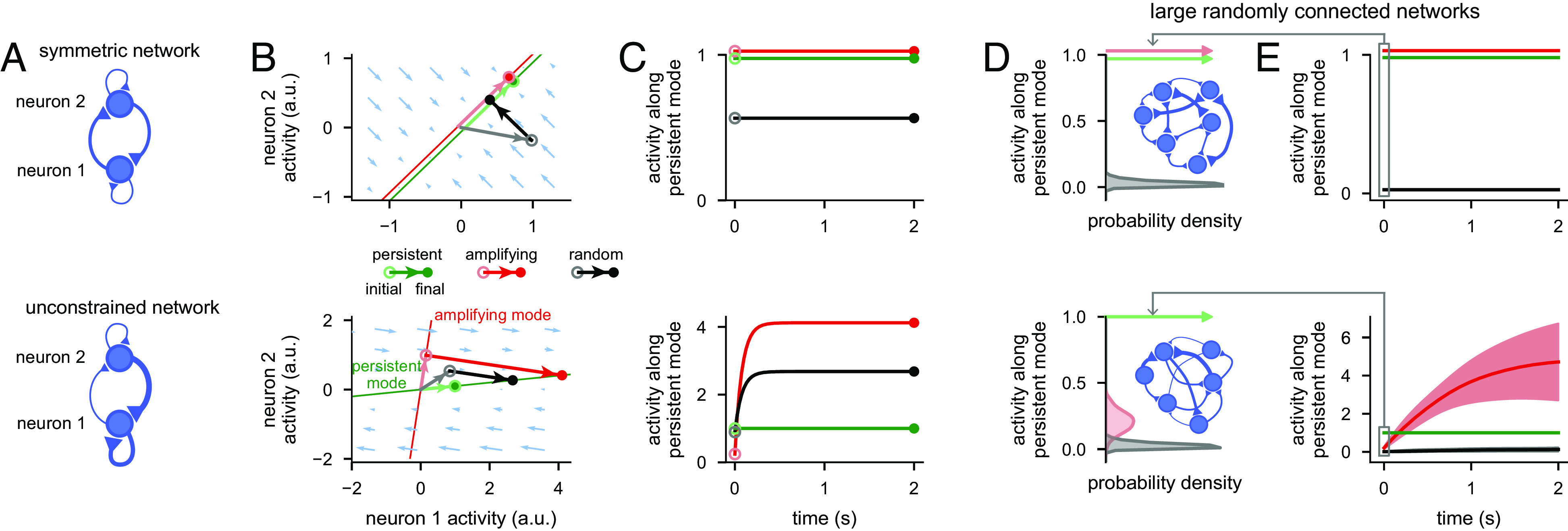
Dynamical analysis of optimal information loading. (*A*) Architecture of a symmetric (*Top*) and an unconstrained network (*Bottom*). (*B*) Neural state space of the symmetric (*Top*) and unconstrained network (*Bottom*). Pale blue arrows show flow field dynamics (direction and magnitude of movement in the state space as a function of the momentary state). Thin green and red lines indicate the persistent and most amplifying modes, respectively (lines are offset slightly in the *Top* panel to aid visualisation). Pale green, red, and gray arrows with open circles at the end indicate persistent, most amplifying, and random initial conditions, respectively. Dark green, red, and black arrows show neural activity trajectories starting from the corresponding initial condition. (Green arrows and the red arrow in the *Top* panel cannot be seen, as no movement in state space happens from those initial conditions.) Filled colored circles indicate final (persistent) neural activity. (*C*) Time course of network activity along the persistent mode (i.e., projection onto the green line in *B*) when started from the persistent (green), most amplifying (red), or random initial conditions (black) for the symmetric (*Top*) and the unconstrained model (*Bottom*). (*D*) Distributions of absolute overlap with the persistent mode for persistent (pale green), most amplifying (pale red), or random initial conditions (gray) across 100 randomly connected 1,000-neuron symmetric (*Top*) or unconstrained networks (*Bottom*). The persistent (and for the symmetric models, also the equivalent most amplifying) initial conditions produce delta functions at 1 (arrows). Insets show illustration of large networks of neurons with either symmetric (*Top*) or unconstrained (*Bottom*) connections. (*E*) Time course of absolute overlap with the persistent mode when starting network dynamics from persistent (green), most amplifying (red), or random initial conditions (black) for the symmetric (*Top*) and the unconstrained network (*Bottom*). Lines and shaded areas show mean ± 1 s.d. over the 100 randomly sampled 1,000-neuron networks from *D*.

**Fig. 4.**
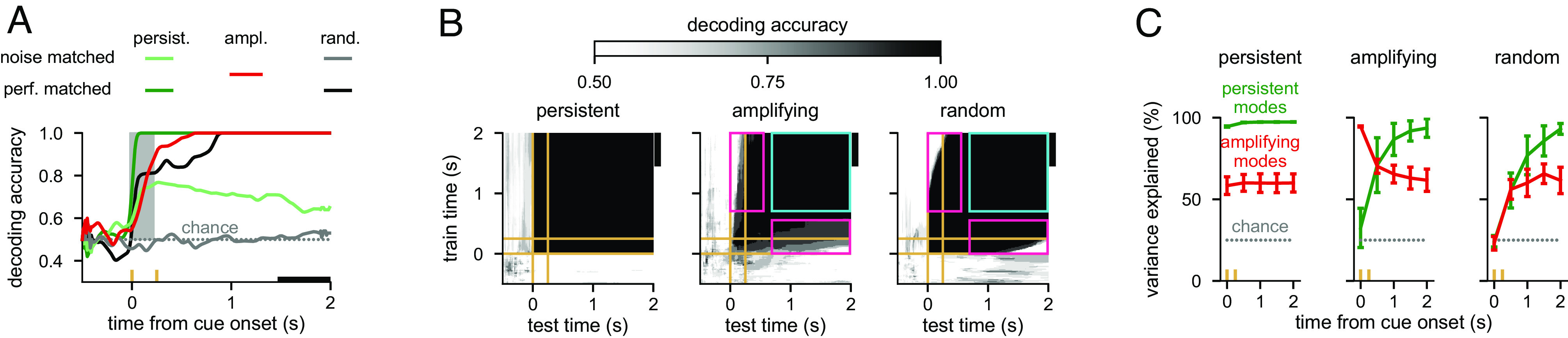
Neural signatures of optimal information loading. (*A*) Performance of a delay-trained decoder (black bar indicates decoder training time period) on neural activity over time. Two cue conditions were used with inputs that were identical but had opposite signs. Lines show mean across 10 randomly connected 100-neuron linear unconstrained networks. Yellow ticks on the horizontal axis indicate cue onset and offset times, and the gray shading indicates the cue period. We show results for inputs aligned with the persistent mode (dark and pale green), the most amplifying mode (red), or a random direction (black and gray). Light colors (pale green and gray, “noise-matched”) correspond to networks with the same level of noise as in the reference network (red), while dark colors (dark green and black, “performance-matched”) correspond to networks with the same level of asymptotic decoding performance as that in the reference network (red). The gray dotted line shows chance level decoding. (*B*) Cross-temporal decoding of neural activity for the three different information loading strategies (persistent, most amplifying, and random respectively in *Left*, *Center*, and *Right* panels) for a representative network for the performance-matched condition from *A*. Yellow lines indicate cue onset and offset times. Pink rectangles indicate poor generalization between time points (i.e., dynamic coding), and cyan squares indicate examples of good generalization between time points (i.e., stable coding). The black vertical bars on the Right of each plot indicate the delay-trained decoder training time period from *A*. (*C*) Percent variance of responses explained by the subspace spanned by either the 25% most persistent (green) or 25% most amplifying (red) modes as a function of time in the same networks analyzed in *A*. Lines and error bars show mean ± 1 s.d. across networks. We show results for inputs aligned with the persistent mode (*Left*), most amplifying mode (*Center*), or a random direction (*Right*). The gray dotted line shows chance level overlap with a randomly chosen subspace occupying 25% of the full space.

For our analyses, we again distinguished between models with symmetric connectivity (Fig. 3*A*, *Top*) (33, 34, 48), and models without this constraint (Fig. 3*A*, *Bottom*) (6, 35). In either case, the specific connection strengths were chosen to create illustrative examples providing intuitions that—as we show below—also generalize to large networks with randomly sampled connection strengths (Figs. 3 *D* and *E* and 4). The dynamics of these networks are fully described in a two-dimensional neural state space spanned by the activities of the two neurons (Fig. 3*B*) and define a flow-field in this space determining how neural activities change over time (Fig. 3*B*; blue arrows). An important subspace of the full neural state space of these networks is the “persistent subspace” corresponding to persistent patterns of activities. In our two-neuron linear networks, the persistent subspace simply corresponds to a line onto which the neural activities ultimately converge over time (Fig. 3*B*; green lines showing the persistent mode). Therefore, the persistent mode allows these networks to distinguish between two stimuli depending on which side of the origin the state of the network is. The larger the magnitude of its activity along this persistent mode at the end of the delay period, the more robustly the identity of the stimulus can be decoded (e.g., in the presence of noise, as we show below).

To understand the mechanisms of information loading, we considered three distinct stimulus input directions (with unit magnitude). We then analysed the time course of the neural activities projected onto the persistent mode (6, 29, 35) after being initialised in each of these directions. First, we considered inputs aligned with the persistent mode, the input direction studied in classical attractor networks (6, 33–35, 48) (Fig. 3*B*; pale green arrows and open circles). Second, we considered the “most amplifying mode,” which is defined as the stimulus direction that generates the most divergent and thus best discriminable activity over time (51–53) (*SI Appendix*, *Materials and Methods*, S2.7.1; Fig. 3*B*, red lines, and pale red arrows and open circles). Third, we considered a random input direction (Fig. 3*B*; gray lines/circles).

We were able to show mathematically that optimal information loading, in the sense of maximizing overlap with the persistent mode at sufficiently long delays, is always achieved with inputs aligned with the most amplifying mode (*SI Appendix*, S5). Equivalently, the most amplifying mode is the input direction that requires the smallest magnitude initial condition to achieve a desired level of persistent activity (i.e., a desired level of performance). More generally, we could also show both mathematically and in simulations (*SI Appendix*, Fig. S4) that the most amplifying mode is near optimal in achieving a desired level of performance while minimizing total neural activity over time (i.e., the total energy used by the network) for sufficiently long delay lengths.

In symmetric networks, the most amplifying mode is aligned with the most persistent mode (Fig. 3*B*, *Top* and *SI Appendix*, S5.1), and thus does not generate activity transients (Fig. 3*C*, *Top*)—accounting for the simple pattern completion dynamics seen in classical attractor networks with symmetric connectivity (5, 7, 27, 29, 31, 33, 34, 42, 43) (Fig. 2*A*–*F*). However, in unconstrained networks, the most amplifying mode is typically different from the most persistent mode (Fig. 3*B*, *Bottom*). Intuitively, this is because effective feedforward connections exist in unconstrained networks (20, 26, 47, 54). For example, neurons 1 and 2 in the example network shown in Fig. 3*A* (*Bottom*) respectively align strongly with the persistent and amplifying modes (Fig. 3*B*, *Bottom*). Thus, feeding neuron 1 indirectly through the feed-forward connection from neuron 2 can increase its activity more than just feeding it directly. This means that activity evolving from the most amplifying mode exhibits a distinct transient behaviour: its overlap with the most persistent mode is initially low and then increases over time (Fig. 3*C*, *Bottom*, red line), accounting for the richer transients seen in unconstrained attractor networks (Fig. 2*G*–*L*). Therefore, there is a form of “speed–accuracy” trade-off between whether inputs should use the most amplifying or persistent mode: if information is required immediately following stimulus offset, such as in a perceptual decision-making task (13, 40), inputs need to use the persistent mode–in line with recent experimental evidence (55). However, if there is a time delay until the information is needed, as is the case in all working memory tasks (2), then the most amplifying mode becomes the optimal input direction. Indeed, an analogous trade-off was already apparent between the persistent sub- vs. nullspace inputs in the nonlinear attractor networks we analysed earlier (Fig. 2*L*, red vs. green).

The insights obtained in the simple two-neuron network also generalized to large randomly connected linear integrator networks, with more than two neurons (Fig. 3 *D* and *E* and *SI Appendix*, *Materials and Methods*, S2.4.1). Moreover, as network size grows, in unconstrained (but not in symmetric) networks, the most amplifying direction becomes increasingly orthogonal to the most persistent mode (56), further accentuating the advantage of amplifying over persistent mode inputs (56) (Fig. 3 *D* and *E* and *SI Appendix*, Fig. S4 *A* and *B*; red vs. green). This is because in large unconstrained networks, there are many effectively feedforward motifs embedded in the full recurrent connectivity of the circuit, which can all contribute to transient amplification (20). Random initial conditions become fully orthogonal in both networks and result in poor overlap with the persistent mode (Fig. 3 *D* and *E* and *SI Appendix*, Fig. S5 *A* and *B*; black). Numerical simulations confirmed that these results also generalized to networks with noisy dynamics (*SI Appendix*, Fig. S5*C*). Moreover, explicitly optimizing the initial condition of such a network so as to maximize the persistent activity it generated at the end of a delay period also made this initial condition overlap strongly with the network’s most amplifying mode (*SI Appendix*, Fig. S5*D*).

As our mathematical analyses only applied to linear dynamics, we used numerical simulations to study how they generalized to nonlinear dynamics. We found that the same principles applied to the dynamics of a canonical 2-dimensional nonlinear attractor system (analogous to the networks in Fig. 3*A* –*C*), when the persistent and most amplifying directions were defined locally around its ground state (*SI Appendix*, *Materials and Methods*, S2.6, S6, and Fig. S6). Importantly, we also found that large optimized nonlinear neural networks (with discrete or ring attractors) also showed a similar pattern of results (*SI Appendix*, S7 and Figs. S3*E* and S7 *A*–*C*).

### Neural Signatures of Optimal Information Loading.

Our dynamical analysis suggested that there should be clearly identifiable neural signatures of a network performing optimal information loading. To demonstrate this, and to allow a more direct comparison with data, we used the same large, randomly connected, unconstrained networks that we analysed earlier (Fig. 3 *D* and *E*, *Bottom*), with noisy dynamics (as in *SI Appendix*, Fig. S5 *C* and *D*) and the cue period modelled using temporally extended constant inputs—mimicking typical experiments (3–5, 10) (Fig. 4). We studied the three different information loading strategies that we identified earlier: Inputs aligned with either the persistent mode, the most amplifying mode, or a cue-specific random direction.

We began by conducting a decoding analysis using templates of late delay activity, as is often done for prefrontal cortical recordings (6, 8, 10, 14, 15, 24) (and also in Fig. 2 *F* and *L*). We first verified that for a fixed level of neuronal noise, the most amplifying inputs were indeed optimal for achieving high decodability at the end of the delay period (Fig. 4*A*, compare the red line to pale green and gray lines). We were also able to show mathematically that, in line with our original definition of optimal information loading, the most amplifying inputs in noisy linear networks are optimal for maximizing average decodability during the delay period (*SI Appendix*, S5.7). In contrast to most amplifying inputs, persistent and random inputs performed considerably more poorly (Fig. 4*A*, pale green and gray lines).

The level of noise in the networks we have studied so far was not constrained by data, which typically shows high decodability at the end of the trial (6, 8, 10, 14, 15, 24). This is important because the suboptimal input conditions (Fig. 4*A*, pale green and gray lines) could achieve high decoding performance by appropriately reducing the noise level in our simulations (Fig. 4*A*, asymptotic values of dark green and black lines). Thus, asymptotic decoding performance alone cannot be used to identify the information loading strategy employed by a network. To address this, in subsequent analyses, we used networks in which the level of late-delay performance was matched between the three information loading strategies by appropriately reducing the level of noise when using persistent or random inputs. Nevertheless, a critical difference emerged between the different information loading strategies even in these “performance-matched” networks. For both random and most amplifying input directions, the delay-trained decoder only performed well when tested late in the delay period (Fig. 4*A*, black and red lines), whereas for inputs aligned with the persistent direction, this decoder performed near ceiling at all times after cue onset (Fig. 4*A*, dark green line).

Next, in order to more fully characterise the differences between persistent versus random or most amplifying inputs, and for a comprehensive comparison with experimental data (8, 10, 14, 15, 24), we also employed full cross-temporal decoding (Fig. 4*B*). This analysis showed that all information loading strategies led to dynamics in which stimulus information was present at all times after cue onset (Fig. 4*B*, diagonals are all black). Moreover, for the persistent mode inputs, stimulus information was maintained using a “stable code” (10, 11, 14, 16) (Fig. 4*B*, *Left*, all off-diagonals are black)—similar to previous integrator models of working memory (33, 34) (*SI Appendix*, Fig. S1*C*).

In contrast, random and most amplifying mode inputs led to poor cross-temporal decodability between early and late time points after cue onset (Fig. 4*B*, *Center* and *Right*, off-diagonals indicated by pink rectangles are white/gray). This gave rise to the phenomenon of “dynamic coding” (8, 10, 11, 14–16), and suggested sequential activities during the early-to-late delay transition (20, 26, 36). These activities then stabilised during the late delay period as the network dynamics converged to a persistent pattern of activity (Fig. 4*B*, *Center*, and *Right*, off-diagonals inside cyan squares are black). In sum, these decoding analyses were able to clearly distinguish between persistent mode and random or amplifying inputs, but not between the latter two.

To clearly distinguish between networks using most amplifying inputs or merely a random input direction, we constructed a targeted measure for identifying networks using most amplifying inputs. To achieve this, we exploited the fact that in large networks, random inputs typically have negligible overlap with any other direction in neural state space, including the most amplifying mode. Thus, we directly measured the time courses of the overlap of neural activities with the top 25% most amplifying modes. We quantified this overlap as the fraction of across-condition variance of neural activities that these modes collectively explained (Fig. 4*C*, red lines and *SI Appendix*, *Materials and Methods*, S2.7.3). For a comparison, we also measured the overlap of neural activities with the top 25% most persistent modes (Fig. 4*C*, green lines).

Persistent mode inputs led to constant high and moderate overlaps with the persistent and most amplifying modes, respectively (Fig. 4*C*, *Left*). Random inputs started with chance overlap for both modes, which then increased to the same levels that resulted from persistent mode inputs (Fig. 4*C*, *Right*). In contrast, most amplifying inputs were uniquely characterised by a cross-over between the time courses of the two overlap measures. Initially, neural activities overlapped strongly with the most amplifying mode, but showed only chance overlap with the persistent mode (Fig. 4*C*, *Middle*). Over time, these overlap measures changed in opposite directions, such that by the end of the delay period overlap was high with the persistent mode and lower with the most amplifying mode (Fig. 4*C*, *Middle*). Therefore, the cross-over of these overlap measures can be used as a signature of optimal information loading utilizing inputs aligned with the most amplifying modes.

To further illustrate how our overlap measures can distinguish between optimal and random input directions, we modified an earlier integrator model of working memory (6) (*SI Appendix*, Figs. S1*C* and S8 *A* and *D*) so that inputs lay in a purely randomly oriented subspace. This resulted in cross-temporal decoding matrices that looked similar to that achieved by the most amplifying mode (*SI Appendix*, Fig. S8*B*), but the overlap measures that we developed here clearly revealed the lack of optimal information loading, even in this modified model (*SI Appendix*, Fig. S8*E*). In addition, we confirmed in numerical simulations that the same signature of optimal information loading remains detectable even under the practical constraints of experimental data analysis: when the underlying network dynamics is nonlinear and only accessible indirectly by fitting linear dynamical models to the neural responses they generate (*SI Appendix*, Fig. S7*D*, *Materials and Methods*, S2.4.3, and S7.4).

### Signatures of Optimal Information Loading in Monkey lPFC.

**Fig. 5.**
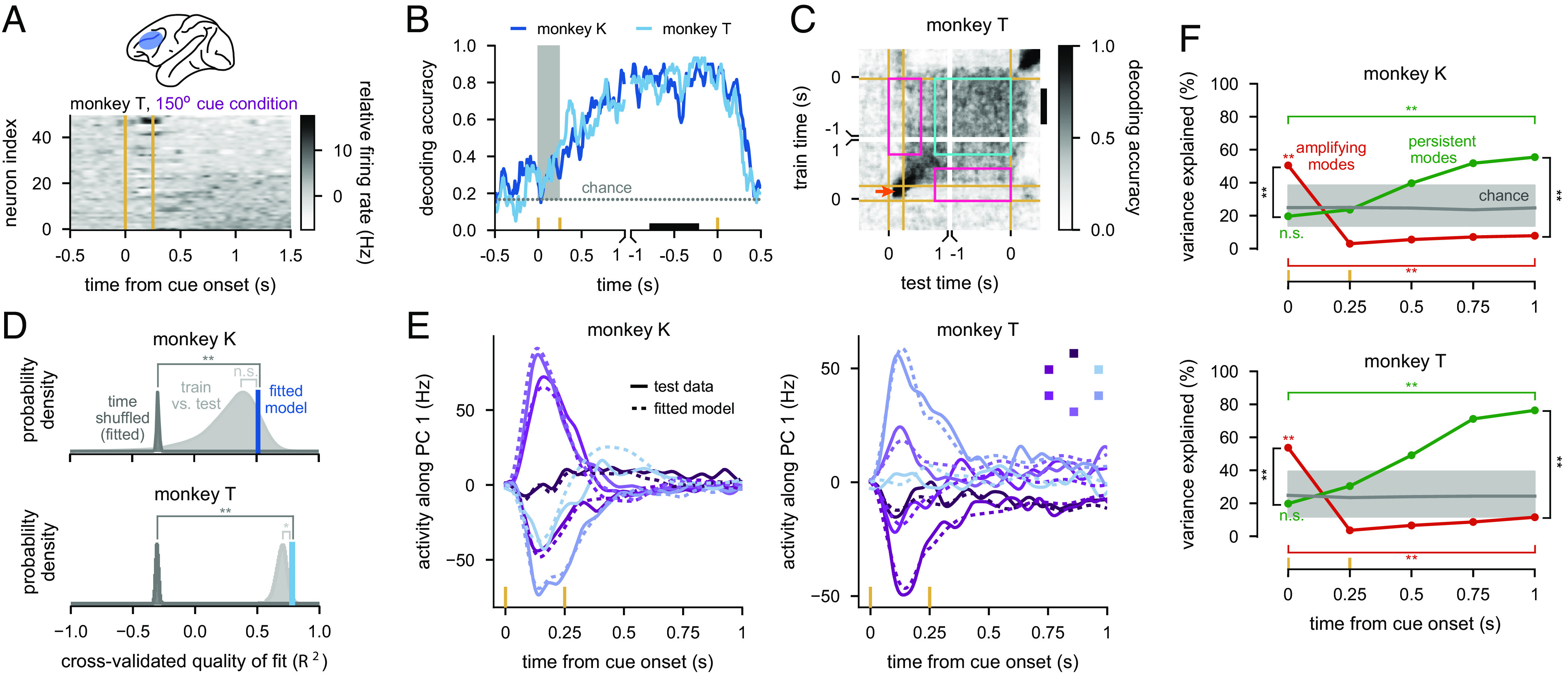
Signatures of optimal information loading in monkey lPFC. (*A*) *Top*: lPFC recording location. *Bottom*: Neural firing rates (relative to the time-dependent but condition-independent mean) for one stimulus cue condition for 50 example neurons. See Fig. 1*A* for experimental paradigm. Neurons are ordered according to their firing rate at the end of the period shown. Vertical yellow lines indicate stimulus cue onset and offset. (*B*) Performance of a delay-trained decoder (the black bar indicates decoder training time period) on neural activity over time. Yellow ticks on the horizontal axis indicate stimulus cue onset, offset, and go cue times, and the gray shading indicates the stimulus cue period. Data are aligned to either stimulus cue onset (first 1.5 s) or to the go cue (final 1.5 s). The gray dotted line shows chance level decoding. (*C*) Cross-temporal decoding of neural activity for monkey T (see *SI Appendix*, Fig. S10*A* for Monkey K). Yellow lines indicate stimulus cue onset, offset, and go cue times. Pink rectangles indicate poor generalization between time points (i.e., dynamic coding) and the cyan square indicates examples of good generalization between time points (i.e., stable coding). The orange arrow indicates good same-time decoding during the cue period. The black vertical bar on the right indicates the delay-trained decoder training time period from *B*. (*D*) Cross-validated quality of fits when fitting 20-dimensional linear neural networks to neural activity (blue) and time-shuffled controls (dark gray) for monkey K (*Top*) and monkey T (*Bottom*). We also show quality of fits of the data against itself (“train vs. test”; light gray). (*E*) Neural activity for each of the 6 cue conditions projected onto the top PC (solid lines) for monkey K (*Left*) and monkey T (*Right*). Solid lines show held-out test data, and dashed lines show predictions of fitted model dynamics. The inset for monkey T shows which color corresponds to each cue condition. (*F*) Percent variance of responses explained by the subspace spanned by either the 25% most persistent (green) or 25% most amplifying (red) modes as a function of time for the 20-dimensional linear neural networks fitted to data from monkey K (*Top*) and monkey T (*Bottom*). Gray lines show chance level overlap defined as the expected overlap with a randomly chosen subspace occupying 25% of the full space (median and 95% C.I. across 200 random subspaces). Comparisons shown in *D* and *F* use two-sided permutation tests (*P<0.05; **P<0.01; n.s., not significant).

To study whether the PFC shows the dynamical signatures of optimal information loading that our theoretical analyses identified, we analysed a data set (49) of multichannel recordings of the lateral prefrontal cortex (lPFC) in two monkeys during a variable-delay memory-guided saccade task (Fig. 1*A*). These recordings yielded 438 and 625 neurons (for monkeys K and T, respectively; *SI Appendix*, Fig. S9 and *Materials and Methods*, S2.1). We analysed the population dynamics of all recorded neurons in each monkey and applied the same metrics to this dataset that we applied to our models. Population dynamics appeared to show rich transient dynamics during the cue and early delay period, followed by relatively stable dynamics during the late delay period (Fig. 5*A*). This was reminiscent of the dynamics we found in unconstrained attractor networks following optimal information loading (Fig. 2*H*).

To further quantify this behaviour, we conducted decoding analyses. First, we found that a delay-trained decoder did not generalize to times outside of the delay period (Fig. 5*B*). In particular, performance was near-chance level during the cue period and increased over the first 1 s of the delay period—in line with previous studies (6, 10, 14–16, 24). This was distinct from the pattern completion dynamics seen in classical attractor network models of working memory (Figs. 2 *F* and *L* green and 4*A* green), but similar to that expected from random or optimal inputs in unconstrained networks (Figs. 2*L*, black and red and 4*A*, *Bottom*, black and red).

Full cross-temporal decoding reinforced these results: decoders trained during the delay period did not generalize to the cue or go periods and vice versa (Fig. 5*C* and *SI Appendix*, Fig. S10*A*, pink rectangles). Thus, neural activity exhibited dynamic coding (14, 15) rather than the stable coding characteristic of simple pattern completion (Figs. 1*C*, *Right*, and 4*B*, *Left*, and *SI Appendix*, Fig. S1 *A*–*C*, *Right*). Importantly, same-time decoding performance was close to 1 throughout the cue and delay periods (Fig. 5*C* and *SI Appendix*, Fig. S10*A*, orange arrow). This confirmed that the poor cross-temporal generalization between early and late periods of a trial was not because the cue information had not yet reached PFC or was maintained by activity-silent mechanisms (11, 41, 45). At the same time, also in line with previous studies (8, 10, 14–16), we found relatively stable coding during the late delay period (Fig. 5*C* and *SI Appendix*, Fig. S10*A*, cyan square). This ruled out purely sequential activity-based dynamics (20, 26, 37, 38) (Fig. 1*D* and *SI Appendix*, Fig. S1*D*).

Quantifying the relative alignment of the subspaces occupied by neural dynamics across time using PCA (6, 57) confirmed the orthogonality of neural activities between different task periods (*SI Appendix*, Fig. S10 *B*–*C*). Further analyses showed that this orthogonality was not simply due to distinct subpopulations of neurons being active in different task periods (due to either feedforward connections between these populations, or single-neuron adaptation mechanisms) but was instead largely due to changes in population-wide activities patterns (10) (*SI Appendix*, Fig. S10 *D* and *E*).

These results, in line with previous findings (8, 10, 15, 16), clearly indicated that activities during the cue period were near-orthogonal from those during the delay period. However, these analyses alone were unable to distinguish between two fundamentally different information loading strategies PFC could employ: random input directions, or optimal input directions. Thus, in order to clearly identify the information loading strategy underlying the combination of dynamic and stable coding that we found, we applied our overlap measure (Fig. 4*C*) to these PFC recordings. For this, we first fitted a 20-dimensional linear dynamical system model to the cue and early delay periods of our recordings (0–1 s after cue onset, *SI Appendix*, *Materials and Methods*, S2.4.3). We confirmed that linear dynamics provided a reasonably accurate cross-validated fit to the data compared to a time-shuffled control (which destroyed the lawful dynamics of the data; Fig. 5*D*, dark gray, see also *SI Appendix*, *Materials and Methods*, S2.4.3), and model-free train vs. test performance (which indicated that cross-validated errors were mostly due to sampling noise differences between the train and test data; Fig. 5*D*, light gray), and they recapitulated the most important aspects of the trial-average dynamics in each condition (Fig. 5*E*).

We then performed the same overlap analysis on the fitted linear dynamics of the data that we used on our simulated networks with linear dynamics (Fig. 4*C* and *SI Appendix*, *Materials and Methods*, S2.7.3). As expected from our decoding analyses (Fig. 5 *B* and *C*), the overlap of neural activities with the most persistent modes was at chance initially and gradually increased (Fig. 5*F*, green and *SI Appendix*, Fig. S10*I*). Critically, however, the overlap of neural activities with the most amplifying modes was high initially and decreased with time (Fig. 5*F*, red and *SI Appendix*, Fig. S10*I*). Consistent with these results, we found that at early times, stimulus information was just as decodable within the amplifying subspace as in the full space and was more poorly decodable in the persistent subspace (*SI Appendix*, Fig. S10*H*, t=0). Later in the delay period, stimulus information was significantly better decodable in the persistent subspace than in the amplifying subspace (*SI Appendix*, Fig. S10*H*, t>0).

We also noted that the overlap with the most amplifying directions became significantly lower than chance over time. This suggests that PFC circuits may be more mathematically “non-normal” (20, 26, 54) than the networks with randomly chosen weights that we used in Fig. 4. For example, *SI Appendix*, Fig. S8*F* shows this phenomenon in a highly non-normal (purely feedforward) network using optimal information loading (*Discussion*). Indeed, when explicitly measuring the level of non-normality in our models fitted to our neural recordings, we found that they had a mean Henrici’s index (55) of 0.83 (which was greater than the 0.69 mean Henrici’s index in the randomly initialised networks; see *SI Appendix*, *Materials and Methods*, S2.7.9). This indicates that lPFC dynamics are strongly non-normal.

As a control, we repeated the same analyses on time-shuffled data, or on data taken from the late delay period (when the network should already be near an attractor state). Neither control analyses resulted in the same cross-over pattern that we found in our main analysis. In particular, the overlap with the most amplifying modes remained at (or below) chance at all times (*SI Appendix*, Fig. S10 *F*, *G*, and *I*).

Therefore, these analyses provide strong experimental evidence that PFC circuit dynamics utilize optimal information loading with inputs aligning with the most amplifying modes (compare to Fig. 4*C*, *Middle* and *SI Appendix*, Fig. S10*I*, third vs. fourth row) rather than simply using random input directions (compare to Fig. 4*C*, *Right* and *SI Appendix*, Fig. S10*I*, first vs. fourth row).

### Information Loading in Task-Optimized Nonlinear Networks.

The definition of most amplifying inputs relies on full access to the algebraic form of the dynamics of a network, something that the brain will not have explicitly when performing a working memory task. In turn, the formal equivalence of using the most amplifying input directions to optimal information loading could only be established for networks with linear dynamics receiving instantaneous inputs, while fixing the magnitude of those inputs. Thus, an important question is whether optimizing simple task-relevant cost functions in nonlinear networks (13, 17, 19, 23, 39–41, 58), under only a generic energy constraint (13, 39–41, 58), without explicitly encouraging optimal information loading or non-normality, can be sufficient for such networks to adopt an optimal information loading strategy.

**Fig. 6.**
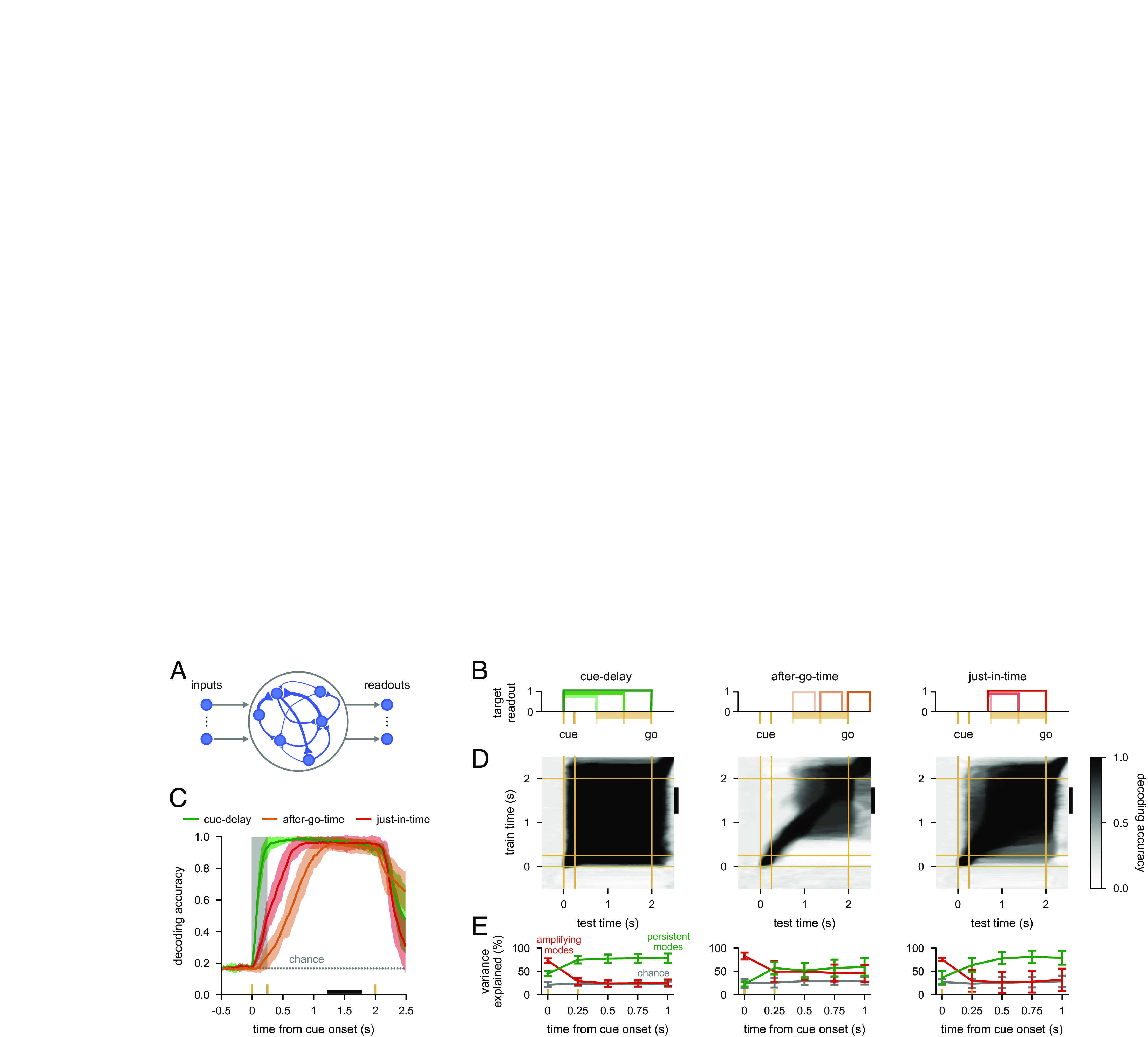
Information loading in task-optimized nonlinear networks. (*A*) Illustration of a recurrent neural network model with unconstrained connectivity (*Middle*). During the cue period, networks received input from one of six input channels on any given trial depending on the cue condition (*Left*). Network activity was decoded into one of six possible behavioural responses via six readout channels (*Right*). All recurrent weights in the network (50 neurons), as well as weights associated with the input and readout channels, were optimized. (*B*) Illustration of cost functions used for training. Yellow ticks indicate cue onset and offset times; yellow bars indicate the range of go times in the variable delay task. Boxcars show intervals over which stable decoding performance was required in three example trials with different delays for each of the cost functions considered: cue-delay (*Left*), after-go-time (*Center*), or just-in-time (*Right*). (*C*) Performance of a delay-trained decoder (the black bar indicates decoder training time period on model neural activity over time in trials with a 1.75-s delay. Yellow ticks show stimulus cue onset, offset, and go times, and the gray shading indicates the cue period. Neural activities were generated by networks optimized for the cue-delay (green), after-go-time (orange), or just-in-time (red) costs. Solid colored lines and shading indicate mean±1 s.d. across 10 networks. The gray dotted line shows chance level decoding. (*D*) Cross-temporal decoding of model neural activity for cue-delay (*Left*), after-go-time (*Center*), and just-in-time (*Right*) trained models. Yellow lines indicate stimulus cue onset, offset, and go times. The black vertical bars on the right of each plot indicate the delay-trained decoder training time period from *C*. (*E*) Percent variance of responses explained by the subspace spanned by either the 25% most persistent (green) or 25% most amplifying (red) modes as a function of time for 20-dimensional linear neural networks fitted to the model neural activities of nonlinear networks optimized for the cue-delay (*Left*), after-go-time (*Center*), or just-in-time cost (*Right*). Gray lines show chance level overlap defined as the expected overlap with a randomly chosen subspace occupying 25% of the full space. Lines and error bars show mean ± 1 s.d. over 10 networks.

We trained nonlinear recurrent networks (Fig. 6*A* and *SI Appendix*, *Materials and Methods*, S2.3.2) on the same memory-guided saccade task as that which our animals performed (Fig. 1*A*). Following previous approaches (13, 39, 40), all recurrent weights in the network, as well as weights associated with the input and read-out channels, were optimized, while only penalizing the average magnitude of neural responses over the course of the whole trial (*SI Appendix*, *Materials and Methods*, S2.3.3).

To study the generality of optimal information loading, we first implemented two standard cost functions that have been widely used in previous work (13, 17, 23, 39, 40). These cost functions required networks to maintain cue information either stably throughout the delay period, starting immediately after cue onset (cue-delay; Fig. 6*B*, *Left*), or only at response time (after-go-time; Fig. 6*B*, *Center*). Both networks achieved high performance, as measured by a late-delay decoder, in line with what their respective cost functions required: immediately after cue onset for the cue-delay cost (Fig. 6*C* and *SI Appendix*, Fig. S11*A*, green), or only shortly before go time for the after-go-time cost (Fig. 6*C*, orange and *SI Appendix*, Fig. S12*B*).

We then further analyzed the dynamics with which these networks achieved competent performance. In particular, we evaluated whether they employed optimal information loading and how well they reproduced critical aspects of the empirical data. The cue-delay network showed signatures of classical attractor dynamics with simple pattern completion: cross-temporal decoding was high at all times, including between the cue and delay periods (Fig. 6*D*, *Left*, cf. Fig. 1*C* and *SI Appendix*, Fig. S1 *A*–*C*), neural activity overlapped strongly between the cue and delay periods (*SI Appendix*, Fig. S11 *C*, *Left*), and at the time of cue offset, neural activity was already very close to its final attractor location in state space (*SI Appendix*, Fig. S11 *D*, *Left*). In line with our theory of optimal information loading, this was achieved by neural activities during the cue period aligning predominantly with the most amplifying modes (Fig. 6*E*, *Left*, red). However, at the same time, activities were also already aligned well above chance with the most persistent modes (Fig. 6*E*, *Left*, green). This was consistent with these networks being explicitly required to exhibit stable coding at all times by the cue-delay cost. These features also made this network a poor match to the experimental data, which showed a combination of dynamic and stable coding and at-chance overlap of activities with the most persistent mode during the cue period (Fig. 5*B*, *C*, and *F*, and *SI Appendix*, Fig. S10 *A* and *B*). We also found similar behavior for networks optimizing a “full-delay” cost, in which cue information must be stably maintained only after cue offset (*SI Appendix*, Fig. S13 and *Materials and Methods* S2.3.3).

At the other extreme, the after-go-time network did not make particular use of attractor dynamics. Instead, it generated largely sequential activities, i.e., pure dynamic coding akin to the dynamics of a feedforward network: cross-temporal decoding was only high at the very end of the delay period (Fig. 6*D*, *Center*, cf. Fig. 1*D* and *SI Appendix*, Fig. S1 *D*, *Right*), neural activity was near-orthogonal between the cue and delay periods (*SI Appendix*, Fig. S12 *D*, *Left*), and these networks did not exhibit attractor states (*SI Appendix*, Fig. S12 *E*, *Left*). This was particularly the case for a fixed delay task, for which this cost function always yielded purely sequential dynamics (*SI Appendix*, Fig. S12 *C*–*E*, *Right*). As required by optimal information loading, neural activities also had a strong initial overlap with the most amplifying modes in this network (Fig. 6*E*, *Center*, green). However, as expected for sequential dynamics, the overlap with the most persistent modes never significantly exceeded that with the most amplifying modes (Fig. 6*E*, *Center*). Again, the apparent lack of attractor dynamics was well explained by the cost function not requiring any stable coding during the delay period. Therefore, this network also deviated from the data in important ways, in this case by failing to exhibit stable coding and high overlap with the persistent mode during the late delay period (cf. Fig. 5*B* , *C*, and *F*, and *SI Appendix*, Fig. S10 *A* and *B*). In summary, network dynamics trained for standard cost functions exhibited optimal information loading and recovered classical network models of working memory (Fig. 1 *C* and *D* and *SI Appendix*, Fig. S1 *A*–*D*) but were different from those seen in experimental recordings (8, 10, 14–16, 24) (Fig. 5*B* , *C*, and *F*).

However, we reasoned that neither of these standard cost functions may be appropriate for understanding PFC function. The cue-delay cost is well justified when stimuli need to be decoded potentially instantaneously after cue onset, and as such, it is most relevant for sensory areas (55). Conversely, the after-go-time cost may be most directly relevant for motor areas, by only requiring stable coding during the short response period (58). Therefore, we also considered a third cost function that required stable coding just in time before the go cue appeared, i.e., during a period that was divorced from the stimulus or response time windows, and as such was more consistent with the putative role of PFC in cognitive flexibility (2, 24) (just-in-time; Fig. 6*B*, *Right*).

In contrast to both standard training costs, just-in-time networks showed the signatures of a combination of attractor and sequential dynamics which were consistent with its cost function. The performance of a late-delay decoder was high only after cue offset but remained so for most of the delay period (Fig. 6*C* and *SI Appendix*, Fig. S11*A*, red), cross-temporal decoding was poor between early and late periods of a trial, but high during the late delay period (Fig. 6*D*, *Right*, *SI Appendix*, Fig. S11*B*, cf. *Center*, Fig. 5*C* and see also *SI Appendix*, Fig. S11*D* for state-space plots), neural activity was near-orthogonal between the cue and delay periods (*SI Appendix*, Fig. S11 *C*, *Right*), and at the time of cue offset, neural activity was far from its final attractor location in state space (*SI Appendix*, Fig. S11 *D*, *Right*). Critically, the overlap of neural activities with the most amplifying and persistent modes showed the characteristic cross-over that we found experimentally (Fig. 6*E*, *Right* and cf. Fig. 5*F*). Thus, this network both used optimal information loading and reproduced the key features of the experimental data. In particular, the requirement for stable coding before the go cue resulted in network dynamics eventually being drawn into attractor states, giving rise to the desired stable coding pattern. At the same time, the fact that no stable coding was required during the cue and early delay period allowed the network to utilize non-normal dynamics, and their most amplifying directions, for reaching those attractor states, thus giving rise to the phenomenon of dynamic coding.

In summary, all task-optimized networks exhibited a key feature of optimal information loading: they made use of most amplifying modes early during the trial (Fig. 6*E*, all red lines start high at 0 s). The extent to which they showed the complete cross-over of amplifying and persistent overlaps predicted by our earlier analyses (Fig. 4*C*, *Center*), and characteristic of the experimental data (Fig. 5*F*), was consistent with how much they were required to exhibit stable coding (8, 10, 11, 14–16). These results suggest that optimal information loading emerges naturally as a dynamical strategy in task-optimized networks, without explicit requirements on their inputs.

## Discussion

While attractor networks have been proposed to underlie a number of core cognitive functions (12, 17, 27–30, 32–34, 42, 48, 59), prominently including working memory (5–7, 28, 30–32, 35), their operation was almost exclusively analyzed in terms of how their intrinsic connectivity supports information maintenance ((5, 7, 12, 28–30, 33, 34, 60); but see refs. (6) and (35), discussed below). We instead studied information loading by external inputs in attractor networks and showed that optimal information loading provides a normative account of the widely observed and puzzling phenomenon of dynamic coding (8, 10, 14–16). Our dynamical analysis also revealed a theoretically grounded aspect of dynamic coding: not only should neural activities during the cue and early delay period be near-orthogonal to those during the late delay period, but they should be orthogonal in the specific directions that are aligned with the most amplifying directions. We found strong evidence for these predictions of optimal information loading in lPFC during a memory-guided saccade task.

Our results unify previous, seemingly conflicting models of working memory maintenance that typically either use attractor dynamics (5, 7, 28) or rely on sequential activities often generated by non-normal dynamics (20, 26, 36, 37). We found that although both classes of models can capture select aspects of neural data (i.e., sequential models can capture early delay activity, whereas attractors are better suited to capturing late delay activity), no model could capture the experimentally observed rich combination of sequential and persistent dynamics (61) (Fig. 1 and see also ref. (39)). We showed that optimal information loading in attractor models with realistic, unconstrained connectivity, leads to the specific combination of sequential and persistent dynamics that has been observed in experiments. Network connectivity being unconstrained was important inasmuch as it allowed for non-normal dynamics—a form of dynamics that is optimal for information maintenance (26, 39) but was not present (or only very weakly) in most previous attractor models of working memory, which used symmetric (or near-symmetric) connectivity (5, 7, 12, 27, 29, 31–34, 43–45, 60). These results generalized across a range of different specific network architectures: using either analytically (Figs. 3 and 4 and *SI Appendix*, Fig. S5 *A* and *B*) or numerically optimized stimulus inputs (*SI Appendix*, Fig. S5 *C* and *D*); and linear integrator (Figs. 3 and 4 and *SI Appendix*, Fig. S5), nonlinear discrete attractor (Figs. 2 and 6, and *SI Appendix*, Figs. S2, S7, and S11–S13) or nonlinear ring attractor dynamics (*SI Appendix*, Fig. S3). The generalizability of these results, in particular even to networks with random connectivity (Figs. 3 *D* and *E* and 4) whose degree of non-normality is known to be limited (54), suggests that network connectivity does not need to be strongly non-normal for dynamic coding to emerge.

In contrast to our optimal information loading-based account, previous attempts to reconcile transient and persistent dynamics specifically proposed that transient dynamics do not affect the delay (or “mnemonic”) coding of the stimulus information (6, 35). These stable delay dynamics are very different from dynamic coding as observed in experiments (3, 8, 10, 11, 14–23), and as predicted by our theory of optimal information loading. Put simply, in previous models, the stimulus input is strongly aligned with the desired persistent state (Fig. 1*D*, *Left*). In real data, and in models that exhibit optimal information loading, stimulus inputs drive network activity strongly orthogonal to the desired persistent state (and specifically in a direction that is aligned with the most amplifying mode) before activity ultimately settles into the correct state (Fig. 1*F*, *Left*). Indeed, previously observed high correlations between cue and delay periods (6), which partially motivates using inputs aligned with the persistent state, are likely due to high overall baseline firing rates, and they have been shown to disappear (and even become negative) when data are mean-centered across cue conditions (8, 23).

There are aspects of the data that were not reproduced accurately by any of the specific models we implemented. First, the overlap with the most amplifying directions became significantly lower than chance over time in the data. This suggests that PFC circuits may be more mathematically “non-normal,” i.e., include stronger effective feedforward loops (20, 26), or excitatory–inhibitory interactions (51) than the networks with randomly chosen or initialised weights we used here (54, 56). (For example, we found that networks with strong feedforward connectivity reproduced this phenomenon; *SI Appendix*, Fig. S8*F*.) Second, the time evolution of the overlaps with the most persistent and most amplifying modes seemed to obey different time constants, with the persistent overlap evolving substantially slower than the amplifying overlap. This may be a result of dynamical transitions between multiple high-dimensional subspaces with graded levels of amplification and persistence, compared to the less complex dynamical transitions that we observed in our models. For example, neural activity appears to quickly rotate out of the most amplifying subspace into an intermediate subspace before it finally slowly enters the persistent subspace later in the delay period. This is clear from Fig. 5*F* because less than 50% of variance of neural activities during the early delay period is captured by both the most amplifying and most persistent modes together. More generally, analysing the data at single trial resolution, as opposed to the across-trial averages we analysed, may provide further important constraints on the underlying circuit dynamics. For example, the seemingly “persistent” neural responses in the late delay period may in part be an artifact of trial averaging with substantial bursting, oscillations, and activity-silent dynamics on individual trials (10, 11, 45, 61).

There have been multiple mechanisms proposed to account for some of the features of the data, most prominently dynamic coding (14, 15), that previously seemed to be at odds with basic attractor network dynamics. These hypothetical mechanisms include short-term plasticity (11, 22, 39, 41), specific changes in the strength of input and recurrent connections (44, 62), and separate stimulus- and delay-responsive cells (3, 10). In addition, the performance of a coordinate transformation between cue-specific sensory representations early in the trial and cue-specific preparatory motor representations later in the trial has also been suggested to account for the transition between dynamic and stable coding over the course of the trial (10, 61, 63). In contrast, we showed that the core phenomenon of dynamic coding emerges naturally, without any of these additional mechanisms, from the same ultimate principle that explains persistent activities (robust memory maintenance implemented by attractor dynamics). Critically, the high initial overlap with the most amplifying modes, which was a core prediction of our theory confirmed by the data and our optimized networks, is not specifically predicted by any of these alternative mechanisms. Nevertheless, these mechanisms are not mutually exclusive to ours. In fact, they might help explain the more nuanced aspects of the data that our specific network implementations did not capture (see above).

A number of recent studies of neural network dynamics have analysed the relationship between the direction of inputs and the magnitude of responses they evoke (51, 55, 56). However, these studies focused on networks with transient dynamics, such as those relevant for perception (55), or motor control (51, 56). In particular, ref. (56) found that optimal inputs (resulting in the largest transients) are typically near-orthogonal to the activity patterns that the network expresses in response to them, providing a normative account for the experimentally observed orthogonality of preparation and execution subspaces in motor cortex (57). Our work suggests that the use of optimal inputs to drive network dynamics, and the orthogonality of those inputs to network responses, is a more general principle of cortical circuits, extending beyond the motor cortex. In particular, our results demonstrate the importance of optimal initialization even when the transients following initialization themselves may be irrelevant, as information is ultimately maintained by stable attractor states.

We predict that optimal initial neural activities, which are aligned with the most amplifying modes, should also align strongly with the slowest or most persistent modes for tasks without a delay period, such as during perceptual decision-making (Figs. 3*C* and 6*B*). Evidence for this regime has been found in the mouse visual cortex for such a task (55): a Henrici’s index of approximately 0.3 was reported (c.f. the >0.8 Henrici’s index in our data), implying the absence of strongly non-normal dynamics and thus a strong overlap between amplifying and persistent modes. In contrast, for brain regions involved in working memory, we predict that optimal information loading should result in distinct amplifying and persistent modes, thus leading to an initial period of dynamic neural activities (aligning with the amplifying modes) followed by more stable neural activities later in the delay period (aligning with the persistent modes). In line with this, a similar combination of dynamic and stable coding that we observed here has also been observed during working memory tasks in both monkey (64, 65) and mouse (36) posterior parietal cortex as well as monkey orbitofrontal and anterior cingulate cortices (8, 19).

We expect our results to also generalize to more cognitively demanding working memory tasks in which, unlike the simple memory-guided saccade task we studied here, the correct response is unknown during the delay period, thus requiring the maintenance of stimulus information before a response can be prepared (14, 15, 22, 23, 61). Indeed, our theory of optimal information loading does not distinguish between information that is being held ready for a response, such as in a delayed response task, or information that is being held for further manipulation later in the trial. (Note that the simple sensory-to-motor coordinate transformation-based accounts of dynamic coding discussed above would not predict generalization to such tasks.) In line with this, strongly dynamic population activity during the cue and early delay period, similar to the dynamics we identified here, has been observed in monkey PFC during such tasks (10, 14–16, 22, 23).

Recurrent neural networks optimized on more complex tasks also exhibited key features of dynamic coding (17, 19, 23, 39, 41)—in line with real neural recordings. In particular, neural activities initially pointed near-orthogonal to the ultimate attractor location in state space (17); the dynamics during the stimulus period had near 0 correlation with late delay activity (23), and cross-temporal decoding of time revealed strongly sequential dynamics in a variety of tasks (19) (see also refs. (39) and (41) for related results). Nevertheless, it remained unclear whether these features of dynamic coding were epiphenomenal or an integral part of the near-optimal functioning of these networks. Our results suggest the latter: that these features were necessary for near-optimal performance. Therefore, optimal information loading likely provides a unifying explanation of dynamic coding during a large variety of working memory tasks.

## Materials and Methods

See our *SI Appendix* file for *Materials and Methods* regarding our models, experimental data, and analysis techniques. We also provide *SI Appendix*, *Text* describing optimal information loading in linear and nonlinear models.

## Supplementary Material

Appendix 01 (PDF)

## Data Availability

Python code data have been deposited in Github (66).
