# Supplementary material for: Optimal information loading into working memory explains dynamic coding in the prefrontal cortex
Source: Proc Natl Acad Sci U S A. 2023 Nov 20;120(48):e2307991120. doi: 10.1073/pnas.2307991120 (PMC10691340; doi:10.1073/pnas.2307991120)
Supplement: Supplementary file 1 — Appendix 01 (PDF) [file pnas.2307991120.sapp.pdf]

Optimal information loading into working memory in prefrontal cortex explains dynamic coding

Jake P. Stroud[1@], Kei Watanabe[2], Takafumi Suzuki[3], Mark G. Stokes[4,5], and Máté Lengyel[1,6]

[1]Computational and Biological Learning Lab, Department of Engineering, University of Cambridge, Cambridge, UK
[2]Graduate School of Frontier Biosciences, Osaka University, Osaka, Japan
[3]Center for Information and Neural Networks, National Institute of Communication and Information Technology, Osaka, Japan
[4]Department of Experimental Psychology, University of Oxford, Oxford, UK
[5]Oxford Centre for Human Brain Activity, Wellcome Centre for Integrative Neuroimaging, Department of Psychiatry, University of Oxford, Oxford, UK
[6]Center for Cognitive Computation, Department of Cognitive Science, Central European University, Budapest, Hungary
[@]corresponding author: j.stroud@eng.cam.ac.uk

Supplementary Information

S1 Supplementary Figures . . . 3

S2 Methods . . . 18

S2.1 Experimental materials and methods 18
S2.2 Neural network models: overview 19
S2.3 Nonlinear networks 22
S2.4 Linear networks 23
S2.5 Previous working memory models 26
S2.6 Canonical nonlinear systems with two stable fixed points 30
S2.7 Analysis methods 31
S2.8 Statistics 35

S3 Supplementary text . . . 36

S4 Comparison of previous models of working memory with task-optimized networks . . . 36

S5 Analysis of linear integrator networks . . . 37

S5.1 Linear integrator networks 38
S5.2 The persistent mode 38
S5.3 The energy of the system 39
S5.4 Optimal information loading: maximising asymptotic persistent overlap with fixed initial norm 39
S5.5 Optimal information loading: achieving fixed asymptotic persistent overlap with a minimal initial norm 40
S5.6 Optimal information loading: maximising asymptotic energy with fixed initial norm 40
S5.7 Optimal information loading: noise-robust decoding 41
S5.8 Optimal information loading: minimising energy with fixed persistent overlap 42

S6 Analysis of canonical nonlinear systems with two stable fixed points . . . 43

S6.1 A local linearization approach based on linearizing around the ground state instead of an attractor 43
S6.2 Most persistent and amplifying directions for nonlinear dynamics 44
S6.3 Locally most amplifying modes predict optimal inputs, analogous to linear networks 44

S7 Analysis of the nonlinear attractor networks of Fig. 2 . . . . . . **45**

S7.1 The relationship between subspaces extracted from nonlinear dynamics and by local linearization 45

S7.2 Locally most amplifying modes predict optimal inputs, analogous to linear networks 45

S7.3 Overlap with most persistent mode in linear networks predicts decoding accuracy in nonlinear networks 45

S7.4 Fitting linear dynamics reveals information loading strategies in nonlinear networks 46

S8 Fisher information in task-optimized ring attractor networks **47**

References **48**

S1 Supplementary Figures

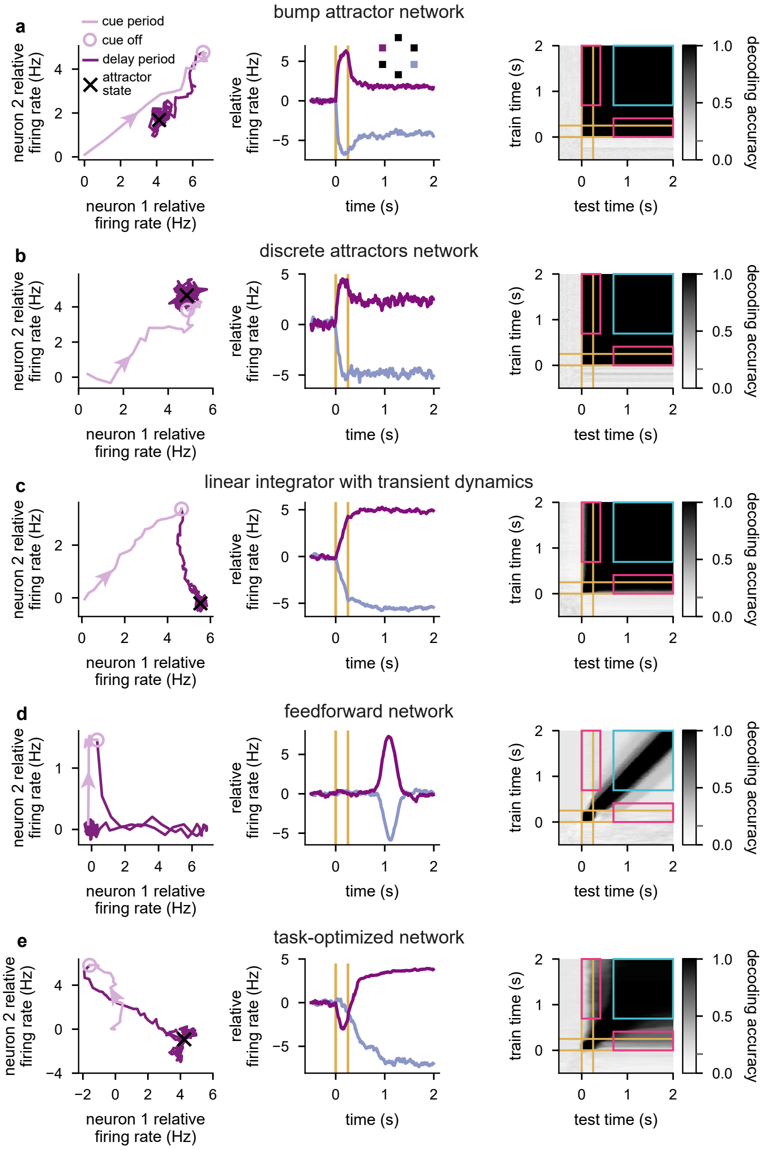

Supplementary Fig. 1 | Dynamics of network models of working memory. a, Neural network dynamics in a bump attractor network (1) performing the task shown in Fig. 1a. Left: trajectory in neural state space in a single cue condition during the cue period (pale purple line, ending in pale purple circle) and delay period (dark purple line). Purple arrow heads indicate direction of travel along the trajectory, black cross shows attractor state. Center: time course of relative (i.e. mean-centered) firing rates of one neuron for two cue conditions (purple vs. blue, see also inset). Yellow lines indicate cue onset and offset times. Right: cross-temporal decoding of neural activity produced by the network across all 6 cue conditions. Pink rectangles indicate generalized decoding between the cue/early delay period and the late delay period and cyan square indicates generalized decoding between time points in the late delay period. The gray tick on the color bar indicates chance-level decoding. **b,** Same as **a** but for a discrete attractors model (1–3). **c,** Same as **a** but for a linear integrator model with transient dynamics that are orthogonal to the attractor subspace (4). **d,** Same as **a** but for feedforward network model (5, 6). **e,** Same as **a** but for a network whose parameters (including recurrent, input, and readout weights) were optimized to perform the task shown in Fig. 1a (cf. Fig. 6d, right).

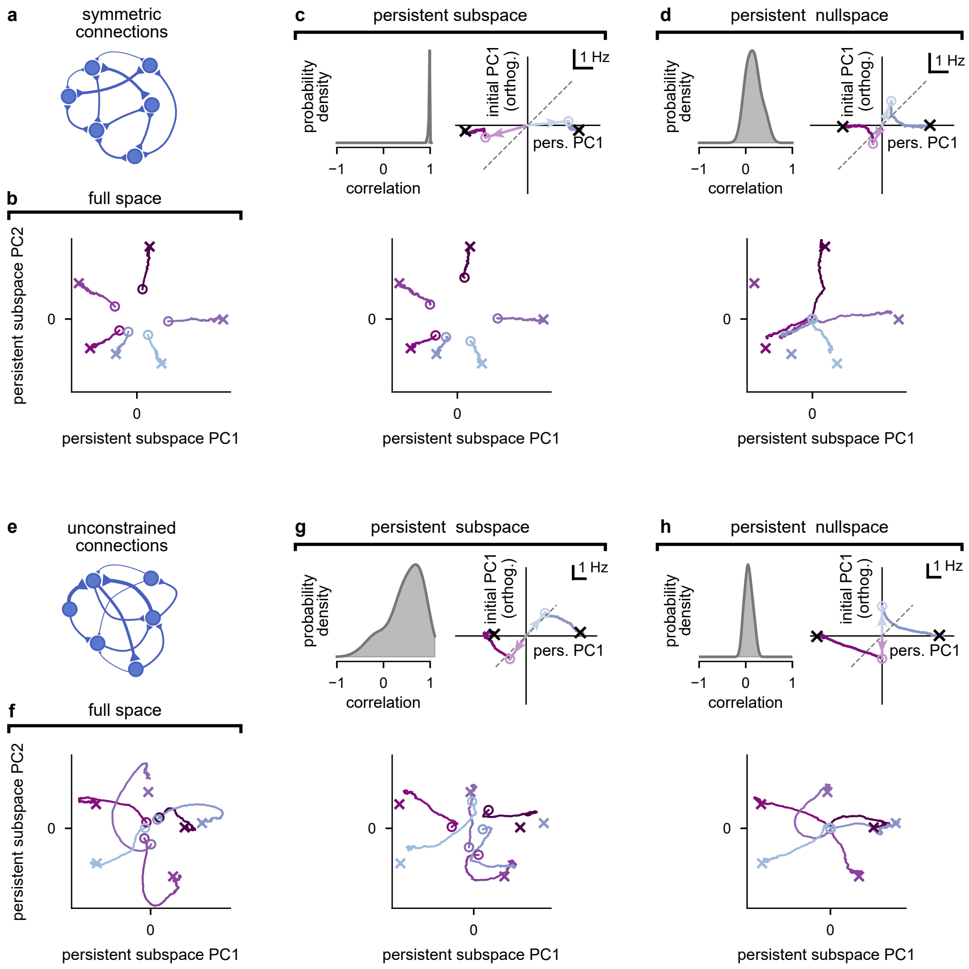

Supplementary Fig. 2 | Attractor network dynamics with or without constraints on the initial condition of the dynamics. **a,** Illustration of an attractor network with symmetric connections. **b**–**d,** Analysis of neural responses in symmetric attractor networks (such as shown in **a**). **b,** Sub-threshold activity (colored trajectories) for all 6 cue conditions (color coded as in Fig. 5e) with initial conditions optimized within the full state space (Methods S2.3.1). Open circles show the optimized initial conditions and crosses show stable fixed points. We show neural activity projected onto the top two principal components of the persistent subspace. **c,** Analysis of neural responses when initial conditions are constrained to lie within the 5-dimensional persistent subspace. Top left: distribution of Pearson correlations between initial and final mean-centered neural firing rates across all 6 cue conditions and 10 networks (same as Fig. 2c, but for persistent subspace-constrained inputs, corresponding to green line in Fig. 2f). Top right: sub-threshold activity for 2 cue conditions in an example network (colored trajectories; same as Fig. 2d, but for persistent subspace-constrained inputs, corresponding to green line in Fig. 2f). Open circles (with arrows pointing to them from the origin) show the optimized initial conditions, black crosses show stable fixed points, dashed gray line is the identity line. Horizontal axis (persistent PC1) shows neural activity projected on to the 1st principal component (PC1) of network activities at the end of the delay period (across the 2 conditions shown), vertical axis (initial PC1 (orthogonalized)) shows projection to PC1 of initial neural activities orthogonalized to persistent PC1. Bottom: same as **b**, but for persistent subspace-constrained inputs, corresponding to green line in Fig. 2f. **d,** Same as **c**, but for persistent nullspace-constrained inputs. Note that the distribution of Pearson correlations of neural firing rates (top left) is distinct from a delta function at 0 because we constrained the initial conditions in the space of sub-threshold activities (rather than firing rates). In the bottom panel, which shows sub-threshold activity, we see that indeed all the colored circles overlap at the origin, indicating near-orthogonality of the initial conditions to the persistent subspace. **e**–**h,** Same as **a**–**d** but for attractor networks without a symmetric connection constraint (i.e. panels **f**, **g**, and **h**, respectively correspond to the networks shown by the black, green, and red lines in Fig. 2). Note initial conditions being near the origin in **f** mean that they are near-orthogonal to the persistent subspace (as in **d**, but without constraining them explicitly to be in the persistent nullspace).

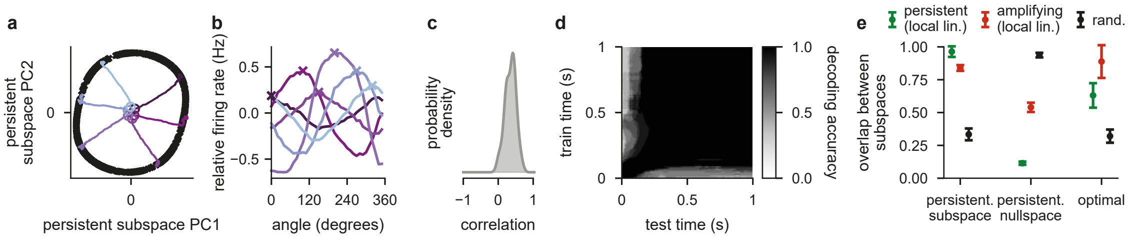

Supplementary Fig. 3 | Dynamics of optimized ring attractor networks. a, Neural activity (colored trajectories) in a ring attractor network with unconstrained connectivity and optimized initial conditions (see Methods S2.3.1 and S2.3.4) for 6 cue conditions (color coded as in Fig. 5e). Open circles show the optimized initial conditions and black crosses show fixed points. We show neural activity projected onto the top two principal components of the persistent subspace. Thus, all circles being near the origin means that initial conditions are near-orthogonal to this subspace (cf. Supplementary Fig. 2f). **b,** Tuning curves at $t = 1$ s for 6 example neurons (colored curves) whose preferred angles (colored crosses) correspond to the 6 cue conditions shown in **a**. **c,** Distribution of Pearson correlations between initial and final mean-centered neural firing rates across the 6 cue conditions and 10 networks (cf. Fig. 2i). **d,** Cross-temporal decoding of neural firing rate activity (cf. Fig. 2k). Note that only the first second of the delay period is shown on both axes because the dynamics of these networks, using a tanh nonlinearity, are faster than those shown in other figures (e.g. Fig. 2), using a ReLu nonlinearity (but the same time constant; Methods S2.2, and Table S1). **e,** Overlap (mean$\pm$1 s.d. across 10 networks) of the 2 locally most persistent (green), most amplifying (red), or random directions (black), obtained using a local linearization around the origin, with the 'persistent subspace' and 'persistent nullspace' of the original nonlinear dynamics, obtained without linearization, and the subspace spanned by the 'optimal' initial conditions of the original nonlinear dynamics (cf. Supplementary Fig. 7a, bottom; see Methods S2.4.2 and S2.7.3). We used 2-dimensional subspaces from the local linearization because we found empirically that the ring attractor lay in a 2-dimensional subspace (see also **a**).

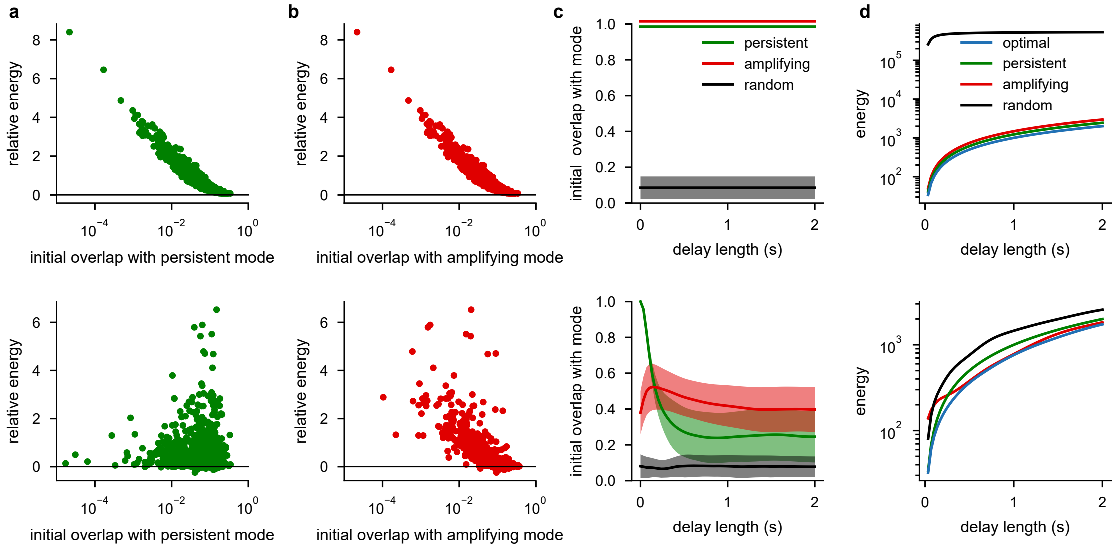

Supplementary Fig. 4 | Analysis of the total energy produced by different initial conditions in linear networks. **a,** The norm of neural activity integrated over time (i.e. a measure of total energy used by the network) for each of 1000 random initial conditions (10 initial conditions for each of 100, 100-neuron networks) relative to the energy produced by the most amplifying initial condition, plotted as a function of their overlap with the persistent mode for symmetric (top) and unconstrained (bottom) linear integrator networks. A positive value on the y-axis means that the total energy produced by the given random initial condition is greater than that produced by the most amplifying initial condition. Initial conditions are scaled so that they all produce the same level of persistent activity (i.e. the same level of performance) after 2 s of simulation. **b,** Same as **a**, but initial conditions are plotted as a function of their overlap with the most amplifying mode. Note that overlap with the most amplifying mode (but not in general with the most persistent mode) is strongly predictive of total energy (with an inverse relationship between the two). **c,** Overlap (mean$\pm$1 s.d. across the 100 networks from **a** and **b**) of optimal initial conditions (Eq. S74), producing an overlap of 1 with the persistent mode after a given delay length (x-axis) while using the minimal total energy over time (Eq. S73), with either persistent (green), most amplifying (red), or random (black) directions, for symmetric (top) and unconstrained (bottom) networks. In unconstrained networks, for very short delay lengths, initial conditions must align exactly with the persistent mode, by necessity (green lines at 0 s). For longer delay lengths, initial conditions make greater use of the most amplifying direction (red lines). **d,** Total energy (mean across the 100 networks from **a** and **b**; we do not show error bars for visual clarity) for dynamics starting from initial conditions that produce an overlap of 1 with the persistent mode after a given delay length (x-axis) in symmetric (top) and unconstrained (bottom) networks. Initial conditions were chosen to be optimal (blue; i.e. using the least energy, cf. panel c), or aligned with the most persistent (green), most amplifying (red), or a random direction (black). In unconstrained networks, for very short delay lengths, initialising along the most persistent mode achieves near-optimal energy-efficiency (green is close to blue), but for longer delay lengths, initialising along the most amplifying mode becomes more energy efficient (red is closer to blue). (Note that for symmetric networks, top, we have offset the curves for the most amplifying, persistent, and optimal directions because these 3 directions are the same and therefore produce the same total energy.)

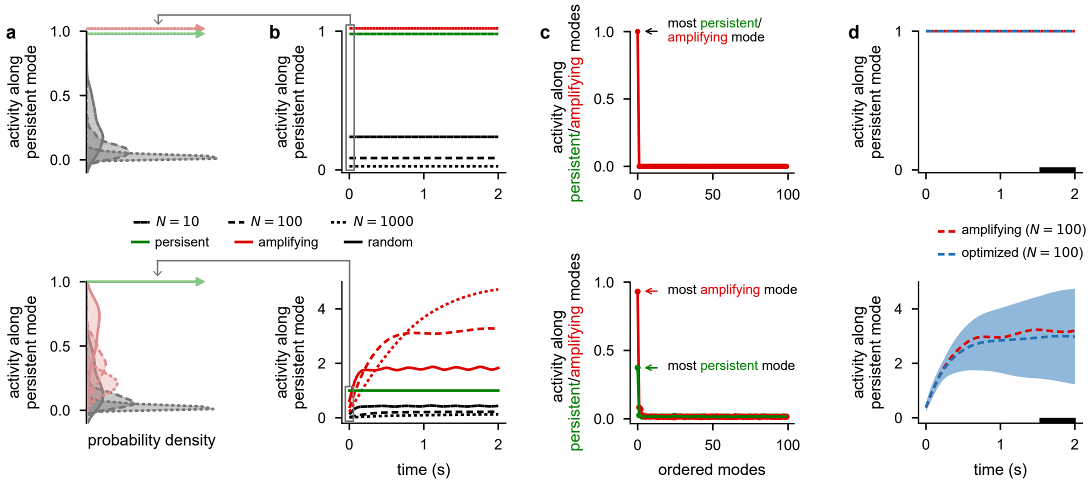

Supplementary Fig. 5 | Analysis of linear networks of different sizes. **a,** Distributions of absolute overlap with the persistent mode for persistent (pale green), most amplifying (pale red), or random initial conditions (gray) across 100 randomly sampled linear symmetric (top) and unconstrained networks (bottom) consisting of either 10 (solid), 100 (dashed), or 1000 (dotted) neurons (cf. Fig. 3d). The persistent initial conditions produced delta functions at 1 (arrows). Results for persistent and most amplifying initial conditions are identical in symmetric networks (top). **b,** Time course of mean (across the 100 networks from **a**) absolute overlap with the persistent mode when starting network dynamics from persistent (green), most amplifying (red), or random initial conditions (black) in symmetric (top) and unconstrained networks (bottom) consisting of either 10 (solid), 100 (dashed), or 1000 (dotted) neurons (cf. Fig. 3e). Results for persistent and most amplifying initial conditions are identical in symmetric networks (top). **c,** Mean (across 100 networks) overlap of initial conditions that were optimized so as to generate maximal persistent activity in 100-neuron noisy symmetric (top) and unconstrained (bottom) networks with 100 orthogonal modes ordered by their persistence (green) or amplification (red) (i.e. corresponding to the rank ordered eigenvectors of the weight matrix, green, or of the observability Gramian of the dynamics, red; Methods S2.7.1). In symmetric networks (top), the optimized initial conditions overlap only with the most amplifying mode and no other mode (note that the most persistent mode is identical to the most amplifying mode in this case). In unconstrained networks (bottom), optimized initial conditions overlap strongly with the most amplifying mode and only weakly with other modes. (The non-zero overlap with the most persistent mode is simply due to the fact that there is a non-zero overlap between the most persistent and amplifying mode in random networks, and it is at the level that would be expected based on this overlap.) **d,** Time course of mean (across 100 networks) absolute overlap with the persistent mode for the same 100-neuron noisy symmetric (top) and unconstrained networks (bottom) as those shown in **c** when the network is started from optimized initial conditions (blue), and for comparison for the most amplifying (red dashed) initial conditions (cf. Fig. 3e). Note the close agreement between the two indicating that the most amplifying mode is indeed optimal in these networks. Horizontal black bar on x-axis shows the time period in which we applied the cost function to optimize the initial conditions (Methods S2.7.3).

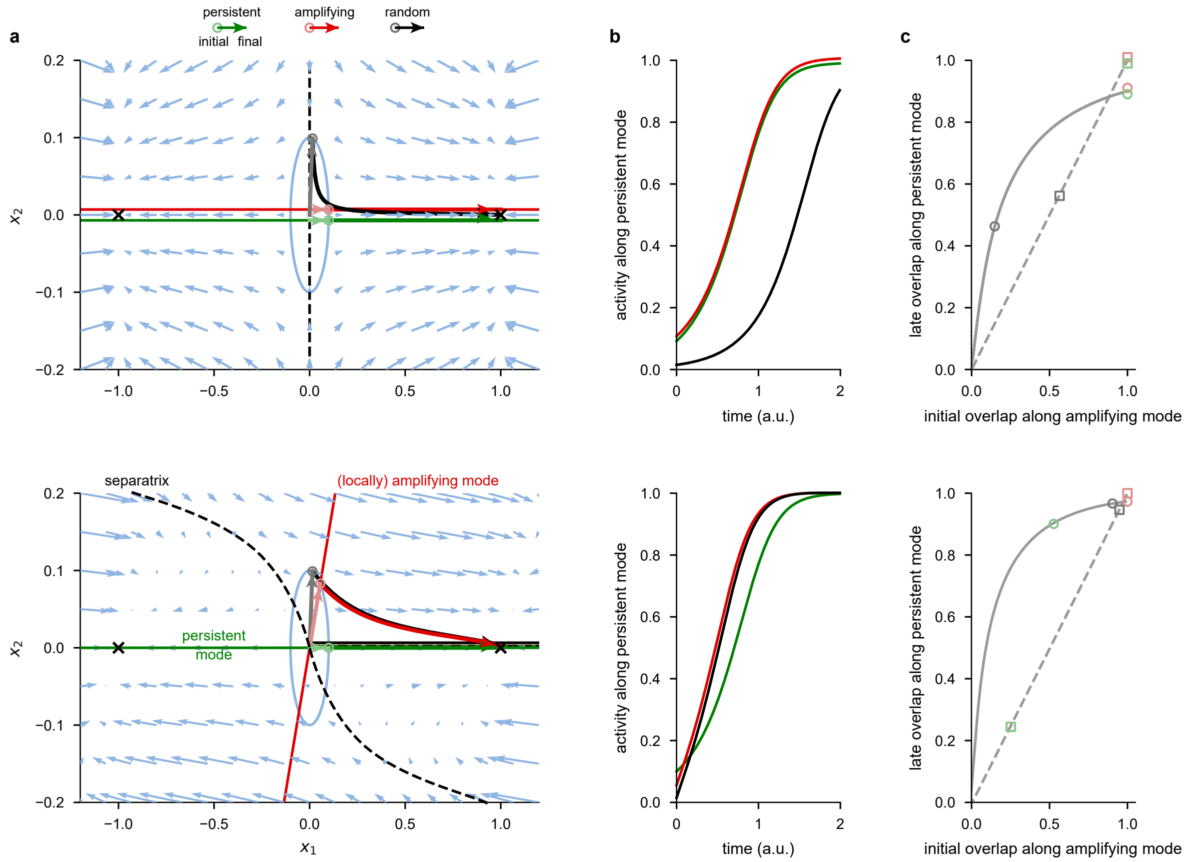

Supplementary Fig. 6 | Analysis of canonical nonlinear attractor systems. a, State space of a canonical nonlinear system with two attractors and a symmetric (top) and non-symmetric Jacobian (bottom, see also Methods S2.6, Section S6; cf. Fig. 3b). Pale blue arrows show flow field dynamics (direction and magnitude of movement in the state space as a function of the momentary state). Black crosses indicate asymptotically stable fixed points (i.e. attractor states), dashed black line shows the separatrix (the manifold separating the basins of attraction of the two attractors). Thin green and red lines indicate the locally most persistent and amplifying modes around the origin, respectively (lines are offset slightly in the top panel to aid visualisation). Pale green, red, and gray arrows with open circles at the end indicate most persistent, amplifying, and random initial conditions, respectively. Blue ellipses show the fixed initial condition norm around the origin to highlight the different axis scales. Dark green, red, and black arrows show neural dynamics starting from the corresponding initial condition. **b,** Time course of dynamics of the system along the persistent mode (i.e. the projection onto the green line in **a**) when started from the persistent (green), most amplifying (red), or random (black) initial conditions for the symmetric (top) and the unconstrained system (bottom). **c,** Late overlap with the locally persistent mode as a function of initial overlap with the locally most amplifying mode in the canonical nonlinear systems shown in panels **a**–**b** (solid gray line) and, for comparison, in the linear networks of Fig. 3a–c (dashed gray line) for symmetric (top) and unconstrained systems (bottom). Late overlap is measured as the mean overlap of activity along the persistent mode (panel **b**, from $t = 0.8$ to $t = 2$ for the canonical nonlinear system; Fig. 3c, from $t = 0.8$ s to $t = 2$ s for the linear networks). Open circles and squares indicate the random (gray), persistent (pale green), and most amplifying (pale red) initial conditions used respectively in panels **a** and **b** for the canonical nonlinear system, and in Fig. 3b–c for the linear networks.

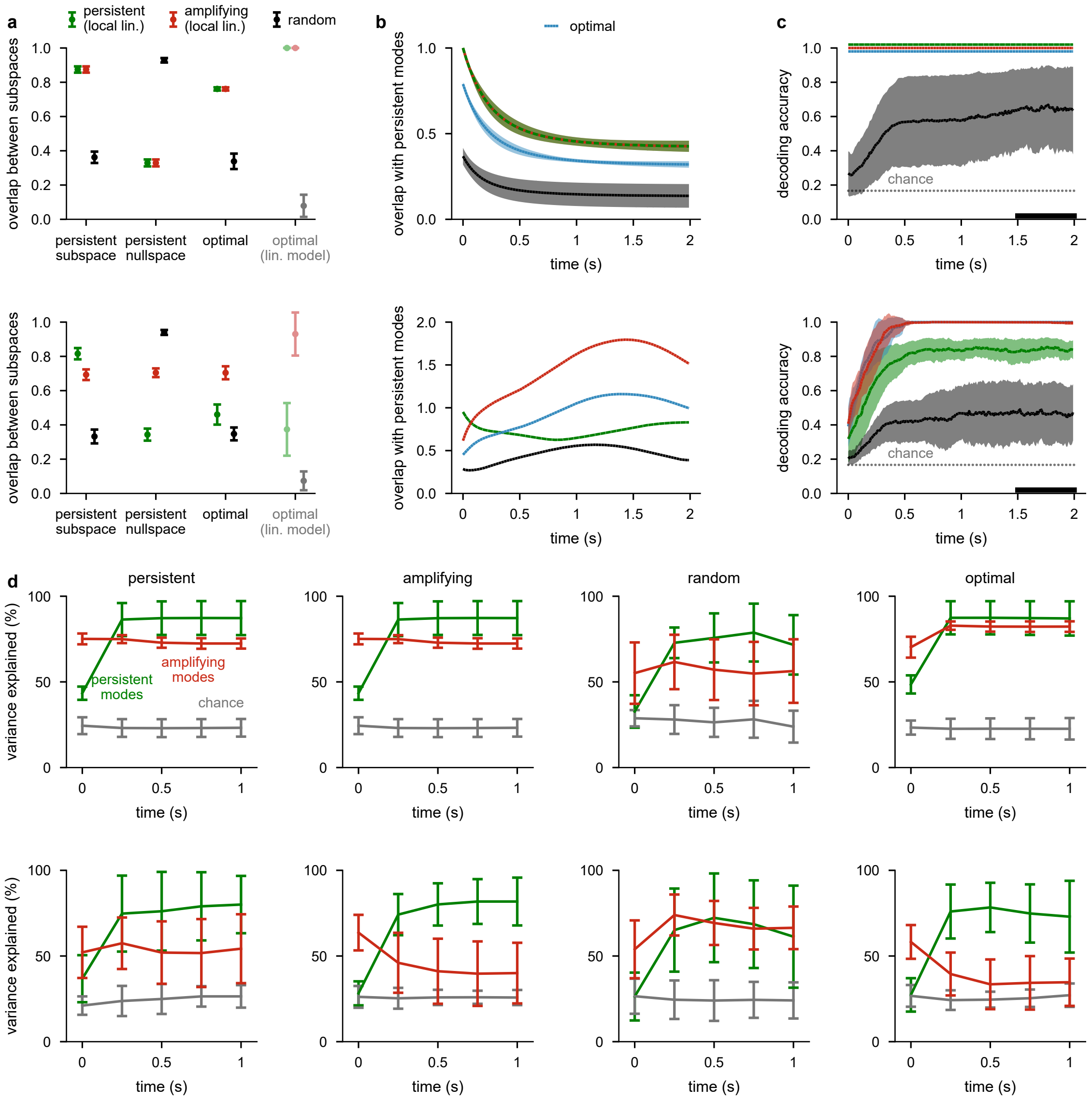

Supplementary Fig. 7 | Linear analyses of the nonlinear attractor networks of Fig. 2. a, Overlap (mean±1 s.d. across 10 networks) of the 5 locally most persistent (green), most amplifying (red), or random directions (black) of the symmetric (top) and unconstrained (bottom) networks from Fig. 2, obtained using a local linearization around the origin, with the 'persistent subspace' and 'persistent nullspace' of the original nonlinear dynamics, obtained without linearization (as used in Fig. 2f and l, red and green), and the 5-dimensional subspace spanned by the 6 'optimal' initial conditions of the original nonlinear dynamics (used in Fig. 2b–e, h–k, and f and l, black). For comparison, we also show the overlap (mean±1 s.d. across 100 networks) of the single most persistent (pale green), most amplifying (pale red), and random (gray) direction with the optimal initial condition of the linear networks from Supplementary Fig. 5c,d ('optimal (lin. model)'). **b,** Time course of the overlap (mean±1 s.d. across 10 networks, s.d. only shown in top panel for visual clarity) of the linearized dynamics of symmetric (top) and unconstrained networks (bottom) with the subspace spanned by their most persistent modes when started from initial conditions that were optimized for the decoding accuracy of the nonlinear dynamics while constrained to be within the locally most persistent (green), most amplifying (red), or a random subspace (black). The linear dynamics, the persistent subspace wrt. which overlap is measured, and the subspaces within which initial conditions were constrained while being optimized, were all based on a local linearization of the nonlinear dynamics around the origin. Compare with Fig. 3e for the analogous plots for linear networks. For reference, blue line shows overlap of the same linearized dynamics when started from the initial conditions directly optimized for the decoding accuracy of the nonlinear dynamics without subspace constraints (used in Fig. 2b–e, h–k, and f and l, black). For consistency with Fig. 3b–e (where initial conditions were constrained to have unit norm), we scaled activity by the norm of the initial condition (which was constrained to be 3 here; Methods S2.4.2). **c,** Performance (mean±1 s.d. across 10 networks) of a delay-trained decoder (black bar indicates decoder training time period; Methods S2.7.4) on neural activity in stochastic nonlinear symmetric (top) and unconstrained networks (bottom) over time. Colors indicate initial conditions as in **b**. (Blue line shows same data as black line in Fig. 2f and l). Gray dotted line shows chance level decoding. Green, red, and blue lines are vertically offset slightly in the top panel to aid visualization. Compare with Fig. 4a (noise matched) for the analogous plots for linear networks (though with non-instantaneous inputs). *(Caption continued on next page.)*

Supplementary Fig. 7 | Linear analyses of the nonlinear attractor networks of Fig. 2 (cont'd). d, Percent variance of responses explained (mean±1 s.d. across 10 networks) by the subspace spanned by either the 25% (i.e. 5) most persistent (green) or 25% (i.e. 5) most amplifying (red) modes as a function of time for 20-dimensional linear neural networks fitted to the neural responses generated by the symmetric (top) and unconstrained (bottom) nonlinear networks when started from the same (optimized) initial conditions analyzed in **b**–**c**: constrained to be within the locally most persistent (far left), most amplifying (center left), or a random subspace (center right), as determined by the local linearization of the dynamics, or without subspace constraints (far right). Gray lines show chance level overlap defined as the expected overlap with a randomly chosen subspace occupying 25% of the full space (i.e. 5 dimensions). Compare with Fig. 4c for the analogous plots for linear networks (though with non-instantaneous inputs, and performance-matched levels of noise, see also Section S7) and with Fig. 5f and Supplementary Fig. 10f,g for analogous plots of linear neural networks fitted to experimental data.

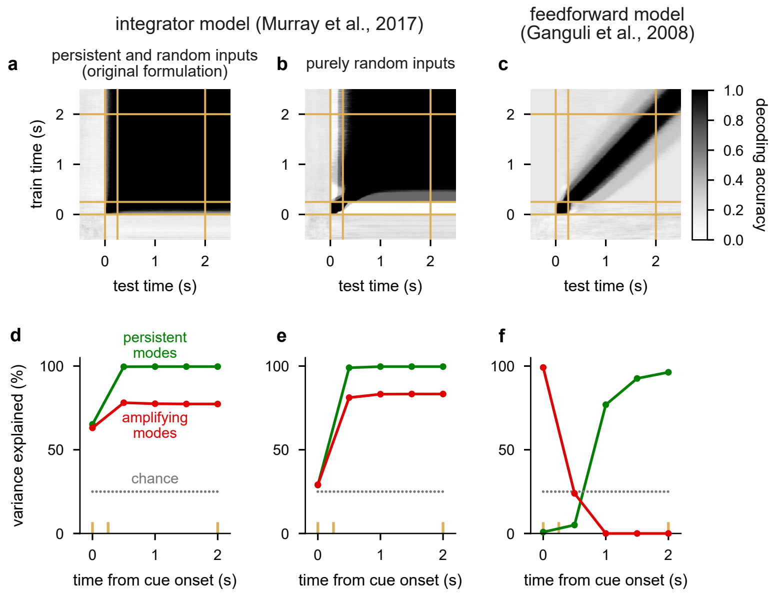

Supplementary Fig. 8 | Analysis of two variants of an integrator model and feedforward model. **a,** Cross-temporal decoding of model neural activity (cf. Fig. 2e,k, Fig. 4b, and Fig. 5c) for a linear integrator model (4) (cf. Supplementary Fig. 1c and Methods S2.5). Yellow lines indicate cue onset, offset, and go times. **b,** Same as **a** for the same model but for inputs aligned with purely random directions (as opposed to inputs aligned with both persistent and random directions as in the original formation of Ref. (4)). **c,** Same as **a** but for a linear feedforward network model (5, 6) (cf. Supplementary Fig. 1d). **d,** Percent variance of responses explained by the subspace spanned by either the 25% most persistent (green) or 25% most amplifying (red) modes as a function of time for the linear integrator model from **a** (cf. Fig. 4c,b, Fig. 5f, and Fig. 6e). Yellow lines indicate cue onset, offset, and go times. Gray dotted line shows chance level overlap with a subspace spanned by 25 random orthogonal directions. **e,** Same as **d** for the same model but for inputs aligned with purely random directions. **f,** Same as **d** but for a linear feedforward network model (5, 6).

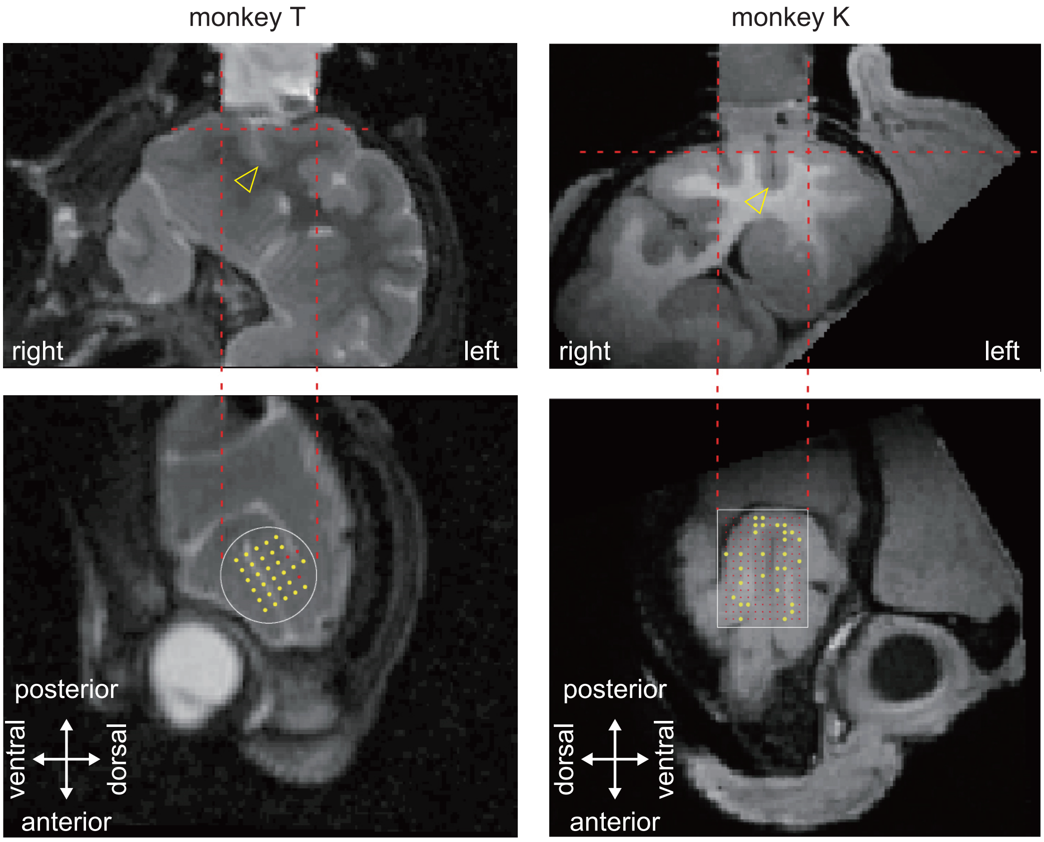

Supplementary Fig. 9 | Recording locations for the two monkeys. Left: recording locations in monkey K (T1-weighted image). In order to image the interior of the chamber, we filled the chamber with cut cottons soaked in iodine. In the upper picture, the yellow arrow indicates the principal sulcus. In the bottom picture, locations of the 11 by 15 grid holes were superimposed over the MR picture. Right: recording locations in monkey T (T2-weighted image). The bottom picture shows the location for the grid of the 32 semi-chronic electrodes. Yellow dots indicate electrode penetrations and recording sites, red dots indicate non-visited sites.

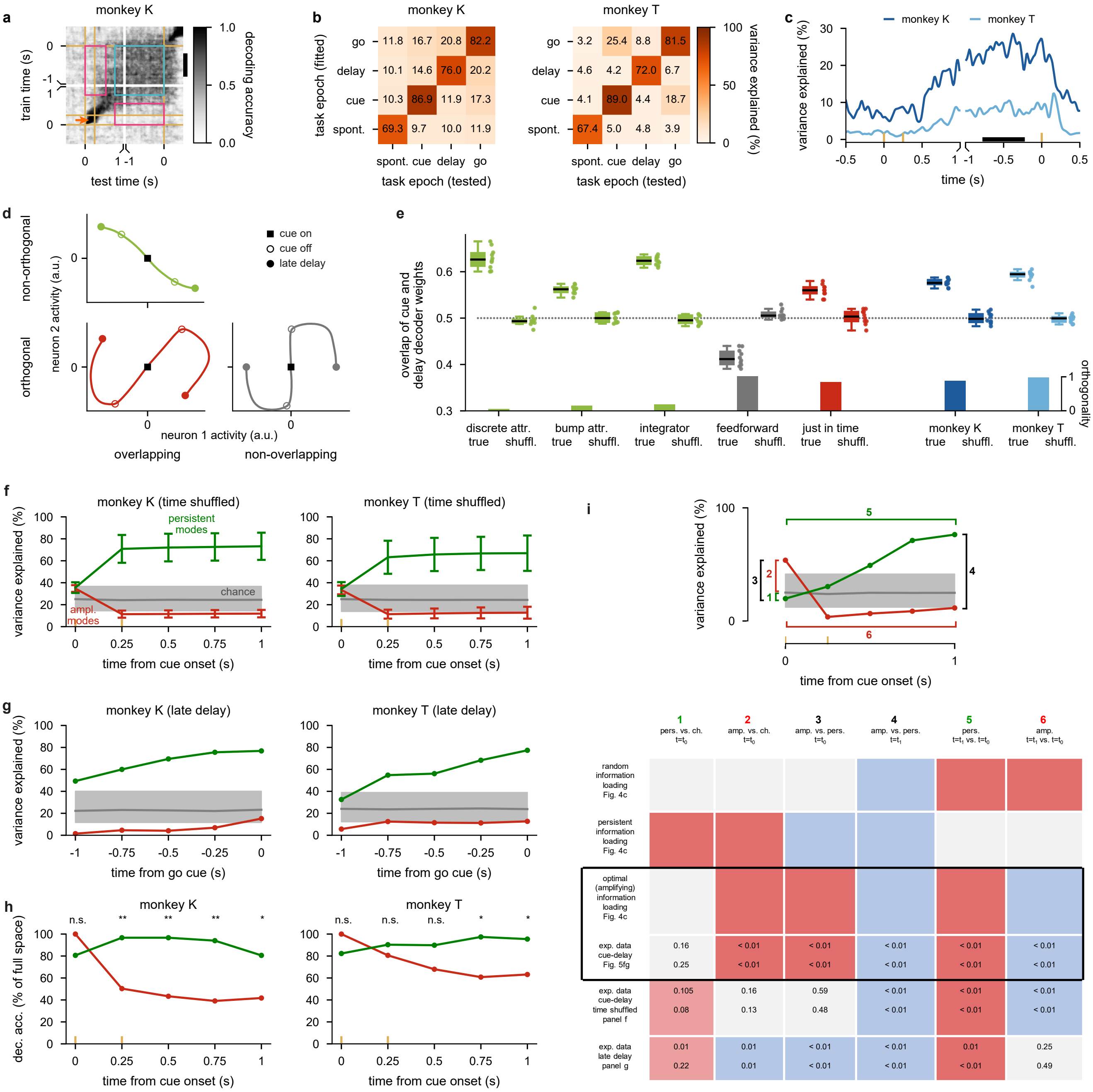

Supplementary Fig. 10 | Supplemental analysis of experimental data and comparison to models. **a,** Cross-temporal decoding analysis for monkey K (cf. Fig. 5c for the same analysis for monkey T and for explanation of plotting scheme and annotations). **b,** Subspace overlap between different task epochs, measured as the percent variance explained (PVE) by projecting neural activity from one task epoch (tested) through the top 10 PCs of another task epoch (fitted). Diagonal elements show the PVE within each task epoch. We show results for monkey K (left) and monkey T (right). **c,** Time course of overlap with delay epoch subspace, measured as the percent variance explained by the top 2 PCs obtained from delay period activity (black bar shows time period of activity from which these PCs were obtained) on held-out test data taken in different time bins. This metric is called the alignment index (7) and is very similar to that used in Ref. 4 (Methods S2.7.3). We show mean (over 10 different data splits) results for both monkeys. Yellow ticks on horizontal axis indicate cue onset, cue offset, and go times. *(Caption continued on next page.)*

Supplementary Fig. 10 | Supplemental analysis of experimental data and comparison to models (cont'd). d, Schematic of 3 different hypothetical scenarios for the relationship between cue and late delay activities (panels), illustrated in neural dynamics for 2 neurons and 2 cue conditions. Colored traces show neural trajectories, black squares indicate cue onset, open circles indicate cue offset, and filled circles show late delay activity. Left vs. right: populations encoding the cue during cue and late delay periods are overlapping vs. non-overlapping, respectively. Top vs. bottom: cue and delay activities are non-orthogonal vs. orthogonal, respectively. (Note that we are not showing dynamics for non-overlapping, non-orthogonal dynamics because no overlap necessarily implies orthogonality.) **e,** Relationship between cue and late delay activities in various different models and our experimental recordings (x-axis). Top: population overlap measured as the mean difference between cue and delay epoch decoder weights (left for each model and data) and, as a control, when randomly shuffling decoder weights across neurons (right for each model and data) (Methods S2.7.6). Box plots show medians (black lines), quartiles (boxes), and 1.5 times the inter-quartile range (whiskers). Dotted gray line shows chance level overlap. Bottom: orthogonality measured as 1 minus the mean overlap between cue and delay epochs (given by the corresponding elements of the subspace overlap matrices shown in panel **b** and Supplementary Fig. 11c, center right). The discrete attractors, bump attractor, and integrator models show high overlap but low orthogonality. The simple feed-forward network shows high orthogonality but low overlap (note that recurrent networks with embedded feed-forward connectivity (5) may show high overlap). The just-in-time network shows high overlap and orthogonality, similar to the experimental data in both monkeys. **f–g,** Same analysis as in Fig. 5f, but either after randomly shuffling data across time (but consistently across conditions and neurons, and applied to the same time period as in the main analysis; **f**, see also Methods S2.4.3), or applied to the late delay time period (without across-time shuffling) in which we do not expect information loading dynamics (**g**). **h,** Decoding of stimulus information within the subspace spanned by either the 25% most persistent modes (green), or the 25% most amplifying modes (red) in the linear neural networks shown in Fig. 5f relative to decoding accuracy using the full space. Comparisons use two-sided permutation tests (*, $p < 0.05$; **, $p < 0.01$; n.s., not significant; see Methods S2.8) **i,** Top inset: original data analysis of overlaps repeated from Fig. 5f to indicate the comparisons (colored numbers) we show in the table below (numbered columns). Bottom: table showing p-values (in each cell for experimental data, top: monkey K, bottom: monkey T) from two-sided permutation tests for each comparison of the main analysis (row 4, repeated from the main text associated with Fig. 5f) and the control analyses shown in panels **f** and **g** of this figure (rows 5–6). Top 3 rows show predictions for the sign of each comparison under different information loading strategies in unconstrained linear networks (Fig. 4c): using inputs aligned with random directions (1st row), persistent directions (2nd row), or the most amplifying directions (3rd row). In the column headings, pers., amp., and ch. respectively refer to overlap with most persistent, most amplifying and random subspaces (chance), t_0 refers to the beginning of the analysis time window, i.e. cue onset (rows 1–5) or 1 s before the timing of the go cue (row 6), and $t_1 = t_0 + 1$ s refers to the end of the analysis time window. The colored numbers above each column correspond to the comparisons shown in the inset above the table. Gray indicates no significant difference between data points, red and blue indicate a significant difference for both monkeys where the first data point is respectively greater or smaller than the second data point, and pale red indicates a significant difference for one of the two monkeys (see Methods S2.8).

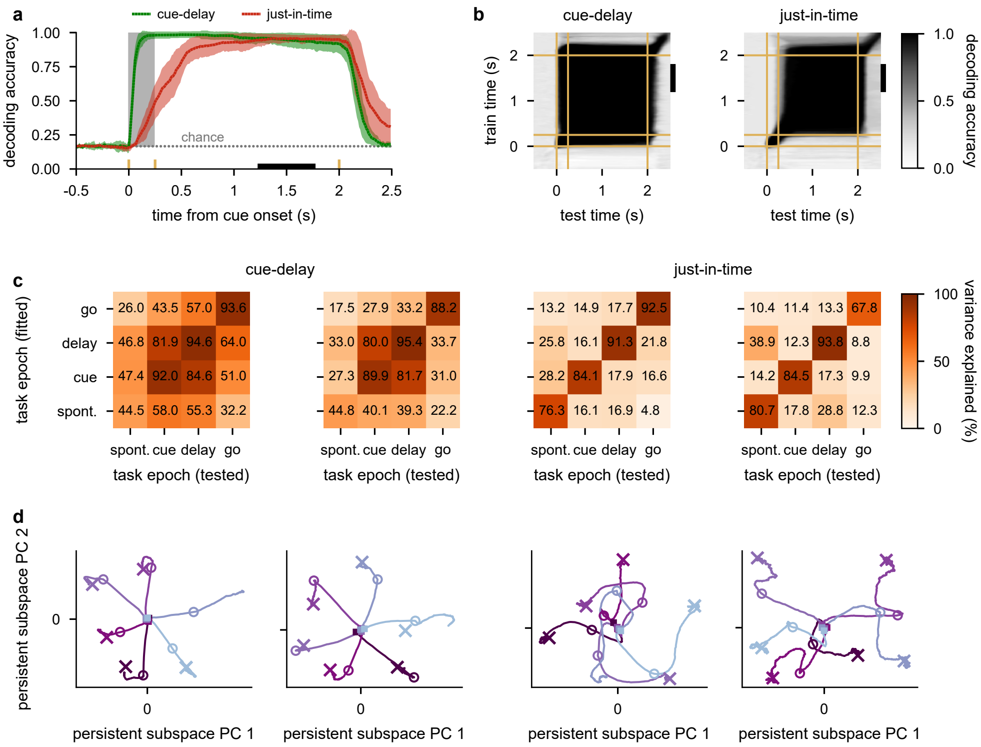

Supplementary Fig. 11 | Cue-delay and just-in-time trained networks. **a–b,** Same as Fig. 6c green and red, and Fig. 6d left and right, but with a regularisation strength of $\alpha^{(2)}_{\text{nonlin}} = 0.0005$ used during training (Methods S2.3.2). **c,** Subspace overlap between different task epochs, measured as the percent variance explained (PVE) by projecting neural activity from one task epoch (tested) through the top 4 PCs of another task epoch (fitted; cf. Supplementary Fig. 12d, Supplementary Fig. 13d, and Supplementary Fig. 10b). Diagonal elements show the PVE within each task epoch. We show results for cue-delay (left two panels) and just-in-time trained networks (right two panels) trained with either a regularisation strength of $\alpha^{(2)}_{\text{nonlin}} = 0.00005$ (left panel for each model, as in Fig. 6) or $\alpha^{(2)}_{\text{nonlin}} = 0.0005$ (right panel for each model, as in panels **a**–**b**). **d,** Neural activity plotted in the top two PCs of delay-epoch activity for all 6 initial conditions for cue-delay and just-in-time trained networks for each of the network-regularization combinations shown in **c** (cf. Supplementary Fig. 2b–d and f–h.) Purple traces show state-space trajectories, squares indicate cue onset, open circles indicate cue offset, and crosses indicate asymptotically stable fixed points, colors indicate cue condition as in Fig. 5e.

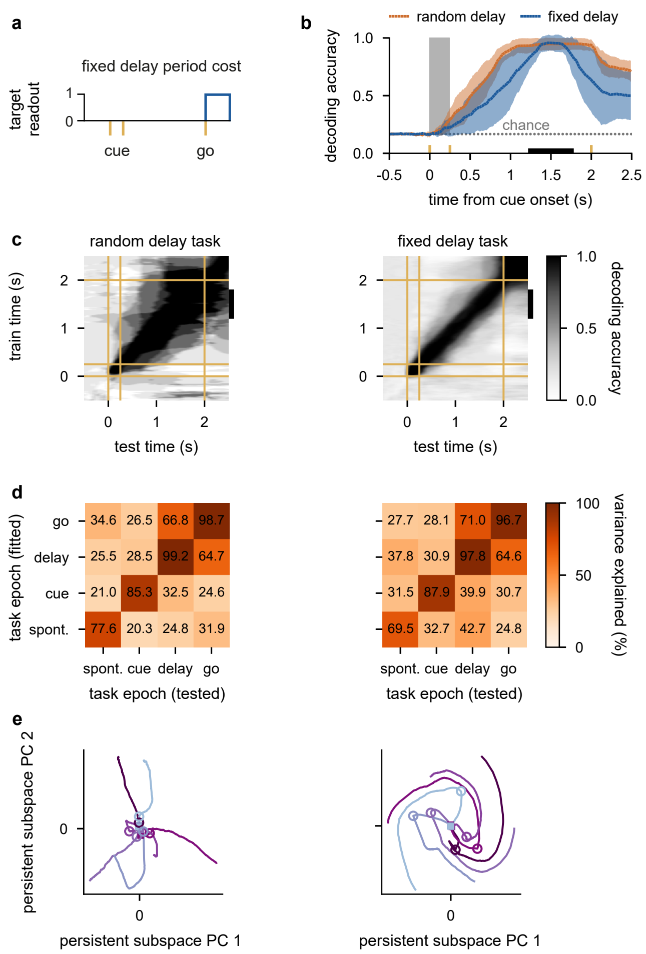

Supplementary Fig. 12 | After-go-time trained networks. **a,** Cost function for after-go-time training on the fixed delay task (Methods S2.3.3). Cue onset, cue offset, and go cue times are indicated by the yellow vertical lines. The boxcar shows the interval over which stable decoding performance was required (i.e. the cost was only applied after the go cue). **b–c,** Same as Fig. 6c orange and Fig. 6d center, but with a regularisation strength of $\alpha^{(2)}_{\text{nonlin}} = 0.0005$ used during training and when either a random (**b** orange, **c** left) or a fixed delay task is used (**b** blue, **c** right, Methods S2.7.4). **d,** Subspace overlap between different task epochs, measured as the percent variance explained (PVE) by projecting neural activity from one task epoch (tested; cf. Supplementary Fig. 11c, Supplementary Fig. 13d, and Supplementary Fig. 10b) through the top 4 PCs of another task epoch (fitted) for the networks shown in **b**–**c**. Diagonal elements show the PVE within each task epoch. **e,** Neural activity plotted in the top two PCs of delay-epoch activity for all 6 initial conditions for random delay (left) and fixed delay (right) trained networks (cf. Supplementary Fig. 2b–d and f–h; and Supplementary Fig. 11d.) Purple traces show state-space trajectories, squares indicate cue onset, open circles indicate cue offset, and colors indicate cue conditions as in Fig. 5e. (Note that the absence of crosses indicates the absence of asymptotically stable fixed points.)

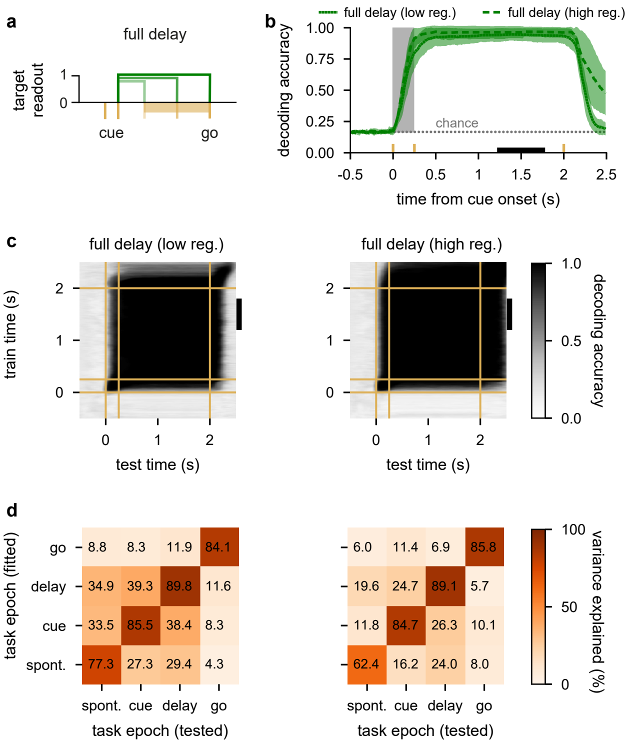

Supplementary Fig. 13 | Full-delay trained networks. a, Cost function for full-delay training on the random delay task (Methods S2.3.3). Yellow ticks indicate cue onset and offset times, the yellow bar indicates range of go times in the variable delay task. Boxcars show intervals over which stable decoding performance was required in three example trials with different delays (Methods S2.3.3). **b–c,** Same as Fig. 6c–d, but when training with the full-delay cost with a regularisation strength of $\alpha^{(2)}_{\mathrm{nonlin}} = 0.00005$ (**b** solid, **c** left) or $\alpha^{(2)}_{\mathrm{nonlin}} = 0.0005$ (**b** dashed, **c** right, Methods S2.7.4). **d,** Subspace overlap between different task epochs, measured as the percent variance explained (PVE) by projecting neural activity from one task epoch (tested; cf. Supplementary Fig. 11c, Supplementary Fig. 12d, and Supplementary Fig. 10b) through the top 4 PCs of another task epoch (fitted) for the networks shown in **b**–**c**. Diagonal elements show the PVE within each task epoch.

S2 Methods

S2.1 Experimental materials and methods

Experimental methods have been described before (8) and largely followed those used in our previous publications (9–11). We briefly summarize the methods below.

S2.1.1 Subjects and apparatus

We used two female macaques (monkey T, *Macaca mulatta*, 5 kg; monkey K, *Macaca fuscata*, 8 kg). Both monkeys were housed individually. Prior to this study, Monkey K participated in another prefrontal neural recording study with different objectives using different tasks from the present study (12). Monkey K was labelled as monkey C in that prior study. The light/dark cycle was 12/12 hr. (light, from 8:30 a.m. to 8:30 p.m.). The monkeys sat quietly in a primate chair in a dark, sound-attenuated shield room. During both training and neural recording sessions, we restrained the monkeys' head movement non-invasively using a thermoplastic head cap as described in (13). This head cap is made of a standard thermoplastic splint material (MT-APU, 3.2 mm thick, CIVCO Radiotherapy, IA., USA), and was molded out so that it conformed to the contours of the animals' scalp, cheek bone, and occipital ridge. Visual stimuli were presented on a 17 inch TFT monitor placed 50 cm from the monkeys' eyes. Eye movements were sampled at 120 Hz using an infrared eye tracking system (ETL-200, ISCAN, MA.). Eye fixation was controlled within a 6.5° imaginary square window. TEMPO software (Reflective Computing, WA.) was used to control behavioral tasks. All experimental procedures were approved by the Animal Research Committee at the Graduate School of Frontier Biosciences, Osaka University, Japan and were in full compliance with the guidelines of the National BioResource Project 'Japanese Macaques'. Experimental work performed in non-human primates that was not funded by Wellcome may not adhere to the principles outlined in the NC3Rs guidance on Non-human Primate Accommodation, Care and Use.

S2.1.2 Behavioral task

The monkeys were trained on a memory-guided saccade task requiring them to remember the location of a visual stimulus cue on a screen and to make a correct eye movement after a delay period (Fig. 1a). Specifically, this task required monkeys to fixate on a central ring for a period of 2.6–7.4 s followed by a stimulus cue (a white square) appearing in one of six pre-determined locations for 0.25 s. After a variable delay period of 1.4–7.5 s, the fixation ring was replaced by placeholders at all six possible stimulus cue locations (go cue). Monkeys were required to make a saccade within 0.5 s to the placeholder where the original stimulus cue was presented and maintain their gaze for 0.25 s for monkey T and either 0.25 s or 0.6 s for monkey K (these two gaze maintenance times were switched in different blocks for monkey K) to receive a juice reward. The monkeys were extensively trained, with close to perfect performance (monkey T, 96.1%; monkey K, 96.3%, mean across sessions). Fixation break errors were excluded from the calculation of percent correct rate.

S2.1.3 Recordings

After training was completed, we conducted an aseptic surgery under general anesthesia. We stereotypically implanted a plastic recording chamber on the lateral surface of the prefrontal cortex, under the guidance of structural MRI images (Supplementary Fig. 9). In monkey T, we implanted a cylindrical chamber (RC-T-S-P, internal diameter 12.7 mm, Gray Matter Research, MT.) in the right hemisphere (AP = 33, ML = 14.5; AP, anterior-posterior; ML, medio-lateral). A 32-channel semi-chronic microdrive system (SC-32, Gray Matter Research) was mounted inside this chamber. In monkey K, we implanted a cuboid chamber (width 12 mm, depth 16 mm, height, 15 mm, S-company ltd., Tokyo, Japan) over the principal sulcus in the left hemisphere.

We collected neural data in a total of 48 daily sessions (21 in monkey T; 27 in monkey K). In monkey T, we used the 32-ch microdrive (SC-32) that housed 32 single-contact tungsten electrodes with inter-electrode spacing of 1.5 mm. In monkey K, we used a 32-ch linear microelectrode array (Plexon U-Probe, Plexon, TX.) with an interelectrode spacing of 150 μm along a single shaft. We positioned the U-Probe by using a custom-made grid (width 12 mm, depth 16 mm, height, 10 mm) which had a total of 165 holes with 1 mm spacing. We advanced the U-Probe by a custom-made hydraulic microdrive (S-company ltd.).

Raw extracellular neural signals were amplified and recorded in reference to a titanium bone screw at the vertex (in monkey T) or the shaft of the linear array (monkey K) using a neural signal amplifier RZ2 Bioamp Processor (Tucker-Davis Technologies, FL.). Behavioral data (task-event information and eye-movement information) were also sent to the RZ2 Bioamp. Neural data acquisition was performed at a sampling frequency of 24414.08 Hz, and behavioral data acquisition at 1017.25 Hz. For analysis of spiking activity, the raw neural signal was filtered (300 Hz to 6 kHz) for offline sorting (Offline Sorter, Plexon). In monkey T, approximately three hours before each recording session, we took the monkey to

the testing room and advanced each electrode in the SC-32 by a minimum of 62.5 μm in order to ensure recording of new neurons. We then put the monkey back in the home cage until we brought it out again for the recording session. In monkey K, we adopted the method of the U-Probe insertion reported in (14). We first punctuated the dura using a guide tube (a shortened 23 gauge needle), and inserted the U-Probe array slowly, usually with a step of 500 μm. We kept monitoring electrocardiogram (pulsatory fluctuation) on superficial electrodes to identify the point of cortical entry. We usually left 3–5 superficial channels outside the cortex. After array insertion, we waited 1–1.5 hours until the recorded single-unit and multiunit activities indicated that the electrode array was stably positioned in the cortex. While waiting, the monkey watched nature and animal video clips and received a small snack on a monkey chair.

In monkey T only, to determine location of the frontal eye field (FEF), and confirm that our recording area was outside it, intracortical microstimulations (22 biphasic pulses, 0.2 ms duration at 333 Hz, $\leq$ 150 μA) were applied through microelectrodes. When eye movements were elicited below 50 μA, the site was considered to be in the low-threshold FEF. In monkey T, our recording area did not include the low-threshold FEF.

S2.1.4 Pre-processing

We excluded neurons that were recorded in fewer than 10 trials for any cue condition. For each monkey, we pooled neurons from all recording sessions to create pseudopopulations of 438 neurons for Monkey K (after we removed 1 neuron from monkey K's dataset due to an insufficient number of trials) and 625 neurons for Monkey T (no neurons were removed from monkey T's dataset). To compute neural firing rates, we convolved spike trains with a Gaussian kernel with a standard deviation of 25 ms. Trial-averaged trajectories of time-varying mean firing rates were computed separately for each neuron and each cue condition. For analysis methods that used cross-validation (see below), we split trials into separate train and test sets with a 1:1 train:test ratio, and computed trial-averaged trajectories for each training and test set (using 1:1 splits). For non-cross validated analyses, we either computed trial averages based on all the data, or on a subset of the data (see below). We aligned neural activity to either stimulus or go cue onset (see also below in Methods S2.7) and shifted activity by -50 ms to allow for the delay in time for information about these cues to enter PFC. For consistency with our simulations (see below), we subsampled neural firing rates at a 1-ms time resolution.

S2.2 Neural network models: overview

All our simulated networks (Figs. 2–4 and 6 and Supplementary Figs. 2–5, 7 and 11–13) evolved according to a canonical model of stochastic recurrent neural circuit dynamics (15, 16):

$$\tau \frac{\mathrm{d}\mathbf{x}^{(c)}(t)}{\mathrm{d}t} = -\mathbf{x}^{(c)}(t) + \mathbf{W}\,\mathbf{r}^{(c)}(t) + m_{\mathrm{h}}(t)\,\mathbf{h}^{(c)} + m_{\mathrm{g}}(t)\,\mathbf{g} + \mathbf{b} + \sigma\,\boldsymbol{\eta}^{(c)}(t) \tag{S1}$$

$$\text{with} \quad \mathbf{r}^{(c)}(t) = \mathbf{f}\left(\mathbf{x}^{(c)}(t)\right) \tag{S2}$$

where $\mathbf{x}^{(c)}(t) = (x_1^{(c)}(t), \ldots, x_N^{(c)}(t))^\top$ corresponds to the vector of (unitless) trial-averaged raw somatic membrane potentials of the N neurons of the network (15) in cue condition $c = 1, \ldots, C$ (initialised at $\mathbf{x}^{(c)}(t_0)$ at the beginning of the simulation t_0, which could be at or before stimulus onset at $t = 0$). $\mathbf{r}^{(c)}(t)$ is their momentary firing rates, with $\mathbf{f}(\mathbf{x})$ being the activation function that converts membrane potentials to firing rates, τ is the effective time constant of the cell), $\mathbf{W}$ is the recurrent weight matrix (shown e.g. in Fig. 2a and g), $\mathbf{h}^{(c)}$ is the input given to the network depending on the stimulus cue, $\mathbf{g}$ is the stimulus-cue-independent go cue that occurs at the go time t_{go}, $m_{\mathrm{h}}(t)$ and $m_{\mathrm{g}}(t)$ are box car 'masking' kernels such that the stimulus and go cues are only effective within a limited period at the beginning and end of the trial, respectively, $\mathbf{b}$ is a cue-independent bias, σ is the standard deviation of the noise process, and $\boldsymbol{\eta}^{(c)}(t)$ is a sample from a standard (mean 0 and variance 1) Gaussian white (temporally and spatially) noise process.

Networks shown in different figures corresponded to different special cases of Eqs. S1 and S2 (see Table S1). Specifically, for linear networks $\mathbf{f}(\mathbf{x}) = \mathbf{x}$ was the identity function. For nonlinear networks $f_i(\mathbf{x}) = [x_i]_+$ was the rectified linear (ReLU) activation function applied element-wise (except for the ring attractor networks where we used $f_i(\mathbf{x}) = \tanh(x_i)$; Supplementary Fig. 3). Given that the focus of our study was optimal information loading, stimulus inputs were either optimized numerically (Fig. 2, Fig. 6, Supplementary Figs. 2 and 3, Supplementary Fig. 5c,d, and Supplementary Figs. 11–13), or set to analytically computed values as dictated by our mathematical analysis (Figs. 3 and 4, Supplementary Fig. 4c,d, and Supplementary Fig. 5a,b), or as a baseline, set to random values (Figs. 3 and 4, Supplementary Fig. 4a,b, and Supplementary Fig. 5a,b). For networks used to study the effects of instantaneous initial conditions (Figs. 2 and 3 and Supplementary Figs. 2–5 and 7), the stimulus masking kernel was zero and instead the initial condition was set to the stimulus input; for other networks (Fig. 4, Fig. 5e–f, Fig. 6 and Supplementary Figs. 11–13) the stimulus masking kernel was a boxcar between 0 and 0.25 s. For task-optimized networks (Fig. 6 and Supplementary Figs. 11–13), the go cue masking kernel $m_{\mathrm{g}}(t)$ was a boxcar starting at go cue onset, t_{go} and lasting for 0.5 s, for all other networks it was set to 0 everywhere. The networks used to analyse the dynamics of information loading (Fig. 3, Supplementary Fig. 4, and Supplementary Fig. 5a,b) were deterministic by setting $\sigma = 0$, all other networks used noisy dynamics (see Table S1). We solved the dynamics of

Symbol	Fig. 2	Fig. 3a–c	Fig. 3d–e	Fig. 4	Units	Description
N	50	2	1000	100	-	number of neurons
t_0	0	0	0	-0.5	s	simulation start time
t_{go}	-	-	-	-	s	go cue time
$t_{\max}$	2	2	2	2.5	s	simulation end time
τ	0.05	0.05	0.05	0.2	s	effective time constant
$\mathbf{x}^{(c)}(t_0)$	$\mathbf{h}^{(c)}$	$\mathbf{h}^{(c)}$	$\mathbf{h}^{(c)}$	$\mathbf{0}$	-	initial condition
$\mathbf{f}(\cdot)$	nonlinear[a]	linear[a]	linear[a]	linear[a]	Hz	neural activation function
$\mathbf{W}$	optimized[b]	set[d]	$\overset{\text{iid.}}{\sim} \mathcal{N}\left(0, \frac{1}{N}\right)$[e]	$\overset{\text{iid.}}{\sim} \mathcal{N}\left(0, \frac{1}{N}\right)$[e]	s	weight matrix
C	6	1	1	2	-	number of stimuli
$\mathbf{h}^{(c)}$	optimized[c1]	set[f]	set[b]	set[b]	-	stimulus input
$\mathbf{g}$	-	-	-	-	-	go cue
$m_{\text{h}}(t)$	0	0	0	$K(0, 0.25)$[g]	-	stimulus masking kernel
$m_{\text{g}}(t)$	0	0	0	0	-	go cue masking kernel
$\mathbf{b}$	optimized[b]	$\mathbf{0}$	$\mathbf{0}$	$\mathbf{0}$	-	cue-independent bias
σ	0.05	0	0	variable[b]	-	noise standard deviation

Symbol	Fig. 5e–f	Fig. 6a–d and Supplementary Fig. 1e	Fig. 6e	Supplementary Fig. 2	Units	Description
N	20	50	20	50	-	number of neurons
t_0	0	-0.5	0	0	s	simulation start time
t_{go}	-	2	-	-	s	go cue time
$t_{\max}$	1	3	1	2	s	simulation end time
τ	0.05	0.05	0.05	0.05	s	effective time constant
$\mathbf{x}^{(c)}(t_0)$	$\mathbf{0}$	$\mathbf{0}$	$\mathbf{0}$	$\mathbf{h}^{(c)}$	-	initial condition
$\mathbf{f}(\cdot)$	linear[a]	nonlinear[a]	linear[a]	nonlinear[a]	Hz	neural activation function
$\mathbf{W}$	fit[b]	optimized[b]	fit[b]	optimized[b]	s	weight matrix
C	6	6	6	6	-	number of stimuli
$\mathbf{h}^{(c)}$	fit[b]	optimized[c2]	fit[b]	optimized[c1]	-	stimulus input
$\mathbf{g}$	-	$\sum_c \mathbf{h}^{(c)}$	-	-	-	go cue
$m_{\text{h}}(t)$	$K(0, 0.25)$[g]	$K(0, 0.25)$[g]	$K(0, 0.25)$[g]	0	-	stimulus masking kernel
$m_{\text{g}}(t)$	0	$K\left(t_{\text{go}}, t_{\text{go}} + 0.5\right)$[g]	0	0	-	go cue masking kernel
$\mathbf{b}$	fit[b]	optimized[b]	fit[b]	optimized[b]	-	cue-independent bias
σ	0	0.05	0	0	-	noise standard deviation

Symbol	Supplementary Fig. 3	Supplementary Fig. 7b	Supplementary Fig. 7c	Supplementary Fig. 7d	Units	Description
N	50	50	50	20	-	number of neurons
t_0	0	0	0	0	s	simulation start time
t_{go}	-	-	-	-	s	go cue time
$t_{\max}$	2	2	2	2	s	simulation end time
τ	0.05	0.05	0.05	0.05	s	effective time constant
$\mathbf{x}^{(c)}(t_0)$	$\mathbf{h}^{(c)}$	$\mathbf{h}^{(c)}$	$\mathbf{h}^{(c)}$	$\mathbf{h}^{(c)}$	-	initial condition
$\mathbf{f}(\cdot)$	nonlinear[a]	linear[a]	nonlinear[a]	linear[a]	Hz	neural activation function
$\mathbf{W}$	optimized[b]	optimized[b]	optimized[b]	fit[b]	s	weight matrix
C	36 (6)[h]	6	6	6	-	number of stimuli
$\mathbf{h}^{(c)}$	optimized[c2]	optimized[c1]	optimized[c1]	fit[b]	-	stimulus input
$\mathbf{g}$	-	-	-	-	-	go cue
$m_{\text{h}}(t)$	0	0	0	0	-	stimulus masking kernel
$m_{\text{g}}(t)$	0	0	0	0	-	go cue masking kernel
$\mathbf{b}$	optimized[b]	optimized[b]	optimized[b]	fit[b]	-	cue-independent bias
σ	0.05	0	0.05	0	-	noise standard deviation

Table S1 | Continued on text page.

Symbol	Supplementary Fig. 4a,b	Supplementary Fig. 4c,d	Supplementary Fig. 5a,b	Units	Description
N	100	100	10, 100, 1000	-	number of neurons
t_0	0	0	0	s	simulation start time
t_{go}	-	-	-	s	go cue time
t_{max}	2	2	2	s	simulation end time
τ	0.05	0.05	0.05	s	effective time constant
$\mathbf{x}^{(c)}(t_0)$	$\mathbf{h}^{(c)}$	$\mathbf{h}^{(c)}$	$\mathbf{h}^{(c)}$	-	initial condition
$\mathbf{f}(\cdot)$	linear^a	linear^a	linear^a	Hz	neural activation function
$\mathbf{W}$	$\overset{\text{iid.}}{\sim} \mathcal{N}\left(0, \frac{1}{N}\right)^e$	$\overset{\text{iid.}}{\sim} \mathcal{N}\left(0, \frac{1}{N}\right)^e$	$\overset{\text{iid.}}{\sim} \mathcal{N}\left(0, \frac{1}{N}\right)^e$	s	weight matrix
C	1	1	1	-	number of stimuli
$\mathbf{h}^{(c)}$	$\overset{\text{iid.}}{\sim} \mathcal{N}\left(0, \frac{1}{N}\right)$	optimized^{c_4}	set^b	-	stimulus input
$\mathbf{g}$	-	-	-	-	go cue
$m_h(t)$	0	0	0	-	stimulus masking kernel
$m_g(t)$	0	0	0	-	go cue masking kernel
$\mathbf{b}$	$\mathbf{0}$	$\mathbf{0}$	$\mathbf{0}$	-	cue-independent bias
σ	0	0	0	-	noise standard deviation

Symbol	Supplementary Fig. 5c,d	Supplementary Fig. 10f	Supplementary Fig. 10g	Supplementary Figs. 11–13	Units	Description
N	100	20	20	50	-	number of neurons
t_0	0	0	$t_{go} - 1$	-0.5	s	simulation start time
t_{go}	-	-	1	2	s	go cue time
t_{max}	2	1	1	3	s	simulation end time
τ	0.05	0.05	0.05	0.05	s	effective time constant
$\mathbf{x}^{(c)}(t_0)$	$\mathbf{h}^{(c)}$	$\mathbf{0}$	set^b	$\mathbf{0}$	-	initial condition
$\mathbf{f}(\cdot)$	linear^a	linear^a	linear^a	nonlinear^a	Hz	neural activation function
$\mathbf{W}$	$\overset{\text{iid.}}{\sim} \mathcal{N}\left(0, \frac{1}{N}\right)^e$	fit^b	fit^b	optimized^b	s	weight matrix
C	1	6	6	6	-	number of stimuli
$\mathbf{h}^{(c)}$	optimized^{c_3}	fit^b	-	optimized^{c_2}	-	stimulus input
$\mathbf{g}$	-	-	-	$\sum_c \mathbf{h}^{(c)}$	-	go cue
$m_h(t)$	0	$K(0, 0.25)^g$	0	$K(0, 0.25)^g$	-	stimulus masking kernel
$m_g(t)$	0	0	0	$K\left(t_{go}, t_{go} + 0.5\right)^g$	-	go cue masking kernel
$\mathbf{b}$	$\mathbf{0}$	fit^b	fit^b	optimized^b	-	cue-independent bias
σ	0.05	0	0	0.05	-	noise standard deviation

Table S1 | Parameters used in the simulations of our models.

^{a} For nonlinear networks, $f_i(\mathbf{x}) = [x_i]_+$ was the rectified linear (ReLU) activation function. For linear networks $f_i(\mathbf{x}) = x_i$. The only exception to this was when we created ring attractor networks (Supplementary Fig. 3) in which we used a tanh nonlinearity $f_i(\mathbf{x}) = \tanh(x_i)$. See also text.

^{b} See text for details.

^{c} Inputs were optimized either with both a norm constraint and an overall energy constraint (c_1); only an overall energy constraint (c_2); only a norm constraint (c_3); or so that the dynamics produced the mathematically minimal overall energy (c_4, see Section S5). See text for more details.

^{d} For the symmetric network, we used $\mathbf{W} = \begin{pmatrix} 0.375 & 0.625 \\ 0.625 & 0.375 \end{pmatrix}$; for the unconstrained network, we used $\mathbf{W} = \mathbf{R} \begin{pmatrix} 1 & 50 \\ 0 & -11.5 \end{pmatrix} \mathbf{R}^\top$, where $\mathbf{R}$ is the rotation matrix $\mathbf{R} = \begin{pmatrix} \cos(\theta) & -\sin(\theta) \\ \sin(\theta) & \cos(\theta) \end{pmatrix}$ with $\theta = 0.1$.

^{e} For the symmetric networks, we enforced $\mathbf{W} \leftarrow \frac{1}{2}(\mathbf{W} + \mathbf{W}^\top)$. For all networks we also shifted the obtained weight matrix by the identity matrix multiplied by a constant so that the largest real part in the eigenvalues of $\mathbf{W}$ is exactly 1 (i.e., the largest eigenvalue of the associated Jacobian would therefore be 0 due to the leak term), and we rejected any $\mathbf{W}$'s for which the top eigenvalue (the eigenvalue with largest real part) had an imaginary component. For Fig. 4, to provide a slightly better agreement between the model dynamics and the experimental recordings, we rejected any $\mathbf{W}$'s for which the inner product between the most amplifying mode and persistent mode was greater than 0.2 (i.e. we only kept $\mathbf{W}$'s that were relatively mathematically non-normal).

^{f} We used 3 possible input directions (which all had a Euclidean norm of 1): inputs either aligned with the most persistent mode ($\mathbf{x}^\mathrm{p}$), the most amplifying mode ($\mathbf{x}^\mathrm{a}$), or a random direction ($\mathbf{x}^\mathrm{r}$). For the symmetric model, $\mathbf{x}^\mathrm{p} = \mathbf{x}^\mathrm{a} = [0.707, 0.707]^\top$ and we used $\mathbf{x}^\mathrm{r} = [0.98, 0.18]^\top$. For the unconstrained model, $\mathbf{x}^\mathrm{p} = [0.995, 0.0998]^\top$, $\mathbf{x}^\mathrm{a} = [0.1537, 0.9881]^\top$ and we used $\mathbf{x}^\mathrm{r} = [0.8453, 0.5343]^\top$.

^{g} $K(t_1, t_2) = \begin{cases} 1 & \text{if } t_1 \leq t \leq t_2 \text{ s,} \\ 0 & \text{otherwise.} \end{cases}$ In the table, t_{go} refers to the timing of the go cue (see text).

^{h} For training, we used $C = 36$ cue conditions. For our subsequent analyses (Supplementary Fig. 3), we used $C = 6$ cue conditions to be consistent with the other models.

Eqs. S1 and S2 using a first-order Euler–Maruyama approximation between t_0 and the simulation end time, t_{max}, with a discretization time step of 1 ms.

For analysis methods that used cross-validation (see below), for each cue condition, we simulated network dynamics twice with independent realizations of $\boldsymbol{\eta}^{(c)}(t)$, to serve as train and test data. For other analyses, we used a single set of simulated trajectories. All analyses involving networks with randomly generated (or initialized) connectivities that also did not require re-fitting their responses with other networks (Fig. 2, Fig. 4, Fig. 6c,d, Supplementary Figs. 1–3, Supplementary Fig. 7a–c, and Supplementary Figs. 11–13) were repeated a total of n = 100 times, consisting of 10 different networks and 10 different simulations. For those analyses that did require the re-fitting of nonlinear networks' responses with linear deterministic networks (Supplementary Fig. 7d and Fig. 6e), we used one simulation of the original (stochastic nonlinear) network, so n = 10 simulations in total.

S2.3 Nonlinear networks

For the dynamical equations of nonlinear networks, see Methods S2.2. For nonlinear networks (Figs. 2 and 6, Supplementary Figs. 2 and 3, Supplementary Fig. 7c, and Supplementary Figs. 11–13), we ensured that they performed working memory maintenance competently by optimizing their free parameters, $\mathbf{W}$, $\mathbf{b}$, and $\mathbf{h}^{(c)}$ for appropriate cost functions (see below, Methods S2.3.3).

S2.3.1 Nonlinear networks with instantaneous inputs

Following classical theoretical approaches to attractor network dynamics, we first used nonlinear neural networks in which stimulus inputs acted instantaneously to determine the initial conditions of the dynamics Fig. 2 and Supplementary Figs. 2, 3 and 7. These networks were optimized using a 'just-in-time' cost function (Methods S2.3.3) under one or two constraints. First, for all these networks, we constrained stimulus inputs to have a Euclidean norm of 3 so that we could compare information loading strategies fairly when inputs were constrained to lie in certain subspaces (see also below): either the persistent subspace, persistent nullspace, locally persistent subspace, locally most amplifying subspace, or a random subspace (Fig. 2f,l, Supplementary Fig. 2, and Supplementary Fig. 7). . We also obtained qualitatively very similar results without this norm constraint, with only a more general energy-based penalty (16–19) (Fig. 6 and Supplementary Figs. 3 and 11–13, see also Methods S2.3.3). Second, for symmetric networks, we enforced $\mathbf{W} \leftarrow \frac{1}{2}(\mathbf{W} + \mathbf{W}^\top)$.

These networks were trained in two epochs. For the first 1000 training iterations, we optimized all free parameters. After this, we confirmed that our trained networks did indeed have attractors (i.e. that they were attractor networks) and determined where these attractors were in state space by finding the stable fixed points of the networks' dynamics (Fig. 2d,j, Supplementary Fig. 2b,f, and Supplementary Fig. 3a)—see below. We then continued for another 1000 training iterations (without any firing rate regularization, $\alpha^{(2)}_{\text{nonlin}} = 0$ in Eq. S3) with only optimizing the initial conditions, $\mathbf{h}^{(c)}$, while keeping the other parameters, $\mathbf{W}$ (Fig. 2a,g) and $\mathbf{b}$, fixed at the values obtained at the end of the first 1000 iterations. We did this so that we could fairly compare different initial conditions that are constrained to lie in different subspaces but which otherwise rely on the same underlying network dynamics. We considered three possible scenarios for introducing additional constraints on the initial conditions (beside the one on their norm, see above): they were either projected and then restricted to the persistent subspace or to the persistent nullspace (see Methods S2.7.1 for how these subspaces were computed), or there was no such constraint applied so that they could utilize any direction in the full state space of the network. In addition, to understand the link between the linearized (Methods S2.4.2) and original (above) forms of the dynamics of these networks, we also considered three more constraints on the initial conditions: constraining them to the most persistent, most amplifying, or a random subspace of the linearized dynamics (Supplementary Fig. 7a–c).

S2.3.2 Nonlinear networks with temporally extended inputs

To more closely follow the experimental paradigms which we modelled, we also used nonlinear networks in which stimuli provided temporally extended inputs (Fig. 6 and Supplementary Figs. 11–13). To construct these networks, stimulus inputs and the weight matrix were freely optimized (Methods S2.3.3), without any constraints, and optimization proceeded for a full 2000 iterations, without dividing training into different epochs.

S2.3.3 Cost functions and training for nonlinear networks

To investigate how different cost functions impact network dynamics (Fig. 6), we trained networks using one of four cost functions: a 'cue-delay' cost, a 'full-delay', a 'just-in-time' cost, and an 'after-go' cost. These costs only differed in terms of the time period in which we applied the cost function. The general form of the cost function we used was a cross entropy

loss plus a regularisation term:

$$\mathcal{L} = \left\langle -\alpha^{(1)}_{\text{nonlin}} \sum_{c=1}^{6} \left(\mathbf{y}^{(c)}\right)^{\top} \int_{T_1}^{T_2} \log\left(\text{Softmax}\left(\mathbf{W}_{\text{out}}\, \mathbf{r}^{(c)}(t) + \mathbf{b}_{\text{out}}\right)\right) \mathrm{d}t + \alpha^{(2)}_{\text{nonlin}} \sum_{c=1}^{6} \int_{t_0}^{t_{\max}} \left\| \mathbf{r}^{(c)}(t) \right\|_2^2 \mathrm{d}t \right\rangle \tag{S3}$$

where T_1 and T_2 determine the time period in which we applied the cost, $\alpha^{(1)}_{\text{nonlin}}$ and $\alpha^{(2)}_{\text{nonlin}}$ control the relative contributions of the cross-entropy loss and firing rate regularisation, $\mathbf{W}_{\text{out}} \in \mathcal{R}^{6\times N}$ and $\mathbf{b}_{\text{out}} \in \mathcal{R}^{6}$ include the 6 sets of 'readout' weights and biases, respectively, and $\mathbf{y}^{(c)} \in \mathcal{R}^{6}$ is a one-hot vector where $y^{(c)}_i = \begin{cases} 1 & \text{if } i = c \\ 0 & \text{otherwise} \end{cases}$ defining the 'target' output for each cue condition. We initialized elements of the network parameters $\mathbf{W}$, $\mathbf{b}$, $\mathbf{h}^{(c)}$, as well as the readout parameters $\mathbf{W}_{\text{out}}$ and $\mathbf{b}_{\text{out}}$ from a Gaussian distribution with mean 0 and variance $1/N$, and then optimized using gradient descent with Adam optimization (20), where gradients were obtained from back-propagation through time. The angle brackets, $\langle\cdot\rangle$, denote averaging over batch sizes of 50 random realisations of $\mathbf{r}^{(c)}$. We used a learning rate of 0.0005.

See Table S2 for how we set the parameters of Eq. S3 (T_1, T_2, t_0, $\alpha^{(1)}_{\text{nonlin}}$ and $\alpha^{(2)}_{\text{nonlin}}$) depending on the cost function and the level of regularization. Briefly, the cue-delay cost included both the cue (between stimulus cue onset and offset) and the delay period (between stimulus cue offset and go cue onset), the full-delay cost the included delay period but not the cue period, the just-in-time cost started between stimulus onset and the earliest go time and ended at the onset of the go cue, and the after-go cost started at go cue onset and lasted for the duration of the go cue (0.5 s). For simulating the random delay task (Fig. 6 and Supplementary Figs. 11–13), analogous to what animals need to solve (see below), we sampled the go time uniformly between $t_{\text{go}} = 0.75$ s and $t_{\text{go}} = 2$ s. For just-in-time (Fig. 2 and Supplementary Fig. 2) and after-go trained networks (Supplementary Fig. 12), we also used a fixed delay task with a simulation end time of $t_{\max} = 2$ s or a go time of $t_{\text{go}} = 2$ s, respectively. For the other cost functions, networks trained on the fixed delay task yielded very similar dynamics to their counterparts trained on the variable delay task. We set $\alpha^{(1)}_{\text{nonlin}}$ and $\alpha^{(2)}_{\text{nonlin}}$ so that networks could reliably learn the task (at performance levels comparable across different settings) while also exhibiting relatively stable dynamics (i.e. if $\alpha^{(1)}_{\text{nonlin}}/\alpha^{(2)}_{\text{nonlin}}$ is too large, the network dynamics can explode whereas if $\alpha^{(1)}_{\text{nonlin}}/\alpha^{(2)}_{\text{nonlin}}$ is too small, the network is not able to learn the task). Note that vanishing gradients during training also impacted the value of $\alpha^{(1)}_{\text{nonlin}}$ that was required for different networks to exhibit similar performance (Fig. 6c). Nevertheless, $\alpha^{(2)}_{\text{nonlin}}$ was varied by an order of magnitude between Fig. 6 and Supplementary Figs. 11–13 to specifically test the robustness of our results to this parameter.

S2.3.4 Optimized ring attractor networks

When training to create ring attractor networks (Supplementary Fig. 3), we made three modifications to the nonlinear networks described above. First, in line with other approaches for optimizing recurrent neural networks (19, 21–23), we used a hyperbolic tangent nonlinearity because the saturation of this nonlinearity greatly encouraged a continuous attractor to form compared with a ReLu nonlinearity. Second, we trained networks with 36 cue conditions, and then subsequently restricted our analyses to 6 evenly spaced cue conditions to keep consistency with our other analyses. Third, we used a cost function that measured estimation (or fine, rather than coarse, discrimination) performance across those 36 conditions, thus encouraging a ring attractor to form:

$$\mathcal{L} = \left\langle \alpha^{(1)}_{\text{nonlin}} \sum_{c=1}^{36} \int_{T_1}^{T_2} \left[1 - \cos\left(\hat{\theta} - \theta^{(c)}\right)\right] \mathrm{d}t + \alpha^{(2)}_{\text{nonlin}} \sum_{c=1}^{36} \int_{t_0}^{t_{\max}} \left\| \mathbf{r}^{(c)}(t) \right\|_2^2 \mathrm{d}t \right\rangle \tag{S4}$$

$$\text{with } \hat{\theta} = \text{atan2}\left(\mathbf{W}^{\text{y}}_{\text{out}}\, \mathbf{r}^{(c)}(t), \mathbf{W}^{\text{x}}_{\text{out}}\, \mathbf{r}^{(c)}(t)\right) \tag{S5}$$

where $\hat{\theta}$ is the population vector-decoded stimulus angle, such that $\text{atan2}(y, x)$ gives the angle that the vector $[x, y]$ makes with the x-axis, $\mathbf{W}^{\text{x}}_{\text{out}}, \mathbf{W}^{\text{y}}_{\text{out}} \in \mathcal{R}^{1\times N}$ are 2 sets of 'readout' weights defining the plane in which decoded angles are defined, and $\theta^{(c)} = 2\pi \frac{c}{36}$ is the target angle for cue condition c. All other terms were the same as those defined in Methods S2.3.3. See also Section S8 for a derivation showing how the cost function Eq. S4 relates to Fisher information.

S2.4 Linear networks

For the dynamical equations of linear networks, see Methods S2.2. Linear networks were either constructed '*de novo*' (Figs. 3 and 4 and Supplementary Figs. 4 and 5), obtained by a local linearization of canonical nonlinear dynamical systems (Supplementary Fig. 6) or of nonlinear neural network dynamics (Supplementary Fig. 3e and Supplementary Fig. 7a–c), or they were fitted to neural responses obtained from experiments (Fig. 5f, and Supplementary Fig. 10f,g) or the simulation of nonlinear networks (Fig. 6e and Supplementary Fig. 7d).

Cost function	T_1 (s)	T_2 (s)	$\alpha^{(1)}_{\text{nonlin}}$ (1/s)	$\alpha^{(2)}_{\text{nonlin}}$ (1)	Figures
cue-delay	0	t_{go}	20	0.00005	Fig. 6 and Supplementary Fig. 11
				0.0005	Supplementary Fig. 11
full-delay	0.25	t_{go}	20	0.00005	Supplementary Fig. 13
				0.0005	Supplementary Fig. 13
just-in-time	0.5	$t_{\max}$	33	0.00005	Fig. 2 and Supplementary Fig. 2
	0.75	t_{go}	33	0.00005	Fig. 6 and Supplementary Fig. 11 and Supplementary Fig. 1e
				0.0005	Supplementary Fig. 11
	0.5	$t_{\max}$	33	0.05	Supplementary Fig. 3
after-go	t_{go}	$t_{\text{go}} + 0.5$	10	0.00005	Fig. 6
				0.0005	Supplementary Fig. 12

Table S2 | Parameters for nonlinear network optimization. Times T_1 and T_2 are relative to stimulus onset at $t = 0$. Units are shown in parentheses after the name of the corresponding parameter.

S2.4.1 De novo linear networks

We used *de novo* linear networks to develop an analytical understanding of the dynamics of optimal information loading. These networks included small 2-neuron networks with hand-picked parameters (see Table S1) chosen to illustrate the differences between normal (symmetric) and non-normal (unconstrained) dynamics and the effects of different initial conditions (Fig. 3a–c), as well as large networks (with 10, 100, or 1000 neurons) with randomly generated parameters (Fig. 3d,e, Fig. 4, Supplementary Fig. 4, and Supplementary Fig. 5a,b; see Table S1). We always set the largest eigenvalue of the weight matrix to be exactly 1 (thus setting the largest eigenvalue of the associated Jacobian to 0 due to the leak term) so that these networks had an integrating or 'persistent' mode (4, 24–26) (see Table S1).

Initial conditions (Fig. 3, Supplementary Fig. 4, and Supplementary Fig. 5a,b) or temporally extended inputs (Fig. 4) were determined by computing the most persistent and amplifying direction(s) based on the Jacobian of the dynamics (Figs. 3 and 4, and Supplementary Fig. 5a,b, see Methods S2.7.1; for how initial conditions were determined in Supplementary Fig. 4c,d see Section S5.8). For the networks in Fig. 4, we also added a small amount of noise to the input to allow for some transient dynamics for all input directions (see Fig. 4c at 0 s). Alternatively, we optimized initial conditions for maximal asymptotic overlap with the most persistent mode (Supplementary Fig. 5c,d; see below). For setting the noise level, σ, in these networks, we considered two scenarios: noise matched (Fig. 4a, light green and gray) and performance matched (Fig. 4a, dark green and black). For noise matched simulations, we first determined the highest value of σ that still allowed us to obtain 100% decodability (using a delay-trained decoder) for all networks when receiving inputs aligned with the most amplifying mode (Fig. 4a, red). This resulted in $\sigma = 0.1$ for symmetric models, and $\sigma = 0.17$ for unconstrained models. We then used the same σ for simulations using inputs aligned with the most persistent and random directions. For performance matched simulations, we used a different value of σ for each possible input direction so that all models achieved 100% decodability using a delay-trained decoder. For symmetric models, this required $\sigma = 0.1$ for inputs aligned with either the persistent or most amplifying modes, and $\sigma = 0.005$ for random inputs. For unconstrained models, this required $\sigma = 0.17$ for inputs aligned with the most amplifying mode, $\sigma = 0.02$ for inputs aligned with the persistent mode, and $\sigma = 0.005$ for random inputs. (Note that, consistent with our theory, smaller noise levels were necessary to achieve the same desired level of performance for input directions that were predicted to be increasingly suboptimal by our analysis.)

To demonstrate that the initial conditions along the most amplifying directions, obtained by control theoretic analyses, were indeed optimal for maximising the overlap with the most persistent mode (the measure of optimality we used for these networks, Fig. 3c,e), we also used a direct numerical optimization approach, analogous to that used to optimize initial conditions in our nonlinear networks (Figs. 2 and 6, see also Methods S2.3.3). Specifically, we optimized $\mathbf{h}^{(c)}$ (constrained to have unit Euclidean norm) with gradient descent using Adam optimization (20) with gradients obtained from back-propagation through time using the following cost function

$$\mathcal{L} = \int_{1.5\text{ s}}^{2\text{ s}} \left[\tanh\left(\mathbf{v}_1^\top \mathbf{x}(t)\right) - 1\right]^2 \mathrm{d}t \qquad \text{(S6)}$$

where $\mathbf{v}_1$ is the eigenvector associated with eigenvalue 0 of the Jacobian (i.e. the most persistent mode). We used a learning rate of 0.0001. We performed the above training procedure independently for 100 random noisy networks (either symmetric or unconstrained) and we show averaged results in Supplementary Fig. 5c,d. We also used random initial conditions as controls. These had elements that were either sampled from a standard normal distribution (re-scaled to have unit Euclidean norm) in large networks (Fig. 3d,e, Fig. 4, and Supplementary Fig. 5a,b), or in the case of 2-neuron networks, quasi-randomly chosen (with unit Euclidean norm) for illustrative purposes (Fig. 3a–c).

S2.4.2 Local linearization of nonlinear dynamics

To better understand how the dynamics of optimal information loading that we identified in linear networks apply to non-linear attractor dynamics, we performed a local linearization of our simulated nonlinear networks (Supplementary Fig. 3e, Supplementary Fig. 6, and Supplementary Fig. 7a–c). This approach required access to the 'true' dynamical equations of the nonlinear networks—which we had by construction.

We performed local linearizations of the original nonlinear network dynamics in $\mathbf{x}$-space (the space of variables in which the dynamics was defined, Eq. S1) around the origin (we found empirically that initial conditions were distributed close to the origin)—which served as the reference point with respect to which the norm of optimized initial conditions was constrained in the networks we linearized (Methods S2.3; analogous to our analysis of information loading in linear networks, Fig. 3a–e, and see also Methods S2.7.2). As the ReLU firing rate nonlinearity of these networks is non-differentiable at exactly the origin, we computed the 'average' Jacobian of the system in the immediate vicinity of the origin instead (this allowed us to use the same linearization and the same set of amplifying modes for all initial conditions; we obtained similar results by linearizing separately for each initial condition). Because the derivative of each ReLU is 0 or 1 in half of the activity space of the network, this resulted in the Jacobian $\mathbf{J} = \frac{1}{2}\mathbf{W}^* - \mathbf{I}$, where $\mathbf{W}^*$ is the weight matrix of the original nonlinear network. Note that one obtains the same result even without averaging, by regarding the ReLu nonlinearity as the limiting case of the soft-ReLu nonlinearity: $[x]_+ = \lim_{\beta\to\infty} \frac{1}{\beta} \ln\left(1 + e^{\beta x}\right)$, of which the derivative at $x = 0$ is $\frac{1}{2}$ (at any value of the inverse temperature, β) and thus results in the same Jacobian as above. We confirmed that the resulting dynamics were always stable (largest real eigenvalue of $\mathbf{J}$ was less than 0). We then used this system to identify the locally (around the origin) most amplifying or most persistent modes (Supplementary Fig. 7a).

For simulating these linearized networks (Supplementary Fig. 7b), we then used the Jacobian we thus obtained to map the resulting linearized dynamics to a deterministic integrator with the effective weight matrix $\mathbf{W} = (\mathbf{J} + \mathbf{I}) - \lambda_{\max}\,\mathbf{I}$, where $\lambda_{\max}$ is the largest real eigenvalue of $\mathbf{J}$. Thus, the resulting dynamics were always marginally stable (largest real eigenvalue of $\mathbf{J}$ was exactly 0). (Note that for subsequent analyses involving most persistent and amplifying modes, we used the original weight matrix, see more in Methods S2.7.1. Nevertheless, the most persistent modes of the weight matrices we used for simulation and those we used in subsequent analyses were identical, as they only relied on the eigenvectors of the weight matrix, or the Jacobian, and the rank order of their associated eigenvalues, which this stabilization did not affect. We also checked numerically that making the system marginally stable only had very minor effects on the most amplifying modes, with correlations between the most amplifying modes of the original and simulated dynamics being above 0.9. Thus, in these respects, our simulations were representative of the dynamics of the original systems.) The bias parameters, $\mathbf{b}$, were the same as in the original nonlinear networks. The initial conditions, $\mathbf{h}^{(c)}$, were either the ones we originally optimized for the nonlinear dynamics without any constraints (beside a constraint on their norm), or they were optimized while constraining them to the most persistent, most amplifying, or a randomly chosen subspace of these linearized dynamics (all were of the same dimensionality for a fair comparison, Supplementary Fig. 7).

For ring attractor networks (Supplementary Fig. 3), which used a tanh nonlinearity (Methods S2.3.4), the associated linearized system around the origin was given by the Jacobian $\mathbf{J} = \mathbf{W} - \mathbf{I}$, which we then used to identify the locally most amplifying and persistent modes (Supplementary Fig. 3e).

We used the same approach to linearize the dynamics of the canonical minimal nonlinear attractor dynamics that we used to gain insights into information loading in nonlinear systems (Methods S2.6, see also Section S6 and Supplementary Fig. 6). In this case, the Jacobian was well defined at the origin, so there was no need to average it. For consistency with the notation and terminology we use in the rest of this paper, and without loss of generality (as linear dynamical systems and linear neural networks are isomorphic), we refer to the resulting linear dynamical system as a 'linear neural network' and define it by its 'effective' weight matrix (defined via the Jacobian as above). Initial conditions were magnitude-matched and chosen to align with the most persistent or the most amplifying direction extracted from the Jacobian (Methods S2.7.1), or chosen randomly, or varied systematically to cover the whole range of possible directions. There were no other parameters for these linearized 'networks'.

S2.4.3 Fitting linear neural networks to neural responses

In order to be able to apply our theoretically derived measures of optimal information loading without having access to the true dynamics of the system, we also created linear neural networks whose parameters were fitted to experimental data (see below). As a control, we repeated the same fitting procedure with simulated nonlinear networks to validate that our approach provides meaningful results when 1. we do not have access to the true dynamics but only to samples of activities generated by those dynamics, and 2. we also cannot assume that the true dynamics are linear.

We fitted deterministic linear neural networks to 1 s of trial-averaged neural activity (experimentally recorded, or simulated by a nonlinear neural network model). For the main analyses (Fig. 5d–f, Fig. 6e, and Supplementary Fig. 7d), we used data starting from the onset of the stimulus cue. For the control analysis of late delay experimental recordings (Supplementary Fig. 10g), we used the final 1 s of neural activity just prior to the go cue. For the shuffle control (Fig. 5d; dark gray, and

Supplementary Fig. 10f), we again used data starting from stimulus onset but randomly shuffled neural activity across time and proceeded by fitting this shuffled data instead.

For fitting high dimensional neural data, we first performed principal components analysis on neural activity (dimensions: neurons, data points: time points, indexed by t, and cue conditions, indexed by c), and projected it through the principal components (PCs): $\mathbf{x}_*^{(c)}(t) = \mathbf{P}\,\mathbf{r}_*^{(c)}(t)$, where the columns of $\mathbf{P}$ are top 20 principal components of the data, and $\mathbf{r}_*^{(c)}(t)$ is trial averaged neural responses (mean-centered, see above) at time t in condition c. These top 20 PCs captured approximately 75% and 76% of variance for monkeys K and T, respectively during the cue and early delay period (Fig. 5d–f), 70% and 60% of variance for monkeys K and T, respectively during the late delay period (Supplementary Fig. 10g), and over 95% of the variance for all simulated neural activities (Fig. 6e). The projected neural activity time courses of the neural data, $\mathbf{x}_*^{(c)}(t)$, served as the targets that needed to be matched (after a suitable linear transformation with 'read-out' matrix $\mathbf{C} \in \mathcal{R}^{20\times 20}$) by the neural activity time courses generated by the fitted neural network's dynamics in the corresponding cue conditions, $\mathbf{x}^{(c)}(t)$ (Eqs. S1 and S2). For fitting the parameters of the network ($\mathbf{W}$, $\mathbf{h}^{(c)}$, $\mathbf{b}$) and the readout matrix ($\mathbf{C}$), we used the following cost function:

$$\mathcal{L} = \varepsilon^2 + \alpha_{\text{lin}} \left[\|\mathbf{C}\|_\text{F}^2 + \|\mathbf{b}\|_2^2 + \sum_{c=1}^{6} \left\|\mathbf{h}^{(c)}\right\|_2^2 \right] \tag{S7}$$

with $$\varepsilon^2 = \frac{1}{6}\sum_{c=1}^{6} \int_{0\text{ s}}^{1\text{ s}} \left(\mathbf{e}^{(c)}(t)\right)^\top \mathbf{D}\,\mathbf{e}^{(c)}(t)\;\mathrm{d}t \qquad \text{being the mean squared error of the fit} \tag{S8}$$

and $$\mathbf{e}^{(c)}(t) = \mathbf{C}\,\mathbf{x}^{(c)}(t) - \mathbf{x}_*^{(c)}(t) \qquad \text{the momentary error} \tag{S9}$$

where $\mathbf{D}$ is a diagonal matrix with the variances explained by the corresponding PCs in $\mathbf{P}$ on the diagonal (encouraging the optimization procedure to prioritize fitting the top PCs), $\|\cdot\|_\text{F}$ is the Frobenius norm of a matrix.

Also note that we had no constraints on $\mathbf{W}$ to define stable dynamics. Nevertheless, when fitting experimental recordings, and responses generated by nonlinear attractor networks, we found that the largest real eigenvalue of the fitted $\mathbf{W}$ was typically within the $0.95 \leq \lambda_\text{max} \leq 1.05$ range, i.e. the dynamics were near marginal stability, in line with the dynamics of our *de novo* linear neural networks (Methods S2.4.1), as well as of those that we obtained by local linearization (Methods S2.4.2). The only exception was when fitting the responses of nonlinear networks trained on an after-go-time cost (Methods S2.3.3) which resulted in dynamics without attractors and, consequently, the fitted linear dynamics typically had $\lambda_\text{max} > 1.05$.

We used Adam (20) to perform gradient descent optimization of $\mathbf{W}$, $\mathbf{h}^{(c)}$, $\mathbf{b}$, and $\mathbf{C}$ with gradients obtained from back-propagation through time, and a learning rate of 0.0001. We initialized elements of all of these parameters from a Gaussian distribution with mean 0 and variance 1/20 and we set the regularisation parameter to $\alpha_\text{lin} = 1/12$.

The stimulus-masking kernel ($m_\text{h}(t)$, Table S1) was matched to how the responses being fitted were obtained: with temporally extended or instantaneous inputs. Specifically, when fitting responses to temporally extended inputs (experimentally measured, Fig. 5f and Supplementary Fig. 10f, or simulated, Fig. 6e), the masking kernel of the fitted linear network matched the cue period. When fitting responses generated by networks driven by instantaneous inputs (Supplementary Fig. 7d), or when fitting the late delay period of experimental recordings (during which no stimulus is present, Supplementary Fig. 10g), the stimulus masking kernel was set to zero, and instead the initial condition of the fitted linear network was tuned to match the responses (see below).

In most cases (Fig. 5f, Fig. 6e, and Supplementary Fig. 10f), we set the initial condition $\mathbf{x}^{(c)}(t_0) = \mathbf{0}$. There were two exceptions to this. First, when fitting the late delay dynamics in the experimental recordings (Supplementary Fig. 10g), we set $\mathbf{x}^{(c)}(t_0) = \mathbf{C}^{-1}\,\mathbf{x}_*^{(c)}(t_0)$ (i.e. we fixed the initial condition of the latent dynamics to the data as no stimulus is present during the late delay period; we also observed qualitatively similar results when we included $\mathbf{x}^{(c)}(t_0)$ as a separate optimizable parameter in this case). Second, when fitting simulated data from models that used instantaneous stimulus inputs (Supplementary Fig. 7d), we set $\mathbf{x}^{(c)}(t_0) = \mathbf{h}^{(c)}$.

S2.5 Previous working memory models

We used the following dynamics for implementing all previous neural network models of working memory:

$$\mathbf{x}^{(c)} = \mathbf{W}\,\mathbf{r}^{(c)}(t) + m_\text{h}(t)\;\mathbf{h}^{(c)} + m_\text{g}(t)\;\mathbf{g} \tag{S10}$$

$$\tau_\text{r}\,\frac{\mathrm{d}\mathbf{r}^{(c)}(t)}{\mathrm{d}t} = -\mathbf{r}^{(c)}(t) + \mathbf{f}\left(\mathbf{x}^{(c)}(t)\right) + \mathbf{b}_\text{r} + \boldsymbol{\sigma}_\text{r}\,\boldsymbol{\eta}_\text{r}^{(c)}(t) \tag{S11}$$

where all symbols refer to the same (or a closely analogous, see below) quantity as in Eqs. S1 and S2. Note that we use this notation to best expose the similarities with and differences from the dynamics of our networks (Eqs. S1 and S2), rather than the original notation used for describing these models (1, 4, 6), but the dynamics are nevertheless identical to

those previously published. Overall, these dynamics are closely analogous to those that we used earlier for our networks with the following differences. First, for us, dynamics were defined in $\mathbf{x}$-space, with $\mathbf{r}$ being an instantaneous function of $\mathbf{x}$. Here, the dynamics are defined instead in $\mathbf{r}$-space (Supplementary Fig. 1a–d and Supplementary Fig. 8), with $\mathbf{x}$ being an instantaneous function of $\mathbf{r}$. (There are slightly different assumptions underlying these rate-based formulations of neural network dynamics when deriving them as approximations of the dynamics of spiking neural networks (27), and the two become identical in the case of linear dynamics.) As a result, time constants, $\boldsymbol{\tau}_\mathrm{r}$, biases, $\mathbf{b}_\mathrm{r}$, and the variance of noise, $\boldsymbol{\sigma}_\mathrm{r}$ (as well as noise itself, $\boldsymbol{\eta}_\mathrm{r}^{(c)}(t)$), are defined for $\mathbf{r}$ rather than $\mathbf{x}$. For nonlinear variants of these networks, there are also differences for the choice of single neuron nonlinearities, $\mathbf{f}(\cdot)$. Furthermore, some of these networks distinguish between excitatory and inhibitory cells, with different time constants, and noise standard deviations. Thus, each of these parameters is represented as a diagonal matrix, $\boldsymbol{\tau}_\mathrm{r}$ and $\boldsymbol{\sigma}_\mathrm{r}$, respectively, with each element on the diagonal storing one of two possible values of that parameter depending on the type (excitatory or inhibitory) of the corresponding neuron ($\tau_\mathrm{r}^\mathrm{E}$ and $\sigma_\mathrm{r}^\mathrm{E}$, or $\tau_\mathrm{r}^\mathrm{I}$ and $\sigma_\mathrm{r}^\mathrm{I}$, respectively). Most importantly, all of these networks used a set of parameters which were hand-crafted to produce the required type of dynamics, rather than optimized for a function (or to fit data) as in the case of our networks. In line with our analyses of experimental data and task-optimized networks (Figs. 5 and 6), simulations started at $t_0 = -0.5$ s, i.e. 0.5 s before stimulus cue onset (defined as $t = 0$), the stimulus cue lasted for 0.25 s, and the go cue appeared at $t_\mathrm{go} = 2$ s and lasted for 0.5 s. (Note that for these networks we considered the fixed-delay variant of the task as that is what these networks were originally constructed to solve.) As with our networks (Methods S2.2), we solved the dynamics of Eqs. S10 and S11 using a first-order Euler–Maruyama approximation between t_0 and the simulation end time with a discretization time step of 1 ms.

For analysis methods that used cross-validation (see below), we simulated network dynamics twice (for each cue condition) with independent realizations of $\boldsymbol{\eta}_\mathrm{r}^{(c)}(t)$, to serve as (trial-averaged) train and test data. For other analyses, we used a single set of simulated trajectories. All analyses involving these networks were repeated $n = 10$ times, using 10 different simulations (non-cross-validated) or simulation-pairs (cross-validated), each time with independent samples of $\boldsymbol{\eta}_\mathrm{r}^{(c)}(t)$.

Table S3 provides the values of most network and other parameters used for simulating each model. In the following we provide the additional details for each of these models that are not included in Table S3.

S2.5.1 Classical bump attractor model

The bump attractor model that we used (Supplementary Fig. 1a) has been described previously (see Ref. 1). The model contained separate excitatory and inhibitory populations. As in the discrete attractors model, the weight matrix was of the form

$$\mathbf{W} = \begin{pmatrix} \mathbf{W}^\mathrm{EE} & -\mathbf{W}^\mathrm{IE} \\ \mathbf{W}^\mathrm{EI} & -\mathbf{W}^\mathrm{II} \end{pmatrix} \tag{S12}$$

where the elements of $\mathbf{W}^\mathrm{IE}$,$\mathbf{W}^\mathrm{EI}$, and $\mathbf{W}^\mathrm{II}$ were set to $6.8/N$, $8/N$, and $1.7/N$, respectively. The excitatory sub-matrix $\mathbf{W}^\mathrm{EE}$ had a circulant form:

$$W_{ij}^\mathrm{EE} = \frac{6\, e^{1.5\cos\left(\frac{4\,\pi\,(i-j)}{N}\right)}}{\sum_{k=0}^{N/2-1} e^{1.5\cos\left(\frac{4\,\pi\,k}{N}\right)}} \tag{S13}$$

for cell-pairs $i, j = 1, \ldots, N/2$.

Stimulus cue inputs were also analogous to those used in the discrete attractors models and were set to

$$h_i^{(c)} = \frac{200\, e^{1.5\ \cos\left(\pi\left(\frac{4\,i}{N} - \frac{2\,c-1}{6}\right)\right)}}{\sum_{k=1}^{N/2} e^{1.5\ \cos\left(\pi\left(\frac{4\,k}{N} - \frac{2\,c-1}{6}\right)\right)}} \tag{S14}$$

for cues $c = 1, \ldots, 6$ and cells $i = 1, \ldots, N/2$ (i.e., as above, inputs were only delivered to the excitatory neurons).

S2.5.2 Discrete attractors model

The discrete attractors model that we used (Supplementary Fig. 1b) has been described previously (see the methods of Ref. 1). The model contained separate excitatory and inhibitory populations.

The weight matrix was of the form

$$\mathbf{W} = \begin{pmatrix} \mathbf{W}^\mathrm{EE} & -\mathbf{W}^\mathrm{IE} \\ \mathbf{W}^\mathrm{EI} & -\mathbf{W}^\mathrm{II} \end{pmatrix} \tag{S15}$$

Parameters used in network simulations of previous models						
Symbol	Supplementary Fig. 1a	Supplementary Fig. 1b	Supplementary Fig. 1c and Supplementary Fig. 8a,b,d,e	Supplementary Fig. 1d and Supplementary Fig. 8c,f	Units	Description
N	100	108	100	100	-	number of neurons
t_0	-0.5	-0.5	-0.5	-0.5	s	simulation start time
t_{go}	2	2	2	2	s	go cue time
t_{max}	3	3	3	3	s	simulation end time
τ	-	-	0.05	0.01	s	effective time constant
$\tau_{\text{r}}^{\text{E}}$	0.02	0.02	-	-	s	effective time constant (E neurons)
$\tau_{\text{r}}^{\text{I}}$	0.01	0.01	-	-	s	effective time constant (I neurons)
$\mathbf{r}^{(c)}(t_0)$	$\mathbf{0}$	$\mathbf{0}$	$\mathbf{0}$	$\mathbf{0}$	Hz	initial condition
$\mathbf{f}(\cdot)$	nonlinear[a]	nonlinear[a]	linear[a]	linear[a]	Hz	neural activation function
$\mathbf{W}$	set[b]	set[b]	set[b]	set[b]	s	weight matrix
C	6	6	6	6	-	number of stimuli
$\mathbf{h}^{(c)}$	set[b]	set[b]	set[b]	set[b]	-	stimulus input
$\mathbf{g}$	$\sum_c \mathbf{h}^{(c)}$	$\sum_c \mathbf{h}^{(c)}$	$\sum_c \mathbf{h}^{(c)}$	$\sum_c \mathbf{h}^{(c)}$	-	go cue
$m_{\text{h}}(t)$	$K(0, 0.25)^c$	$K(0, 0.25)^c$	$K(0, 0.25)^c$	$K(0, 0.25)^c$	-	stimulus masking kernel
$m_{\text{g}}(t)$	$K\left(t_{\text{go}}, t_{\text{go}} + 0.5\right)^c$	$K\left(t_{\text{go}}, t_{\text{go}} + 0.5\right)^c$	$K\left(t_{\text{go}}, t_{\text{go}} + 0.5\right)^c$	$K\left(t_{\text{go}}, t_{\text{go}} + 0.5\right)^c$	-	go cue masking kernel
$\mathbf{b}_{\text{r}}$	$b_{\text{r}}^{\text{E}} = 0.2$, $b_{\text{r}}^{\text{I}} = 0.5$	$b_{\text{r}}^{\text{E}} = -1.2$, $b_{\text{r}}^{\text{I}} = 0.28$	$\mathbf{0}$	$\mathbf{0}$	Hz	cue-independent bias
σ_{r}	-	-	0.02	0.02	Hz	noise standard deviation
$\sigma_{\text{r}}^{\text{E}}$	1	2	-	-	Hz	noise standard deviation (E neurons)
$\sigma_{\text{r}}^{\text{I}}$	3	1	-	-	Hz	noise standard deviation (I neurons)

Table S3 | Parameters used in previous models.

[a] For nonlinear networks, $f_i(\mathbf{x}) = \begin{cases} [x_i]_+^2 & \text{if } x_i \leq 1, \\ \sqrt{4x_i - 3} & \text{otherwise.} \end{cases}$. For linear networks $f_i(\mathbf{x}) = x_i$.

[b] See text for details.

[c] $K(t_1, t_2) = \begin{cases} 1 & \text{if } t_1 \leq t \leq t_2 \text{ s}, \\ 0 & \text{otherwise.} \end{cases}$

where the elements of $\mathbf{W}^{\mathrm{IE}}$,$\mathbf{W}^{\mathrm{EI}}$, and $\mathbf{W}^{\mathrm{II}}$ were set to $2.4/N$, $8/N$, and $2.6/N$, respectively. The excitatory sub-matrix $\mathbf{W}^{\mathrm{EE}}$ was constructed by dividing the population of excitatory cells into six clusters (of 9 neurons each), with each cluster corresponding to one of the stimulus cue conditions. Connections within each cluster were strong, with a value of $30/N$. Connections between neurons belonging to clusters that corresponded to adjacent stimulus cues were weaker, with a value of $2.5/N$. All other connections were very weak, with a value of $0.02/N$. This resulted in a block circulant structure for $\mathbf{W}^{\mathrm{EE}}$.

Stimulus cue inputs were set to

$$h_i^{(c)} \propto \frac{350\, e^{8\cos\left(\pi\left(\frac{4i}{N}-\frac{2c-1}{6}\right)\right)}}{\sum_{k=1}^{N/2} e^{8\cos\left(\pi\left(\frac{4k}{N}-\frac{2c-1}{6}\right)\right)}} \tag{S16}$$

for cues $c = 1,\ldots,6$ and cells $i = 1,\ldots,N/2$ (i.e. inputs were only delivered to the excitatory neurons).

S2.5.3 Linear integrator model

The linear integrator model that we used (Supplementary Fig. 1c and Supplementary Fig. 8a,d) has been described previously (see Ref. 4). There were no separate excitatory and inhibitory populations in this model, and the weight matrix was constructed such that network dynamics were non-normal, non-oscillatory, and stable with a single two-dimensional neutrally stable subspace (i.e. a plane attractor). We achieved this by defining $\mathbf{W}$ via its eigen-decomposition:

$$\mathbf{W} = \mathbf{V}\,\mathbf{D}\,\mathbf{V}^{-1} \tag{S17}$$

where the eigenvectors (columns of $\mathbf{V}$, denoted as $\mathbf{v}_j$, for $j = 1,\ldots,N$, with elements v_{ij}, for $i,j = 1,\ldots,N$) were generated by the following process:

1. Generating a random vector:

$$\nu_i \overset{\text{iid.}}{\sim} \mathcal{N}(0,1) \tag{S18}$$

for $i = 1,\ldots,N$.

2. Making the first 10% of vectors overlapping so that the resulting matrix is non-normal:

$$v_{ik} = \nu_i + \epsilon_{ik} \tag{S19}$$

$$\text{where } \epsilon_{ik} \overset{\text{iid.}}{\sim} \mathcal{N}\left(0, 0.05^2\right) \tag{S20}$$

for $i = 1,\ldots,N$ and $k = 1,\ldots,K$ with $K = 0.1\,N$.

3. Making the the other 90% of vectors orthogonal:

$$\mathbf{v}_{K+k} = \text{the } k\text{th column of Nullspace}(\mathbf{v}_1,\ldots\mathbf{v}_K) \tag{S21}$$

for $k = 1,\ldots,N-K$

4. Unit normalizing each vector:

$$\mathbf{v}_k \leftarrow \frac{\mathbf{v}_k}{\|\mathbf{v}_k\|_2^2} \tag{S22}$$

and the eigenvalues (λ_i, for $i = 1,\ldots,N$, the diagonal elements of the diagonal matrix $\mathbf{D}$) were generated by the following process

1. Generating random (real) values:

$$\lambda_i \overset{\text{iid.}}{\sim} \text{Uniform}(0, 0.8) \tag{S23}$$

for $i = 1,\ldots,N-2$.

2. Creating a pair of neutrally stable eigenmodes:

$$\lambda_{N-1} = \lambda_N = 1 \tag{S24}$$

The stimulus cue inputs were set to

$$\mathbf{h}^{(c)} = \mathbf{K} \begin{pmatrix} \cos\left(\frac{(c-1)\,\pi}{3}\right) \\ \sin\left(\frac{(c-1)\,\pi}{3}\right) \\ 1 \end{pmatrix} \tag{S25}$$

for cues $c = 1, \dots, 6$, and we considered two forms for $\mathbf{K}$: either $\mathbf{K} = \left[\mathbf{v}_{N-1}, \mathbf{v}_N, \mathbf{v}_{r_1}\right] + \left[\mathbf{v}_{r_2}, \mathbf{v}_{r_3}, \mathbf{v}_{r_4}\right]$ (Supplementary Fig. 1c and Supplementary Fig. 8a,d; as in the original formulation (4)) or $\mathbf{K} = \left[\mathbf{v}_{r_1}, \mathbf{v}_{r_2}, \mathbf{v}_{r_3}\right]$ (Supplementary Fig. 8b,e), where r_1, r_2, r_3, r_4 were randomly drawn integers over the range 1 to $N-2$. The first formulation of $\mathbf{K}$ ensured that stimulus cue inputs partially align with the persistent subspace, whereas the second formulation of $\mathbf{K}$ ensured that stimulus cue inputs align only with random directions.

S2.5.4 Feedforward network model

The linear feedforward network model that we used (Supplementary Fig. 1d and Supplementary Fig. 8c,f) has been described previously (see Refs. 5, 6). (For pedagogical purposes, we used the simplest set up consisting of a feedforward chain of neurons, see below. However, using a more general network model that contained 'hidden' feedforward chains (5) did not affect our analyses except for Supplementary Fig. 10e which, in contrast to the simple feedforward chain, could display overlap values greater than 0.5.) There were no separate excitatory and inhibitory populations in this model, and the weight matrix included a single chain running from neuron 1 to neuron N:

$$W_{ij} = \delta_{(i-1),j} \tag{S26}$$

for cell-pairs $i, j = 1, \dots, N$.

The stimulus cues provided random inputs delivered to only the first 10 neurons so that each input could pass through the feedforward network:

$$h_i^{(c)} \overset{\text{iid.}}{\sim} \mathcal{N}(0, 1) \tag{S27}$$

for cues $c = 1, \dots, 6$ and cells $i = 1, \dots, 10$.

S2.6 Canonical nonlinear systems with two stable fixed points

In order to illustrate the applicability of our analysis of optimal information loading in linear dynamical systems to the behaviour of nonlinear dynamical systems, we first studied two variants (either symmetric or non-symmetric) of a canonical nonlinear system that can exhibit two stable fixed points. (These systems are closely related to the damped, unforced Duffing oscillator which is a classic example of a [non-symmetric] system that can exhibit two stable fixed points. Additionally, the analysis of these systems also holds for the Duffing oscillator.)

The dynamics of the first system (which has a symmetric Jacobian matrix) are governed by

$$\begin{aligned} \tau_c \frac{\mathrm{d}x_1(t)}{\mathrm{d}t} &= x_1(t) - x_1^3(t) \\ \tau_c \frac{\mathrm{d}x_2(t)}{\mathrm{d}t} &= -x_2(t) \end{aligned} \tag{S28}$$

and the dynamics of the second system (which has a non-symmetric Jacobian matrix) are governed by:

$$\begin{aligned} \tau_c \frac{\mathrm{d}x_1(t)}{\mathrm{d}t} &= x_1(t) - x_1^3(t) + 3\,x_2(t) \\ \tau_c \frac{\mathrm{d}x_2(t)}{\mathrm{d}t} &= -x_2(t) \end{aligned} \tag{S29}$$

We used a cubic polynomial in Eqs. S28 and S29 because it is the lowest order polynomial that allows a system to exhibit 2 stable fixed points. Both systems exhibit 3 fixed points: both have a saddle point at the origin and both have 2 asymptotically stable fixed points at $(\pm 1, 0)$ (see Supplementary Fig. 6 for the state space dynamics of these two systems).

We solved the dynamics of Eqs. S28 and S29 using a first-order Euler approximation starting from $t = 0$ with a discretization time step of 0.02 and a time constant of $\tau_c = 0.4$ (note time was unitless for this model).

S2.7 Analysis methods

Here we describe methods that we used to analyse neural data. Whenever applicable, the same processing and analysis steps were applied to both experimentally recorded and model simulated data. As a first step in all our analyses, in line with previous work analysing neural population dynamics (28), we removed the stimulus cue-independent time-varying mean activity from each neuron's firing rate time series (see Fig. 5a for an example). (This was done separately for training and test data for cross-validated analyses, see below.) In most of our analyses, neural activities were aligned to stimulus cue onset defined to be at $t = 0$. However, due to the variable delay duration of the task (Fig. 1a), experimentally recorded neural activities were also aligned to go cue onset for analyses that required incorporating the late delay and go epochs (i.e. beyond the first 1.65 s after the stimulus cue onset; Fig. 5b–c, Supplementary Fig. 10a–c,g). For simulated neural activities, this was not necessary, as we always simulated our networks in a fixed-delay task for ease of analysis, even if they were optimized for a variable-delay task in accordance with how our experimental monkey subjects were trained.

S2.7.1 *Identifying amplifying, persistent, and other subspaces in network dynamics*

In order to understand the dynamics of neural networks with potentially complex and high-dimensional dynamics, and the way these dynamics depend on initial conditions, we identified specific subspaces within the full state space of these networks that were of particular relevance for our analyses. These subspaces served dual roles. First, as 'intervention tools', to ascertain their causal roles in high dimensional network dynamics, we used them to constrain the initial conditions of the dynamics of our networks (see also Methods S2.7.2). Second, as 'measurement tools', to reveal key aspects of the high-dimensional dynamics of neural networks, we used them to project high-dimensional neural trajectories into these lower dimensional subspaces (see also Methods S2.7.3).

Our main analyses relied on identifying the most persistent and most amplifying modes of a network. This required dynamics that were linear—either by construction, or by (locally) linearizing or linearly fitting dynamics that were originally nonlinear (see Table S1). We computed the most persistent mode(s) in one of two different ways. First, for networks that were either guaranteed to have stable dynamics by construction (i.e. those constructed *de novo*; Figs. 3 and 4 and Supplementary Figs. 4, 5 and 8), or were confirmed to be always stable in practice (i.e. those constructed by local linearization; Supplementary Fig. 3e, Supplementary Fig. 6, and Supplementary Fig. 7), we simply used the eigenvector(s) of the weight matrix $\mathbf{W}$ associated with the eigenvalue(s) that had the largest real part(s). Second, for networks that were fitted to nonlinear dynamics or recorded data, and whose dynamics could thus not be guaranteed to be stable (Fig. 5f, Fig. 6e, Supplementary Fig. 7d, and Supplementary Fig. 10f–h), we used the eigenvectors of $\mathbf{W}$ associated with the largest real eigenvalues that were less than or equal to $1 + \delta$ (with $\delta = 0.05$) (i.e. we find the slowest, or most persistent, modes of the network—the δ was mostly relevant only for the after-go-time networks of Fig. 6 and Supplementary Fig. 12 which exhibited eigenvalues substantially greater than 1 and setting δ less than 0.05 did not substantially change our results). (Note that an eigenvalue of 1 for $\mathbf{W}$ corresponds to an eigenvalue of 0 for the associated Jacobian of the dynamics due to the leak term.)

For computing the most amplifying modes, we performed an eigen-decomposition of the associated Observability Gramian $\mathbf{Q}$ (29, 30). Specifically, we obtained $\mathbf{Q}$ by solving the following Lyapunov equation:

$$\left(\tilde{\mathbf{W}} - \mathbf{I}\right)^{\top} \mathbf{Q} + \mathbf{Q}\left(\tilde{\mathbf{W}} - \mathbf{I}\right) + \mathbf{C}^{\top}\mathbf{C} = \mathbf{0} \tag{S30}$$

where $\tilde{\mathbf{W}}$ is the 'stabilized' weight matrix of the dynamics (and the $-\mathbf{I}$ terms represent the effect of the leak on the Jacobian of the dynamics, Eq. S1) and $\mathbf{C}$ is the read-out matrix of the network. The most amplifying mode(s) of the network are given as the eigenvector(s) of $\mathbf{Q}$ associated with the largest eigenvalue(s). Again, for networks that were guaranteed to have stable dynamics by construction (Figs. 3 and 4, Supplementary Fig. 7a–c, Supplementary Fig. 5, and Supplementary Fig. 8), $\tilde{\mathbf{W}} = \mathbf{W} - \epsilon\,\mathbf{I}$, where $\mathbf{W}$ is the original weight matrix of the dynamics and $\epsilon = 0.01$ (to ensure dynamical stability). For other networks, i.e. either linear networks fitted to experimental data (Fig. 5f and Supplementary Fig. 10f–h), linear networks fitted to simulated nonlinear dynamics (Fig. 6e and Supplementary Fig. 7d), or local linearizations of nonlinear dynamics (Supplementary Fig. 6, Supplementary Fig. 3e, and Supplementary Fig. 7a–c), we used $\tilde{\mathbf{W}} = \mathbf{W}$ unless the largest eigenvalue $\lambda_{\max}$ of $\mathbf{W}$ was greater than or equal 1, in which case we used $\tilde{\mathbf{W}} = \mathbf{W} - (\lambda_{\max} - 1 + \epsilon)\,\mathbf{I}$, to ensure that the linear dynamics with $\tilde{\mathbf{W}}$ were stable (which is required for calculating $\mathbf{Q}$). For networks obtained by fitting neural responses (experimentally recorded or simulated; Fig. 5f, Fig. 6e, Supplementary Fig. 7d, and Supplementary Fig. 10f–h), $\mathbf{C}$ was obtained by fitting those responses (Methods S2.4.3), as we wanted to understand how the fitted dynamics taking place in a latent space can generate the most discriminable fluctuations in (the principal components of) the neural responses to which they are related by this read-out matrix (although using $\mathbf{C} = \mathbf{I}$ did not change our results substantially). For all other networks (Figs. 3 and 4, Supplementary Fig. 7a–c, Supplementary Fig. 3e, Supplementary Fig. 5, and Supplementary Fig. 8), we simply used $\mathbf{C} = \mathbf{I}$, as the activity of these networks was supposed to be read out in the same space within which their dynamics took place.

We also applied methods which did not rely on the linearization (or linear fitting) of network dynamics. Our goal was to develop basic intuitions for how much the dynamics of the different simulated nonlinear networks of Fig. 2 and Supple-

mentary Fig. 2 used the persistent subspace of their dynamics. For this, we determined the 'persistent subspace' as the subspace spanned by the 5 principal components of the final 500 ms of neural activities ($\mathbf{x}$) across all 6 cue conditions, corresponding to 6 distinct attractors, and the 'persistent nullspace' of the network as the 45-dimensional subspace orthogonal to the persistent subspace. For plots showing the projection of network activities within the persistent subspace (Supplementary Fig. 2b,f and Supplementary Fig. 2c–d and g–h, bottom) we used the first two principal components of the full, five-dimensional persistent subspace of the network, as determined above. For plots showing the projection of network activities to persistent vs. cue-aligned directions (Fig. 2d,j, and Supplementary Fig. 2c–d and g–h, top right), 'persistent PC1' was determined as the direction spanning the two persistent states corresponding to the two cue conditions being illustrated (i.e. as above, spanning the final 500 ms of neural activities across the two cue conditions), and 'initial PC1 (orthogonalized)' was determined as the the direction spanning the two initial conditions corresponding to the two cue conditions being illustrated, orthogonalized with respect to the corresponding persistent PC1.

S2.7.2 *Subspace-constrained initial conditions*

When using the subspaces identified above as 'intervention tools', to constrain the initial conditions of our networks, we either used the single top most persistent or amplifying mode for linear networks with low-dimensional coding spaces (including the linearized canonical nonlinear attractor dynamical system; Figs. 3 and 4 and Supplementary Figs. 5 and 6), or numerically optimized initial conditions within the corresponding higher-dimensional subspaces (Fig. 2f,l, Supplementary Fig. 2c,d,g,h, Supplementary Fig. 7; see also Methods S2.3 and Methods S2.4). When the persistent subspace was extracted from neural responses (rather than from the dynamical equations of the network, Methods S2.7.1; Fig. 2f,l, Supplementary Fig. 2c,d,g,h, Supplementary Fig. 7a) we used different sets of simulations to generate data from which we could estimate the persistent subspace (as explained above), and to analyse network dynamics when initialized within these subspaces. In all cases, for a fair comparison, the magnitude of initial conditions was fixed (Methods S2.3.1, Methods S2.4.1), and only their direction was affected by constraining them to one of these subspaces.

S2.7.3 *Measures of subspace overlap*

In order to measure the overlap of high dimensional neural dynamics with the subspaces we identified, we used one of two methods. First, for analysing network dynamics across two conditions chosen to correspond to 'opposite' stimulus cues (Fig. 2d,j, Fig. 3c,d,e, Supplementary Fig. 2c,d,g,h, Supplementary Fig. 6c,d, and Supplementary Fig. 5), such that the coding part of the persistent subspace was one-dimensional, we simply measured the projection of neural dynamics onto the first eigenvector (i.e. the eigenvector associated with the largest real eigenvalue) of the corresponding subspace using a dot product:

$$\text{activity along mode}(t) = \mathbf{u}^\top \mathbf{x}(t) \tag{S31}$$

where $\mathbf{u}$ may correspond to the most persistent, or the most amplifying mode, or the first PC of the persistent-orthogonalized cue subspace (as defined above). We also used the same measure for visualising the quality of fit of linear neural network dynamics to experimental data (Methods S2.4.3) with $\mathbf{u}$ being the first PC of the full state space of neural firings rates (Fig. 5e). In those cases, when $\mathbf{u}$ had to be estimated from neural responses (Fig. 2d,j, Fig. 5e, Supplementary Fig. 2c,d,g,h), we used a cross-validated approach, using different subsets of the data to determine $\mathbf{u}$ and $\mathbf{x}(t)$ (from a single split of the data). In other cases, $\mathbf{u}$ was determined from the truly deterministic dynamics of the system and thus there was no need for cross-validation.

Second, to measure subspace overlaps for d-dimensional neural activities across multiple conditions and time points within coarser time bins (Fig. 4c, Fig. 5f, Fig. 6e, Supplementary Fig. 7d, Supplementary Fig. 8d–e, Supplementary Fig. 11c, Supplementary Fig. 12d, Supplementary Fig. 13d, and Supplementary Fig. 10b,c,f,g), thus corresponding to high-dimensional coding sub-spaces, we used the following properly normalized measure:

$$\text{across-condition variance explained}\left(t, t'\right) = \frac{\mathrm{Tr}\left(\mathbf{U}^\top\left(t'\right)\ \mathbf{\Sigma}(t)\ \mathbf{U}\left(t'\right)\right)}{\mathrm{Tr}\left(\mathbf{P}^\top(t)\ \mathbf{\Sigma}(t)\ \mathbf{P}(t)\right)} \tag{S32}$$

where $\mathbf{\Sigma}(t)$ is the covariance matrix of neural activities across conditions and raw (1-ms) time points within time bin t, the columns of $\mathbf{P}(t)$ are the first principal components of neural activities within time bin t (i.e. the eigenvectors of $\mathbf{\Sigma}(t)$ associated with the largest eigenvalues), and $\mathbf{U}\left(t'\right)$ is the subspace of interest with respect to which overlaps are computed (which itself may or may not depend on time, see below). The time resolution of t and t' (i.e. the duration of time bins within which data was used to compute the corresponding terms at a given t or t'), the choice of $\mathbf{U}\left(t'\right)$, and the number of vectors used for constructing $\mathbf{U}\left(t'\right)$ and $\mathbf{P}(t)$ depended on the analysis (see below).

Specifically, for measuring subspace overlap between neural activity and persistent vs. amplifying modes (Fig. 4c, Fig. 5f, Fig. 6e, Supplementary Fig. 7d, Supplementary Fig. 8d–e, and Supplementary Fig. 10f,g), we set $\mathbf{U}\left(t'\right) = \mathbf{U}$ where the

columns of $\mathbf{U}$ are the first $d/4$ eigenvectors of the most persistent or amplifying subspace (orthogonalized using a QR decomposition for the most persistent modes—this was not necessary for most amplifying modes which are orthogonal by construction), or $d/4$ randomly chosen orthonormal vectors as a control (shown as 'chance'; computed analytically as $1/4$ for 'de novo' linear networks (Fig. 4c and Supplementary Fig. 8d–e), and numerically for fitted linear networks, also yielding values of approximately $1/4$, Fig. 5f, Fig. 6e, Supplementary Fig. 7d, and Supplementary Fig. 10f). $\mathbf{P}(t)$ contained the first $d/4$ principal components. In this case, a value of 1 for this metric implies that the $d/4$ directions of greatest variability in neural activity overlap exactly with the $d/4$-dimensional subspace spanned by $\mathbf{U}$. The time resolution of t was 20 ms (for clarity, bins to be plotted were subsampled in the corresponding figures). Note that when this analysis was performed on linear networks fitted to neural data (experimentally recorded or simulated), $\mathbf{U}$, $\mathbf{P}(t)$, and $\mathbf{\Sigma}(t)$ were all obtained from the same fitted linear network (i.e. no cross-validation). Specifically the parameters of the network were used to determine $\mathbf{U}$ (see Methods S2.4.3), and the neural responses these fitted linear dynamics generated (rather than the original neural responses that were fit by the linear model) were used to determine $\mathbf{\Sigma}(t)$ and thus $\mathbf{P}(t)$. See Methods S2.8 for computing the significance of these overlaps (and their differences). When analysing optimized ring attractor networks (Supplementary Fig. 3e), we used 2-dimensional subspaces (rather than $d/4$-dimensional subspaces) because we found empirically that the obtained ring attractors lay in a 2-dimensional subspace.

For analyzing subspace sharing between different task epochs (Supplementary Fig. 11c, Supplementary Fig. 12d, Supplementary Fig. 13d, and Supplementary Fig. 10b), $\mathbf{U}(t')$ contained the top k principal components (PCs) of neural activity within the time bin indexed by t' (we used $k = 10$ for the monkey data and $k = 4$ for our models because the models typically exhibited lower dimensional dynamics), while $\mathbf{P}(t)$ included all PCs within the time bin indexed by t. For these, we performed principal components analysis with dimensions corresponding to neurons and data points corresponding to time points and cue conditions. The time resolution of both t and t' was 250 ms, such that the time periods (relative to cue onset) that we used were -500 to -250 ms (spontaneous epoch), 0 to 250 (cue epoch), 1250 to 1500 ms (delay epoch), and the first 250 ms after the go cue, i.e. t_{go} to $t_{\text{go}} + 250$ ms (go epoch). In this case, $\mathbf{U}(t')$, $\mathbf{P}(t)$ and $\mathbf{\Sigma}(t)$ were obtained by fitting all the available neural data (i.e. no cross-validation). See also Ref. 7 for an 'alignment index' metric that is closely analogous to this use of this metric.

For showing how much variance the top 2 delay epoch PCs capture over time (Supplementary Fig. 10c), in line with Ref. 4, we set $\mathbf{U}(t') = \mathbf{U}$ where the columns of $\mathbf{U}$ are the first 2 principal components of neural activities over the time period 750 to 250 ms before the go cue, i.e. $t_{\text{go}} - 0.75$ to $t_{\text{go}} - 0.25$ s, and $\mathbf{P}(t)$ also includes the top 2 principal components. The resolution for t was 10 ms (for clarity, bins to be plotted were subsampled in the corresponding figure). In this case, we estimated $\mathbf{U}$ and $\mathbf{P}(t)$ in a cross-validated way (as in Ref. 4)—we estimated $\mathbf{U}$ using training data and $\mathbf{P}(t)$ and $\mathbf{\Sigma}(t)$ using test data, and we show results averaged over 10 random 1:1 train:test splits of the data. See also Ref. 4 for a measure that is closely related to this use of this metric, but uses the number of neurons in the denominator instead of the total variance.

S2.7.4 Linear decoding

We fitted decoders using linear discriminant analysis to decode the stimulus cue identity from neural firing rates (Fig. 2e,f,k,l, Fig. 4a,b, Fig. 5b,c, Fig. 6c,d, Supplementary Fig. 7c, Supplementary Fig. 3d, Supplementary Fig. 8a–c,Supplementary Fig. 10a,h, Supplementary Fig. 11a,b, Supplementary Fig. 12b,c, and Supplementary Fig. 13b,c). We constrained the decoders to be 2-dimensional (in line with previous studies (4)) because this was a sufficient dimensionality to decode responses. We primarily considered two types of decoding analyses: we either trained decoders on late delay activity and tested on all time points ('delay-trained decoder', e.g. Fig. 4a), or we trained decoders separately at every time point and tested on all times ('full cross-temporal decoding', e.g. Fig. 4b). In all cases, we measured decoding performance in a cross-validated way, using separate sets of neural trajectories to train and test the decoder, and we show results averaged over 10 random 1:1 train:test splits of the data. For delay-trained decoders, training data consisted of pooling neural activity over a 500 ms time interval (the time interval is shown by a horizontal black bar in all relevant figures), and tested the thus-trained decoder with data in each 1 ms time bins across the trial (for clarity, test bins to be plotted were subsampled every 10 ms in the corresponding figures). For full cross-temporal decoding, we binned neural responses into 10 ms time bins and trained and tested on all pairs of time bins (specifically, we plotted mean decoding performance across the 10 1-ms raw time bins corresponding to each 10-ms testing bin). We used a shrinkage (inverse regularisation parameter on the Euclidean norm of decoding coefficients) of 0.5. Chance level decoding was defined as $1/C$, where $C = 2$ or 6 is the number of cue conditions that need to be decoded (Tables S1 and S3).

S2.7.5 Quality of fit for linear models fitted to neural responses

When fitting linear models to neural data (experimentally recorded or simulated; Methods S2.4.3) we used a cross-validated approach for measuring the quality of our fits, with a random 1:1 train:test split of the data (Fig. 5d). For this, we first fitted the model on training data ($\mathbf{x}_*^{(c)} = \mathbf{x}_{\text{train}}^{(c)}$ in Eq. S9). The quality of fit was then computed on the test data, $\mathbf{x}_{\text{test}}^{(c)}$, as the fraction of variance of $\mathbf{x}_{\text{test}}^{(c)}(t)$ explained by the simulated responses (after the appropriate projection, i.e. $\mathbf{C}\,\mathbf{x}^{(c)}(t)$), across

all 20 dimensions weighted by $\mathbf{D}$ (all parameters, including $\mathbf{P}$, $\mathbf{C}$ and $\mathbf{D}$, were set to their values obtained by fitting the training data). In other words, we computed the Pearson R^2 with respect to the identity line using the mean squared error, ε^2 in Eq. S8, with the momentary error in Eq. S9 computed using $\mathbf{x}_*^{(c)} = \mathbf{x}_{\text{test}}^{(c)}$. Once the quality of fit for this split was thus established, we conducted all further analysis involving fitted linear models with the model that was fit to the training half of this split.

As a meaningful lower bound on our quality of fit measure, we also computed the same measure (i.e. fitting a linear neural networks to training data and calculating the quality of fit using test data) for 100 different time-shuffled controls of the original train:test split of the data (Methods S2.4.3), such that we shuffled time bins coherently between the training and the test data, across neurons and conditions (Fig. 5d, dark gray).

To calibrate how much our fits were limited by the noisiness of the data, we also computed the quality of fit directly between $\mathbf{x}_{\text{train}}^{(c)}(t)$ and $\mathbf{x}_{\text{test}}^{(c)}(t)$ (i.e. using the mean squared error, ε^2 in Eq. S8, with the momentary error redefined as $\mathbf{e}^{(c)}(t) = \mathbf{x}_{\text{train}}^{(c)}(t) - \mathbf{x}_{\text{test}}^{(c)}(t)$) for 100 random 1:1 train:test splits of the data (Fig. 5d, light gray). The extent to which the R^2 computed with this control was below 1 reflected the inherent (sampling) noise of the experimental data that limited the quality of fit obtainable with any parametric model, including ours that was based on linear dynamics. Moreover, a cross-validated R^2 computed with our fits that was higher than the R^2 obtained with this control (Fig. 5d dark and light blue vs. light gray) meant that the inherent assumption of linear dynamics in our model acted as a useful regularizer to prevent the overfitting that this overly flexible control inevitably suffered from. See more in Methods S2.8 on statistical testing for our quality of fit measure.

When fitting to simulated neural data, we obtained high quality of fits using the same measure ($R^2 > 0.95$).

S2.7.6 Overlap between the coding populations during the cue and delay epochs

To test whether separate neural populations encode stimulus information during the cue and delay epochs (Supplementary Fig. 10e), we trained (non-cross validated) decoders to decode cue identity using logistic regression on either cue-epoch activity ('cue-trained'; the first 250 ms of activity after cue onset) or delay-epoch activity ('delay-trained'; 1250–1500 ms after cue onset). We used an L2 regularisation penalty of 0.5 (we also tested other regularisation strengths and observed no substantial changes in our results). We took the absolute value of decoder weights as a measure of how strongly neurons contributed to decodability (either positively or negatively). We then binarized the absolute 'cue-trained' and 'delay-trained' weights using their respective median values as the binarization threshold. (This binarization reduces a potential bias effect from large or small weight values in our analysis.) Our measure of overlap between the coding populations during the cue and delay epochs, was then simply the inverse normalized Hamming distance between these two sets of binarized weights:

$$\text{overlap} = 1 - \left\langle \left| w_{n,c}^{\text{cue}} - w_{n,c}^{\text{delay}} \right| \right\rangle_{n,c} \tag{S33}$$

where $w_{n,c}^{\text{cue}}$ ('cue trained') and $w_{n,c}^{\text{delay}}$ ('delay trained') is the binarized weight of neuron n in cue condition c during the cue and delay epochs, respectively, and $\langle\cdot\rangle_{n,c}$ denotes taking the mean across neurons and cue conditions. For completely overlapping populations, this measure takes a values of 1, for completely non-overlapping populations, it takes a values of 0, and for random overlap (shown as 'chance') it takes a values of 0.5.

For the shuffle controls, we randomly permuted the neuron indices of the delay-trained weights (such that using the median as a threshold thus resulted in values close to 0.5, i.e. chance level; Supplementary Fig. 10e). We show results (for both the original analysis and shuffle control) for 10 random halves of the data (equivalent to the training halves of 10 different 1:1 train:test splits). We also tested a variety of percentile values other than the median and our results did not change substantially (choosing a threshold other than the median causes both the data and shuffle controls to have overlap values lower than those that we obtained with the median as the threshold, but it does not substantially affect the difference between them).

S2.7.7 Finding fixed / slow points

For finding the fixed / slow points of nonlinear network dynamics (Fig. 2d,j, Supplementary Fig. 2b,f, Supplementary Fig. 3a, and Supplementary Fig. 11d), we used a slow-point analysis method (21) that searches for an $\mathbf{x}$ for which the L2 norm of the gradient determined by the autonomous dynamics of the network is below a threshold. Note that this was only possible in model neural networks as the method requires access to the equations (and parameters) defining the true (nonlinear) dynamics of a system.

Specifically, for network dynamics governed by (cf. Eqs. S1 and S2)

$$\frac{\mathrm{d}\mathbf{x}(t)}{\mathrm{d}t} = \psi\big(\mathbf{x}(t)\big) \ , \tag{S34}$$

for some function ψ, we sought to find points $\mathbf{x}^*$ such that $\|\psi(\mathbf{x}^*)\|_2$ is small. To achieve this, we drew 1000 $\mathbf{x}$'s from a spherical Gaussian distribution with mean 0 and variance 10 (the large variance helps to ensure that we cover a large part of state space) and we optimized each $\mathbf{x}$ to minimize $\|\psi(\mathbf{x})\|_2$ using gradient descent with gradients obtained by back-propagation with an Adam optimizer (20). We used an adaptive learning rate (which we found worked substantially better than a fixed learning rate in this scenario) that started at 0.1 and halved every 1000 training iterations (we used 5000 training iterations in total). Finally, we identified the $\mathbf{x}$'s obtained at the end of optimization as asymptotically stable fixed points, $\mathbf{x}^*$, if $\|\psi(\mathbf{x})\|_2 < 0.001$ and if the largest real part in the eigenvalues of the linearization of $\psi(\mathbf{x})$ around $\mathbf{x}^*$ was less than 0.

S2.7.8 Correlations between initial and final neural firing rates

To measure correlations between initial and final simulated activities, we used the Pearson correlation coefficient (with respect to the identity line) between initial and final mean-centered firing rates across neurons within the same simulation (i.e. no cross-validation; Fig. 2b,h; insets). Histograms show the distribution of this correlation across 6 cue conditions (and the 10 different networks, each simulated 10 times, see above) using a kernel-density estimate (Fig. 2c,i, Supplementary Fig. 2c,d,g,h, and Supplementary Fig. 3c).

S2.7.9 Measuring non-normality using Henrici's index

When measuring the non-normality of our fitted linear models to our neural recordings (Fig. 5d–f) we used Henrici's index (31) $h = \left(\sqrt{\|\mathbf{J}\|_F^2 - \sum_i |\lambda_i|^2}\right) / \|\mathbf{J}\|_F$, where $\mathbf{J} = \mathbf{W} - \mathbf{I}$ is the Jacobian of the dynamics, λ_i are the eigenvalues of $\mathbf{J}$, and $\|\cdot\|_F$ is the Frobenius norm of a matrix. An index of 0 implies that the system is normal whereas an index of 1 implies that the system is maximally non-normal.

S2.8 Statistics

We performed statistical hypothesis testing in two cases.

First, we tested whether the quality of fit of linear models to experimental data was sufficiently high using permutation tests. To construct the distribution of our test statistic (cross-validated R^2, see also Methods S2.7.5) under the null hypothesis, we used $n = 200$ different random time shuffles of the data (Fig. 5d, dark gray), such that we shuffled time bins coherently between the training and the test data, across neurons and conditions, and for each shuffle used the same random 1:1 train:test split as for the original (unshuffled) data. For additional calibration, we also constructed the distribution of our test statistic under the alternative hypothesis that all cross-validated errors were due to sampling noise differences between the train and test data. For this, we used $n = 200$ random 1:1 train:test splits of the (original, unshuffled) data, and measured the quality of fit directly between the test data and the training data (rather than a model fitted to the training data, see also Methods S2.7.5; Fig. 5d, light gray). In both cases, we computed the two-tailed p-value of the test statistic as computed on the real data (Fig. 5d, blue lines) with respect to the corresponding reference distribution.

Second, we also used a permutation test-based approach to test whether the experimentally observed overlaps with persistent and amplifying modes (or their differences) were significantly different from those expected by chance. For testing the significance of overlaps in a given time step, we constructed the distribution of our test statistics (the overlap measures; Methods S2.7.3) under the null hypothesis by generating $n = 200$ random subspaces within the space spanned by the 20 PCs we extracted from the data (Methods S2.4.3), dimensionality matched to the persistent and amplifying subspaces (i.e. 5 orthogonal dimensions), and computed the same subspace overlap measures for the data in the given time step with respect to these random subspaces (Fig. 5f and Supplementary Fig. 10f–g; gray line and shading). For testing the significance of differences between overlaps (amplifying vs. persistent at a given time step, or amplifying or persistent between two different time steps), our test statistic was this difference (i.e. a paired test), and our null distribution was constructed by measuring it for $n = 200$ pairs of random subspace overlaps at the appropriate time step(s). Once again, in all these cases we computed the two-tailed p-value of the test statistic as computed on the real data (Fig. 5f and Supplementary Fig. 10f–g, green and red lines) with respect to the corresponding reference distribution.

Note that we did not compute p-values across multiple splits of the data because this led to p-value inflation as we increased the number of splits. Instead, we repeated all relevant analyses on 10 different random 1:1 train:test splits to see if our results were robust to the choice of data split. Indeed, we obtained qualitatively and quantitatively (in terms of p-values for quality of fits, and overlaps) similar results for all these splits.

Permutation tests do not assume that the data follows any pre-defined distribution. No statistical methods were used to predetermine experimental sample sizes. Sample sizes for permutation tests (n above) were chosen so as to be able to determine p-values to a precision of 0.01 (quality of fits) or 0.01 (subspace overlaps).

S3 Supplementary text

The main insight of our work is that optimal information loading relies on inputs aligned with the most amplifying mode of network dynamics, which in turn accounts for the combination of stable and dynamic coding seen in experiments. In Section S4, we first show that this combination does not arise in previously proposed models of working memory with hand-crafted connectivities, but does in task-optimized neural networks. To understand why network optimization leads to this behavior, in Section S5, we provide mathematically rigorous derivations of optimal information loading for linear network dynamics. However, these derivations—and indeed, the very concept of the most amplifying mode—do not readily apply to nonlinear networks, which are likely to be more relevant for understanding cortical circuits. Therefore, in Section S6 we generalize the concept of the most amplifying mode to nonlinear dynamical systems (through a local linearization around the ground state) and show that in a simple canonical nonlinear attractor system it offers the same qualitative advantage for information loading as in linear networks. In Section S7, we demonstrate the same advantage in high-dimensional multi-attractor nonlinear networks, show that the abstract notion of information loading performance we used in simple systems predicts a practically more relevant measure of performance in these networks, and that the dynamical signatures we established for identifying optimal information loading in linear networks can still be used even when the real dynamics of a circuit are nonlinear, and only indirectly accessible to us by the neural responses they generate. As there are no readily available analytical techniques that could be directly applied to the problem of information loading in nonlinear dynamical systems, our analyses in Sections S6 and S7 are mostly based on numerical simulations of specific nonlinear dynamical systems where we have ground truth knowledge of the dynamics and the information loading strategy that is optimal for them. Finally, in Section S8, we show with analytical derivations that optimizing a ring attractor with a negative cosine loss on the difference between true and decoded angles (as we do in the main text) is approximately equivalent to optimizing the population Fisher information about angle in the network.

S4 Comparison of previous models of working memory with task-optimized networks

We simulated four, previously proposed representative models of working memory, as well as one of the task-optimized networks developed here for comparison (the just-in-time network in Fig. 6d, right).

First, we simulated the 'bump' attractor network of Ref. 1: a recurrent network in which neurons are indexed by their preferred directions and are connected recurrently by a circulant weight matrix, such that the connection strength between two excitatory neurons only depended (as a circular Gaussian) on the difference of their preferred directions, and there was a single inhibitory neuron providing global inhibition (Methods S2.5). (Note that this network thus represents a paradigmatic case of quasi-symmetric connectivity.) In the absence of inputs, this network is able to maintain the memory of a saccade direction via a 'bump' of activities: its intrinsic dynamics (mediated by the recurrent interactions between its neurons) converge to a steady state pattern in which the response of neurons is described by a Gaussian-like ('bump') function of the difference between their preferred direction and the maintained direction. To understand information loading and static vs. dynamic coding in this network, it is useful to adopt a state space view of its dynamics (cf. Fig. 3). Information about the to-be-memorised direction (the stimulus cue) is loaded into this network by a transient external input. During the cue period, this transient input drives the network (Supplementary Fig. 1a, left, pale purple line and arrow) into a suitable state (Supplementary Fig. 1a, left, pale purple circle). Then, during the delay period, in the absence of the cue, its intrinsic dynamics (Supplementary Fig. 1a, left, dark purple line), are 'attracted' into a cue-specific steady state (corresponding to a bump of activities; Supplementary Fig. 1a, left, black cross). This naturally accounts for selective persistent activity (Supplementary Fig. 1a, center). However, the state into which the external input drives network activities, i.e. the initial state during the delay period, already has large overlap with the steady state to which the dynamics eventually converge (Supplementary Fig. 1a, left, pale purple line points in the direction of the black cross). In other words, the network performs simple 'pattern completion' (32), whereby this overlap is only slightly improved until it becomes perfect (Supplementary Fig. 1a, left), with the response of each neuron quickly reaching its steady-state and maintaining it throughout most of the delay period (Supplementary Fig. 1a, center). As a result, neurons show limited transient activity during the delay period (Supplementary Fig. 1a, center), and cross-temporal decoding reveals stable coding throughout the whole trial, lacking the characteristic dynamic coding seen in experimental data (compare Fig. 1b to Supplementary Fig. 1a, right).

We also simulated the closely related 'discrete' attractor network of Ref. 1. In this network, neurons are assigned to a finite number of discrete clusters (one cluster for each cue condition), with strong recurrent connections between neurons belonging to the same cluster, and weaker connection between neurons belonging to different clusters (Methods S2.5). Thus, instead of maintaining bumps of activity, the steady states of this network correspond to one of the clusters being active while all others exhibiting weak background activity. Other than that, the same principles of simple pattern completion apply to information loading that we saw in the bump attractor network (Supplementary Fig. 1a). Therefore, this network is also characterized by the absence of dynamic coding (Supplementary Fig. 1b,c).

Recent work has suggested that linear integrator networks, that are based on similar principles as nonlinear attractor networks but use linear dynamics, can exhibit activity transients similar to those seen in experiments (4) (see also (25)). In line

with earlier work, when simulating such models, we also found activity transients to emerge when cue-dependent inputs included a random component in addition to the 'usual' steady state-aligned component that ensured pattern completion (Methods S2.5). Because the random component in these networks is designed to be orthogonal to the steady state-aligned component, the dynamics of the network take time to relax to the subspace spanned by the cue-dependent steady states (the coding subspace; Supplementary Fig. 1c, left), and neurons exhibit transient activities during this relaxation (Supplementary Fig. 1c, middle). However, precisely because this relaxation thus takes place in a subspace orthogonal to the persistent subspace, it does not affect stimulus coding during the delay. Thus a delay-trained decoder will always generalize perfectly to the cue period. Indeed, we found that cross-temporal coding demonstrated strongly stable coding (Supplementary Fig. 1c, right, also see Supplementary Fig. 8a)—just as in the classical attractor networks seen above. We were able to achieve more dynamic coding in a modified version of this model, in which the random component of the inputs completely dominated (Methods S2.5.3, Supplementary Fig. 8b). In other words, this modified model consisted of unconstrained (non-normal) connectivity and purely random inputs (similar to Fig. 4, 'random'). Nevertheless, our neural measure of optimal information loading, based on the time course of the overlap of activities with the most amplifying and most persistent modes (Supplementary Fig. 8d,e) still revealed a fundamental difference from experimental data (cf. Fig. 5f) and task-optimized neural networks (cf. Fig. 6e).

A major class of alternative models of working memory use essentially feedforward dynamics (5, 6). We thus simulated a simple, purely feedforward network that captured the essence of such dynamics (Methods S2.5). As expected, this network did not exhibit any steady state and inputs that stimulated the beginning of the feedforward chain resulted in long transients that rapidly transitioned into activity patterns that were orthogonal to these inputs (because they involved the firing of an altogether different group of neurons at the end of the chain)—after which, ultimately, all activities decayed to zero (Supplementary Fig. 1d, left and middle). (Note that the orthogonality of successively expressed activity patterns, and the eventual decay of activities, would remain the same even in the more general case when the nodes of the feedforward chain are distributed patterns of neural activities, rather than individual neurons.) As a consequence, this network showed strong dynamic coding with cross-temporal decoding being high only between neighbouring time-points, but no stable coding during the delay period (Supplementary Fig. 1d, right).

Finally, for comparison, we show the same plots for a representative example of one of our task-optimized networks (Supplementary Fig. 1e; see also Fig. 6, 'just-in-time'). In this network, inputs are also largely orthogonal to the late delay activities, as in the feed-forward networks, but eventually converge to an attractor maintaining information during the late delay period, as in attractor networks (Supplementary Fig. 1e, left). As a consequence, neurons show rich transient dynamics (Supplementary Fig. 1e, middle), and the network exhibits the experimentally described combination of dynamic and stable coding (Supplementary Fig. 1e, right).

S5 Analysis of linear integrator networks

In this section we provide analytical derivations showing that optimal information loading is achieved by the most amplifying mode in linear integrator networks. We first define linear integrator networks and introduce the basic mathematical concepts for understanding their dynamics (Section S5.1). We then define the persistent mode, a direction in the state space of linear integrator networks that is critical for characterising their performance (Section S5.2). We also define the energy of the system as a quantity useful for both constraining optimisation and defining optimal information loading (Section S5.3). We then define optimal information loading in five seemingly different ways (via five different constrained optimization problems), but show that all definitions lead to the same solution (at least approximately):

1. The initial condition with a fixed norm that achieves the highest asymptotic overlap with the persistent mode (Section S5.4).
2. The initial condition with minimal norm that achieves a fixed asymptotic overlap with the persistent mode (Section S5.5).
3. The most amplifying mode, defined as the initial condition with a fixed norm that evokes the largest asymptotic energy (Section S5.6).
4. The initial condition with a fixed norm that provides maximal 'total' decodability in a *noisy* linear integrator over an asymptotically long time horizon (Section S5.7).
5. The initial condition that achieves a fixed asymptotic overlap with the persistent mode while using the least amount of (asymptotic) energy (Section S5.8).

S5.1 Linear integrator networks

We define a linear integrator network as a linear dynamical system

$$\dot{\mathbf{x}}(t) = \mathbf{J}\,\mathbf{x}(t) \tag{S35}$$

with a Jacobian $\mathbf{J}$, which we define using its eigen-decomposition:

$$\mathbf{J} = \mathbf{V}\,\boldsymbol{\Lambda}\,\mathbf{U} \tag{S36}$$

where $\boldsymbol{\Lambda}$ is a diagonal matrix containing the eigenvalues of $\mathbf{J}$, $\lambda_1, \dots \lambda_N$ (where N is the number of neurons in the network, and the eigenvalues are sorted in descending order of their real values, without loss of generality), and $\mathbf{V}$ is the matrix of the associated (right) eigenvectors of $\mathbf{J}$, $\mathbf{v}_{(1)}, \dots \mathbf{v}_{(N)}$, as columns (unit-norm, without loss of generality). Conversely, $\mathbf{U} = \mathbf{V}^{-1}$ is the matrix of the corresponding left eigenvectors, $\mathbf{u}_{(1)}^\top, \dots \mathbf{u}_{(N)}^\top$, as rows. Note that typical dynamics for a linear neural network (cf. Eq. S1) include a leak term and a time constant, τ, such that $\mathbf{J} = \frac{1}{\tau}(\mathbf{W} - \mathbf{I})$, where $\mathbf{W}$ is the recurrent weight matrix of the network (and we ignored noise and temporally extended external inputs to the network in order to focus on the intrinsic deterministic component of its dynamics).

Note that the eigenvectors in $\mathbf{V}$ need not be linearly independent (orthogonal): they are only orthogonal in the special case when $\mathbf{J}$ is a normal matrix (e.g. symmetric), in which case $\mathbf{U} = \mathbf{V}^\top$, i.e. the left and right eigenvectors are identical. Otherwise, when $\mathbf{J}$ is non-normal, its eigenvectors are not orthogonal, and $\mathbf{U} \neq \mathbf{V}^\top$, i.e. the left and right eigenvectors are different. This will be important below for understanding the geometry of optimal information loading in the network.

In order for a linear dynamical system to act as an integrator network, we require that its top eigenvalue (eigenvalue with the largest real part), λ_1, is real, unique, and marginally stable

$$\lambda_1 = -\delta \text{ (with } \delta \to 0) \tag{S37}$$

and all of its other eigenvalues are properly stable with real parts that are

$$\mathrm{Re}(\lambda_i) \ll 0 \text{ (for } i > 1) \tag{S38}$$

(This is consistent with our procedure for constructing the linear integrator networks analysed in Figs. 3 and 4 and Supplementary Figs. 4 and 5, see Methods S2.4.1, and Table S1.)

Following standard textbook treatment (27), we often make use of the eigen-decomposition in Eq. S36 to write the state of the system in terms of its eigen-coefficients as

$$\mathbf{y}(t) = \mathbf{U}\,\mathbf{x}(t) \text{ , or, equivalently, } \mathbf{x}(t) = \mathbf{V}\,\mathbf{y}(t) \tag{S39}$$

which in turn allows us to rewrite the dynamics of the system in Eq. S35 as

$$\dot{\mathbf{y}}(t) = \boldsymbol{\Lambda}\,\mathbf{y}(t) \tag{S40}$$

Indeed, the solution to the linear dynamics of the system (Eq. S35) in the original space of neural activities is

$$\mathbf{x}(t) = e^{\mathbf{J}\,t}\,\mathbf{x}(0) \tag{S41}$$

which expresses complex interactions between neurons (in the first term). However, in the space of the eigen-coefficients, the solution of the dynamics (Eq. S40) can be conveniently written as a set of *independently* evolving components, each decaying exponentially to zero with a rate given by the corresponding eigenvalue:

$$y_i(t) = y_i(0)\; e^{\lambda_i\,t} \tag{S42}$$

In the following, we will be particularly interested in the asymptotic behavior of the system, $t \to \infty$. Given the marginally stable nature of the system (Eqs. S37 and S38), in this joint limit all but the first eigencoefficient decay to zero:

$$e^{\delta\,t}\; y_i(t) \to \begin{cases} y_1(0) & \text{for } i = 1 \\ 0 & \text{otherwise} \end{cases} \tag{S43}$$

S5.2 The persistent mode

The persistent mode of the system is the direction in state space with which the system's state, $\mathbf{x}(t)$ is aligned in the asymptotic limit, $t \to \infty$. As such, a natural measure of the system's performance will be its position along this direction (Section S5.4).

We define the momentary direction of the system's state as

$$\bar{\mathbf{x}}(t) = \frac{\mathbf{x}(t)}{\sqrt{\mathbf{x}^\top(t)\ \mathbf{x}(t)}} \tag{S44}$$

which we can rewrite using the eigen-coefficients (Eq. S39) as

$$\bar{\mathbf{x}}(t) = \frac{\mathbf{V}\,\mathbf{y}(t)}{\sqrt{\mathbf{y}^\top(t)\ \boldsymbol{\Xi}\,\mathbf{y}(t)}} \tag{S45}$$

$$\text{where } \boldsymbol{\Xi} = \mathbf{V}^\top\mathbf{V} \tag{S46}$$

measures the overlap between the eigenvectors. Taking the asymptotic limit as defined above (Eq. S43), leads us to

$$\bar{\mathbf{x}}(t) \to \mathbf{v}_{(1)} \tag{S47}$$

In other words, asymptotically, the network expresses an activity pattern that is aligned with $\mathbf{v}_{(1)}$, the first *right* eigenvector of the Jacobian, which we thus call its *persistent mode*. In control theoretic terms, this shows (the unsurprising result) that the persistent mode of a linear integrator network is its most 'controllable' mode (33).

S5.3 The energy of the system

As we will see below, an important quantity for constraining and understanding network dynamics is the 'energy' of the system, which is the total norm of neural responses integrated over some (potentially infinite) time window. We define the total 'energy' that the system has used up to time t as:

$$\varepsilon(t) = \int_0^t \mathbf{x}^\top\!\left(t'\right)\ \mathbf{x}\!\left(t'\right)\ \mathrm{d}t' \tag{S48}$$

which we can re-write using the solution to the dynamics in Eq. S41 as

$$\varepsilon(t) = \mathbf{x}^\top(0) \int_0^t e^{\mathbf{J}^\top t'}\ e^{\mathbf{J}\,t'}\ \mathrm{d}t'\ \mathbf{x}(0) \tag{S49}$$

Using the eigen-coefficients (Eq. S39), and the overlap of eigenvectors as defined in Eq. S46, we can further rewrite this as

$$\varepsilon(t) = \int_0^t \mathbf{y}^\top\!\left(t'\right)\ \boldsymbol{\Xi}\,\mathbf{y}\!\left(t'\right)\ \mathrm{d}t' \tag{S50}$$

Using Eq. S42 to solve the dynamics in the space of eigen-coefficients analytically allows us to write the energy in closed form as an explicit function of the initial condition:

$$\varepsilon(t) = \mathbf{y}^\top(0)\ \boldsymbol{\Omega}(t)\ \mathbf{y}(0) \tag{S51}$$

$$\text{where } \Omega_{ij}(t) = \frac{\Xi_{ij}}{\lambda_i + \lambda_j}\left[e^{(\lambda_i+\lambda_j)\,t} - 1\right] \tag{S52}$$

Taking the asymptotic limit (Eq. S43) of Eq. S49, we obtain in the original space of neural responses

$$\varepsilon(t) \to \varepsilon_\infty = \mathbf{x}^\top(0)\ \mathbf{Q}\,\mathbf{x}(0) \tag{S53}$$

$$\text{where } \mathbf{Q} = \int_0^\infty e^{\mathbf{J}^\top t}\, e^{\mathbf{J}\,t}\,\mathrm{d}t \tag{S54}$$

is the so-called 'observability' Gramian of the dynamics (33). We can once again rewrite this by substituting the asymptotic eigen-coefficients of marginally stable dynamics (Eq. S43) into Eq. S51 as

$$\varepsilon_\infty = \frac{1}{2\,\delta}\, y_1^2(0) \propto \mathbf{x}^\top(0)\ \mathbf{u}_{(1)}\,\mathbf{u}_{(1)}^\top\,\mathbf{x}(0) \tag{S55}$$

where $\mathbf{u}_{(1)}$ is the first column of $\mathbf{U}$, i.e. the first *left* eigenvector of $\mathbf{J}$.

S5.4 Optimal information loading: maximising asymptotic persistent overlap with fixed initial norm

We now study the effects of using different inputs on the asymptotic behaviour of the system. For the purposes of the analyses presented here, by 'input', we mean the initial condition of the system, $\mathbf{x}(0)$. (For numerical results with temporally extended inputs, see Fig. 4, and also Fig. 6 and Supplementary Figs. 11–13 for nonlinear networks using temporally

extended inputs). In the previous subsection we saw that asymptotically the system's state is always aligned with the persistent mode, independent of its initial condition. However, the precise point along that mode, i.e. the system's overlap with the persistent mode, still depends on the initial condition.

We define the momentary overlap with the persistent mode as

$$p(t) = \mathbf{v}_{(1)}^{\top}\, \mathbf{x}(t) \tag{S56}$$

Using the eigen-coefficients (Eq. S39), we once again rewrite this in closed form as an explicit function of the initial condition:

$$p(t) = \sum_i y_i(0)\; \Xi_{i1}\; e^{\lambda_i t} \tag{S57}$$

and take the asymptotic limit (Eq. S43) to obtain (see also Ref. 21 for an analogous derivation):

$$p(t) \to p_\infty = e^{-\delta t}\, y_1(0) \propto \mathbf{u}_{(1)}^{\top}\, \mathbf{x}(0) \tag{S58}$$

In order to study optimal information loading, we want to identify the initial condition, $\mathbf{x}(0)$, that achieves the maximal asymptotic overlap with the persistent mode. As the system has linear dynamics, it is obvious that $p(t)$ simply scales linearly with the norm of $\mathbf{x}(0)$. Therefore, to make our question meaningful, we have to introduce some additional constraint that prevents the trivial solution of an input with an infinitely large norm. We first consider simply a norm-constraint directly on $\mathbf{x}(0)$. This leads to solving the following optimization problem

$$\mathbf{x}^*(0) = \underset{\mathbf{x}(0)}{\operatorname{argmax}}\, \underbrace{\mathbf{u}_{(1)}^{\top}\, \mathbf{x}(0)}_{\propto\, p_\infty},\ \text{s.t. } \|\mathbf{x}(0)\|_2 = 1 \tag{S59}$$

of which the solution is trivially

$$\mathbf{x}^*(0) = \pm\mathbf{u}_{(1)} \tag{S60}$$

Thus, optimal information loading is achieved by the first *left* eigenvector of $\mathbf{J}$ (which, as we saw above, will *not* be identical to the first *right* eigenvector, $\mathbf{v}_{(1)}$, i.e. the persistent mode itself, in the general case, when $\mathbf{W}$—and thus $\mathbf{J}$—is non-normal).

S5.5 Optimal information loading: achieving fixed asymptotic persistent overlap with a minimal initial norm

Defining optimal information loading instead as the input that achieves a fixed asymptotic overlap with the minimal norm, i.e. as the solution to the optimization problem

$$\mathbf{x}^*(0) = \underset{\mathbf{x}(0)}{\operatorname{argmin}}\, \|\mathbf{x}(0)\|_2,\ \text{s.t. } p_\infty = \text{const.} \tag{S61}$$

trivially leads to the same solution (up to multiplicative scaling) as that which we saw in Section S5.4, as it amounts to optimising the same Lagrangian:

$$\mathbf{x}^*(0) = \pm\mathbf{u}_{(1)} \tag{S62}$$

We will also consider below a third way to constrain the optimization problem, via its 'energy' (time integrated-norm), rather than just the norm of its initial condition (Section S5.8).

S5.6 Optimal information loading: maximising asymptotic energy with fixed initial norm

The asymptotic energy we introduced above (Eq. S55) allows us to relate optimal information loading to a well-known concept in control theory: the most amplifying mode, which is the eigenvector of the observability Gramian associated with the largest eigenvalue (30, 33, 34). Crucially, the most amplifying mode can also be defined as the initial condition with a unit norm that evokes the largest asymptotic energy, i.e. as the solution to the optimization problem

$$\mathbf{x}^*(0) = \underset{\mathbf{x}(0)}{\operatorname{argmax}}\, \underbrace{\mathbf{x}^{\top}(0)\; \mathbf{u}_{(1)}\, \mathbf{u}_{(1)}^{\top}\, \mathbf{x}(0)}_{\propto\, \mathcal{E}_\infty},\ \text{s.t. } \|\mathbf{x}(0)\|_2 = 1 \tag{S63}$$

which is straightforwardly given as

$$\mathbf{x}^*(0) = \pm\mathbf{u}_{(1)} \tag{S64}$$

Comparing Eq. S64 with Eq. S60 reveals that, in the case of 1-dimensional linear integrators, the most amplifying mode achieves optimal information loading as it is equivalent to the top left eigenvector. The intuition behind this result is simple:

- Optimal information loading is defined (Section S5.4) as using an initial condition that (asymptotically) generates maximal activity along the persistent mode.
- The most amplifying mode is formally defined as the direction for initial conditions that results in the largest overall fluctuations (on an infinite time horizon; Eq. S63).
- In linear integrators, all fluctuations are eventually quenched, except those that are expressed along the persistent mode of the system (Eq. S43).
- Therefore, if an input direction generates large overall fluctuations, it must be because it achieves a large asymptotic overlap with the persistent mode.
- Therefore, the most amplifying mode must achieve the largest overlap with the persistent mode, i.e. it is the optimal information loading direction.

More importantly, as we show in Section S5.7, the most amplifying mode generalises to cases (multiple integration directions in non-perfect integrators) in which the left eigenvectors are not relevant, thus motivating our use of the set of most amplifying directions rather than left eigenvectors to define optimal information loading throughout our analyses.

S5.7 Optimal information loading: noise-robust decoding

We now study the more general problem of optimal inputs for general, stable linear dynamics, which are not necessarily exactly marginally stable, and may in fact have multiple dimensions along which they (approximately) integrate. To be closer to the actual (linear) networks we study in the main text (Fig. 4, Supplementary Fig. 5c,d, and Supplementary Fig. 8a,b,d,e), we consider stochastic dynamics of the form (cf. Eq. S35, but also see Eq. S2):

$$\dot{\mathbf{x}}(t) = \mathbf{J}\,\mathbf{x}(t) + \sigma\,\boldsymbol{\eta}(t) \tag{S65}$$

where $\boldsymbol{\eta}(t)$ is zero-mean, unit-variance white Gaussian noise, so that the total noise injected into the system has variance σ^2. We assume that the network has attained its (input-free) steady-state distribution before the stimulus arrives at $t = 0$, and the input $\mathbf{x}(0)$ is an instantaneous additive 'push' of the network state from whatever stochastic state it takes at $t = 0$ (rather than directly defining the network's state at $t = 0$ as before).

Considering stochastic network dynamics allows us to meaningfully define optimality in terms of decoding performance (rather than the somewhat arbitrary objective of maximising overlap with a particular direction in state space) — also in closer analogy with the main text. Following Ref. 6, we quantify decoding performance using the Fisher information. For this, we first note that as the dynamics is stochastic, the state of the system at any time is described by a probability distribution. Given that the dynamics is linear and the noise is Gaussian (Eq. S65), this distribution is also Gaussian:

$$\mathbf{x}(t) \sim \mathcal{N}\left(e^{\mathbf{J}\,t}\,\mathbf{x}(0)\,,\mathbf{C}\right) \tag{S66}$$

where $\mathbf{C} = \sigma^2 \int_0^\infty e^{\mathbf{J}\,t'}\, e^{\mathbf{J}^\top t'} \mathrm{d}t'$ is the stationary noise covariance of neural responses. (Note that the mean of the state distribution is the same as that for deterministic dynamics, Eq. S41, so that noise only contributes to the covariance, and conversely, the covariance does not depend on the input $\mathbf{x}(0)$.)

The ability to discriminate two inputs in the $\mathbf{x}(0)$ direction, $\mathbf{x}(0)$ and $\mathbf{x}(0)\ (1 + \Delta)$, at a later time t, is upper bounded by the Kullback-Leibler (KL) divergence between the two corresponding state distributions at that time. For normal distributions that only differ in their means (Eq. S66), the KL-divergence can be written simply as:

$$\mathrm{D_{KL}}(t) = \frac{1}{2}\Delta^2\,\mathbf{x}^\top(0)\;\mathcal{I}(t)\;\mathbf{x}(0) \tag{S67}$$

where $\mathcal{I}(t)$ is the Fisher information matrix of the system at time t:

$$\mathcal{I}(t) = e^{\mathbf{J}^\top t}\,\mathbf{C}^{-1}\,e^{\mathbf{J}\,t} \tag{S68}$$

Indeed, this upper bound on decoding performance is saturated by a simple linear decoder, $\hat{\mathbf{x}}(t) = \mathbf{W}_{\mathrm{out}}\,\mathbf{x}(t)$ with readout weights

$$\mathbf{W}_{\mathrm{out}} = \mathbf{C}^{-1}\,e^{\mathbf{J}\,t}\,\mathbf{x}(0) \tag{S69}$$

Note that the optimal decoder is thus time-*dependent*.

Finally, to create a single scalar objective that can be optimised, we measure the 'total' decodability of the system over a suitably long (technically, infinite) time horizon:

$$\bar{\mathrm{D}}_{\mathrm{KL}} = \frac{1}{2}\Delta^2\,\mathbf{x}^\top(0)\;\bar{\mathcal{I}}\,\mathbf{x}(0) \tag{S70}$$

where $\bar{\mathcal{I}}$ is the total Fisher information matrix of the system (also called the 'Spatial Fisher memory matrix' in Ref. 6):

$$\bar{\mathcal{I}} = \int_0^\infty e^{\mathbf{J}^\top t}\, \mathbf{C}^{-1}\, e^{\mathbf{J}\, t} \mathrm{d}t \tag{S71}$$

which is symmetric and positive definite by construction. Note that Eq. S70 measures the (upper bound on) the diagonal of what we call the 'cross-temporal decoding matrix' in the main text, i.e. the performance of a time-*dependent* optimal linear decoder, whereas we (optimise and) use time-*independent* decoders for measuring decoding performance in stochastic integrators (roughly corresponding to a horizontal slice of the cross-temporal decoding matrix, Fig. 4a–b). As, in general, the optimal decoder is indeed time-dependent (Eq. S69), a single time-independent linear decoder will not be able to attain optimal performance. Nevertheless, for the integrators we study, $\bar{\mathrm{D}}_{\mathrm{KL}}$ is dominated by persistent activity, which in turn can be decoded by a fixed decoder, and so a time-independent linear decoder is able to achieve near-optimal performance.[1]

We can now define optimal information loading as the $\mathbf{x}(0)$ that maximizes

$$\mathbf{x}^*(0) = \underset{\mathbf{x}(0)}{\mathrm{argmin}}\, \underbrace{\mathbf{x}^\top(0)\, \bar{\mathcal{I}}\, \mathbf{x}(0)}_{\propto \bar{\mathrm{D}}_{\mathrm{KL}}}, \text{ s.t. } \|\mathbf{x}(0)\|_2 = 1 \tag{S72}$$

This is trivially solved by the top eigenvector of $\bar{\mathcal{I}}$, i.e. the eigenvector with the largest eigenvalue. Moreover, because $\bar{\mathcal{I}}$ is symmetric and positive definite (see above), this can be generalized to the first k optimal information loading directions being the top k (orthogonal) eigenvectors of $\bar{\mathcal{I}}$. Importantly, we observe that $\bar{\mathcal{I}}$, as defined in Eq. S71, is the so-called observability Gramian of the system (33), and thus its top k eigenvectors are equivalent to the k most amplifying directions of the system used in our analyses. (Indeed, note the close analogy between Eq. S71 and Eq. S54.) As a technical complication, Eq. S71 defines the observability Gramian with (the square root of) $\mathbf{C}^{-1}$ as the so-called 'readout matrix', whereas for consistency across our analyses (see below), we defined the most amplifying modes as the top eigenvectors of the observability Gramian with simply an identity readout matrix. We confirmed numerically that for the stochastic networks shown in (Fig. 4, Supplementary Fig. 5c,d, and Supplementary Fig. 8a,b,d,e), the top eigenvectors of the observability Gramians defined with these two different readout matrices were highly correlated (average 0.95 for the top $k = 5$ eigenvectors used in our analyses). For all other analyses of most amplifying modes (Fig. 3, Fig. 5f, Fig. 6e, Supplementary Fig. 3e Supplementary Fig. 7, and Supplementary Fig. 10f–h) we used deterministic networks (constructed de-novo, or fitted to stochastic nonlinear networks, or to experimental recordings; Methods S2.4.1 and S2.4.3) and so the choice of an identity readout matrix was justified by a lack of a meaningful definition of $\mathbf{C}$.

S5.8 Optimal information loading: minimising energy with fixed persistent overlap

In our analyses of optimal information loading so far (Eqs. S59, S63 and S72), we have been considering a constraint on the norm of initial conditions in order to make optimization meaningful (as without some constraint, scaling the initial condition will always scale our chosen measures of performance due to the linearity of the dynamics). We now consider a biologically more relevant constraint that is better aligned with the optimization objectives used for most of our numerically optimized neural networks (Fig. 6 and Supplementary Figs. 3 and 11–13) and also standard in previous studies optimising recurrent neural networks(16–19): the energy of the system as defined in Eq. S48. Using the duality of objective functions and constraints in constrained optimization, we seek the initial condition that minimizes energy, ε_t while maintaining a fixed amount of overlap with the persistent mode, p_t (Eq. S56), at the end of a time period of length t. This means we want to solve the following optimization problem:

$$\mathbf{x}^*(0) = \underset{\mathbf{x}(0)}{\mathrm{argmin}}\, \varepsilon_t, \text{ s.t. } p_t = 1 \tag{S73}$$

the solution to which is

$$\mathbf{x}^*(0) \propto \mathbf{V}\, \boldsymbol{\Omega}^{-1}(t)\; \rho(t) \tag{S74}$$

$$\text{where } \rho_i(t) = \Xi_{i1}\, e^{\lambda_i t}, \text{ and } \boldsymbol{\Omega}(t) \text{ is defined in Eq. S52} \tag{S75}$$

In the limit when the delay period is very long, $t \to \infty$, we obtain

$$\mathbf{x}^*(0) \propto \mathbf{V}_{\mathrm{Q}}\, \boldsymbol{\Lambda}_{\mathrm{Q}}^{-1}\, \bar{\rho} \tag{S76}$$

$$\text{where } \bar{\rho} = \mathbf{V}_{\mathrm{Q}}^\top\, \mathbf{U}^\top\, \rho_\infty \mathrel{\overset{\approx}{\propto}} (1, 0, \dots 0)^\top \tag{S77}$$

[1]In other words, although Ref. 6 found fundamentally feedforward dynamics to be optimal for information maintenance, this was only true in general as long as a time-dependent decoder was allowed. The argument above instead suggests a normative justification for attractor, or integrator, dynamics that can render even a time-independent decoder near-optimal.

where $\mathbf{V}_\mathrm{Q}$ and $\mathbf{\Lambda}_\mathrm{Q}$ are respectively the matrix of eigenvectors (as columns) and diagonal matrix of associated eigenvalues of $\mathbf{Q}$, both ordered in decreasing order of the eigenvalues (which are all real, as $\mathbf{Q}$ is positive definite, Sections S5.3 and S5.6), and the approximate proportionality in Eq. S77 is due to $\boldsymbol{\rho}_\infty = \lim_{t\to\infty} \boldsymbol{\rho}(t) \propto (1, 0, \ldots 0)^\top$ (in analogy with Eq. S43), $\mathbf{u}_{(1)}$ being identical to the top eigenvector of $\mathbf{Q}$ (Section S5.6), and the eigenvectors of $\mathbf{Q}$ being orthonormal, because it is symmetric positive definite (Eq. S54). Were this proportionality exact, Eq. S76 could be simply solved as

$$\mathbf{x}^*(0) \overset{\approx}{\propto} \mathbf{u}_{(1)} \tag{S78}$$

In other words, the optimal information loading direction of an integrator network that achieves a fixed level of (asymptotic) persistent overlap while minimising the energy (or, conversely, that maximises the asymptotic persistent overlap given a fixed energy budget) is again approximately the top left eigenvector of the dynamics, i.e. (Section S5.6) the most amplifying mode.

In practice, Eq. S77 only holds approximately, with the ratio of the subsequent elements of $\bar{\boldsymbol{\rho}}$ to the first element being very small, but not quite zero. However, the final weighting of the eigenvectors of $\mathbf{Q}$ in expressing $\mathbf{x}^*(0)$ depends on the product of $\bar{\boldsymbol{\rho}}$ with $\mathbf{\Lambda}_\mathrm{Q}^{-1}$ (Eq. S76), and the (diagonal) elements of $\mathbf{\Lambda}_\mathrm{Q}^{-1}$ (which are the inverses of the diagonal elements of $\mathbf{\Lambda}_\mathrm{Q}$) are ordered exactly oppositely to the elements of $\bar{\boldsymbol{\rho}}$. This partially cancels the preferential weighting of the top eigenvector of $\mathbf{Q}$ by $\bar{\boldsymbol{\rho}}$, and also makes Eq. S78 approximate. Nevertheless, in numerical simulations we found that Eq. S78 held to good precision in random linear integrator networks (such as those in Fig. 3d,e, Fig. 4 and Supplementary Figs. 4 and 5). In Supplementary Fig. 4a,b, we show the relationship between the energy produced by random initial conditions, scaled so that they all result in the same asymptotic persistent overlap, and their overlap with the most persistent versus most amplifying mode. We find that, in general, the overlap with the most amplifying mode (but not with the most persistent mode) is strongly and inversely related to the energy.

While our analytical derivations above concerned the asymptotic limit of an infinitely long delay period, Supplementary Fig. 4c–d show numerical simulations with systematically varied finite delay lengths. In particular, Supplementary Fig. 4c shows that the initial conditions that are optimal for different finite delays (Eq. S74), again in the sense of achieving a fixed persistent overlap by the end of the delay with minimal energy measured over the delay (Eq. S73), show an intuitive dependence on the length of the delay (Supplementary Fig. 4c, bottom). For very short delays, it is best to use inputs that are already aligned with the persistent mode, as there is no time for the dynamics of the system to contribute and gradually align the state with the persistent mode. However, for longer delays, the dynamics can have an increasing contribution, thus increasingly preferring the most amplifying mode over the persistent mode, in qualitative agreement with our analytical results. (We find that even for long delays, the overlap of optimal inputs is not all and none with the most amplifying and persistent modes, respectively, in line with our analysis of the approximate nature of Eq. S78.) In line with these results, the persistent mode achieves optimal to near-optimal performance (i.e. minimal energy) at short delays, but the most amplifying mode performs better (uses less energy) at longer delays (Supplementary Fig. 4d, bottom). (For symmetric networks, Supplementary Fig. 4c–d, top, which we only show for the completeness of analogy with Fig. 3d–e, no such trade-off is observed for the trivial reason that the most persistent and amplifying modes are identical.)

S6 Analysis of canonical nonlinear systems with two stable fixed points

For linear systems that can exhibit persistent activity, we showed that inputs that align with the most amplifying mode generate the most noise-robust (and thus most easily decodable if using multiple inputs) persistent activity (Section S5, see also Figs. 3 and 4 and Supplementary Fig. 5). To develop insights as to how this result might generalise to nonlinear attractor systems, here we study two variants (either symmetric, Supplementary Fig. 6, top, or non-symmetric, Supplementary Fig. 6, bottom) of a canonical minimal nonlinear attractor system, based on the 'unforced Duffing oscillator' (35) (Eqs. S28 and S29; see Supplementary Fig. 6a for flow fields). Specifically, both variants exhibit 3 fixed points: a saddle point at the origin and 2 asymptotically stable fixed points (i.e. attractors) at $(\pm 1, 0)$ (Supplementary Fig. 6a, black crosses). Thus, the (one dimensional) 'coding subspace' of the system along which the two attractors can be best distinguished is the line connecting the two attractors, $\mathbf{c} = (1, 0)^\top$ (Supplementary Fig. 6a, x_1 axes). These two attractors divide the state space of the system into two halves, their respective basins of attraction, which are separated by a manifold, the so-called 'separatrix' (which in this case is one-dimensional; Supplementary Fig. 6a, dashed black lines). This means that the attractor to which the system ultimately converges from a particular initial state simply depends on which side of the separatrix that initial state lies.

S6.1 A local linearization approach based on linearizing around the ground state instead of an attractor

Standard linearization-based approaches to understand the behaviour of nonlinear attractor systems linearize the system's nonlinear dynamics around an *attractor* (21, 36). These approaches are valuable for understanding the *asymptotic* behaviour of the dynamics, once the state of the system is in the sufficiently close vicinity of the attractor, e.g. to understand its stability (21, 35, 36). However, to study information loading, we need to understand the *initial* behaviour of

the system. Thus, our approach is based on a linearization around the fixed point at the *origin* (see also Methods S2.4.2), which serves as the 'ground state'—the state which the system is thought to occupy before the 'stimulus' arrives, and thus with respect to which the magnitude of initial conditions is constrained. Indeed, the origin is an ideal ground state in this case (Supplementary Fig. 6a), not only because it is equidistant from the two attractors, but also because it sits on the separatrix. Thus, as long as the system is in the origin, or—the origin being a saddle point—even if it is perturbed away from the origin along the separatrix, it does not 'choose' between the two attractors.

S6.2 Most persistent and amplifying directions for nonlinear dynamics

Linearizing Eqs. S28 and S29 around the origin yields the Jacobian matrix $\mathbf{J}_s = \begin{pmatrix} 1 & 0 \\ 0 & -1 \end{pmatrix}$ for the symmetric system, and $\mathbf{J}_n = \begin{pmatrix} 1 & 3 \\ 0 & -1 \end{pmatrix}$ for the non-symmetric system. Performing the same analyses on these linearized dynamics as those that we established for genuinely linear dynamics (Fig. 3; see also Methods S2.7.1), reveals that the (locally) 'most persistent' mode of the dynamics, $\mathbf{v}$ (i.e. the direction associated with the top (i.e. largest real) eigenvalue of the Jacobian), in this case represents an unstable direction of the system around the origin (Supplementary Fig. 6a, thin green lines). Although at first it may seem counterintuitive that an unstable direction is called 'persistent', this is actually consistent with our naming convention for linear dynamics not only based on its mathematical definition (see above), but also because—just like for the linear networks we studied—the persistent direction defined in this way corresponds to the coding direction of the system, i.e. $\mathbf{v} = \mathbf{c}$ (in both variants). In addition, these analyses also identify the 'most amplifying' mode, $\mathbf{a}$, (Supplementary Fig. 6a, thin red lines). Intuitively, the (locally) most amplifying mode is orthogonal to the separatrix in both systems (note different scales for the x_1 and x_2 axes in Supplementary Fig. 6a, causing an apparent lack of orthogonality in the bottom panel), i.e. it is the direction that allows the system to start moving away from the separatrix, and thus choose between the two attractors, the fastest. For the symmetric system, this direction is actually identical to the most persistent direction, $\mathbf{a}_s = \mathbf{v}$. However, for the non-symmetric system, the most amplifying mode is at an (approximately 57°) angle to the most persistent mode, $\mathbf{a}_n \approx (0.55, 0.83)^\top$ (unit normalized).

S6.3 Locally most amplifying modes predict optimal inputs, analogous to linear networks

Numerical simulations of the original nonlinear dynamics confirmed that the particular information loading strategies we identified and compared for genuinely linear dynamics (Fig. 3a–c) lead to qualitatively similar behaviours in these nonlinear systems. Specifically, in analogy with our analysis of linear dynamics (Fig. 3b), given a fixed magnitude with respect to the origin (Supplementary Fig. 6a, pale blue ellipses), we compared three different initial conditions that were aligned with either the locally most persistent (Supplementary Fig. 6a, pale green arrows and circles) or most amplifying direction at the origin (Supplementary Fig. 6a, pale red arrows and circles), or with a randomly chosen direction (Supplementary Fig. 6a, gray arrows and circles). We then simulated dynamics starting from each of these initial conditions (Supplementary Fig. 6a, dark green, red, and black arrows) and measured how the overlap of the system's state with the most persistent direction evolved over time (Supplementary Fig. 6b). As expected, there were some unavoidable differences from the behaviour of the linear system: with these nonlinear attractor dynamics, eventually all trajectories converge to one of the attractors, and thus the overlap with the most persistent mode ultimately reaches the same asymptote (at unity) in all cases. As a consequence, the differences in trajectories corresponding to different initial conditions, and their overlap with the most persistent mode, can appear smaller than in the case of linear dynamics (this is especially the case for the non-symmetric system; Supplementary Fig. 6a, bottom). Nevertheless, by measuring the average overlap achieved between $t = 100$ and 250 while we systematically varied the direction of the initial conditions in the full 360° range, we found a monotonic relationship between the initial overlap of the state of the system with the most amplifying mode and this measure of 'late' overlap of the system with the most persistent mode Supplementary Fig. 6c, solid lines). This was qualitatively similar to the behavior of the simple linear networks of Fig. 3, where we also found a monotonic (in that case, linear) relationship between initial amplifying and late persistent overlap (Supplementary Fig. 6c, dashed lines; with late overlap measured between $t = 0.8$ and 2 s). This meant that the most amplifying direction was indeed the optimal direction for 'information loading' in both the nonlinear and linear dynamical systems (Supplementary Fig. 6c, pale red circles and squares). In comparison, with both nonlinear and linear dynamics, random directions or the most persistent direction—when it was different from the most amplifying direction (i.e. in the non-symmetric system; Supplementary Fig. 6a–c, bottom)—achieved lower information loading performance by this measure (Supplementary Fig. 6c, gray and pale green circles and squares).

In summary, we have shown that—just like in the case of linear persistent dynamics—the locally most amplifying direction around the ground state of these nonlinear systems represents the optimal information loading strategy, that is better than 'pushing' the system directly in the direction of the desired attractor (most persistent mode), or in random directions. The intuition is that the locally most amplifying mode is the direction that allows the system to leave the vicinity of the separatrix and travel towards the desired attractor the fastest—this gain in speed quickly offsets the initial disadvantage in closeness to the attractor in comparison to inputs using the most persistent mode. While the size of the effects we obtained were small in some cases, these depended on our choice of parameters and were in part due to the simplicity and

low-dimensionality of these systems. Thus, as the next step, we studied these effects in high-dimensional neural networks with nonlinear dynamics.

S7 Analysis of the nonlinear attractor networks of Fig. 2

To further understand how our theoretical insights gained from analysing linear networks (Section S5, see also Fig. 3 and Supplementary Fig. 5) may generalize to nonlinear attractor networks, we performed the same local (around the origin) linearization of the nonlinear attractor networks of Fig. 2 that we introduced in the previous section (Section S6; see also Methods S2.4.2).

S7.1 The relationship between subspaces extracted from nonlinear dynamics and by local linearization

First, we linearized the dynamics (Eqs. S1 and S2) of both the symmetric and unconstrained networks of Fig. 2 (Methods S2.4.2) and identified the associated most persistent and amplifying modes around the origin based on this local linearization. We then measured the overlap of the (5-dimensional) subspaces spanned by these modes with the subspaces that we extracted from the activities of the original nonlinear networks without linearizing their dynamics (Methods S2.7.1), analysed in Fig. 2f and l. We found that the locally most *persistent* modes overlapped strongly with the persistent subspace of the nonlinear dynamics in which the attractors lie (Supplementary Fig. 7a, green, 'persistent subspace') and overlapped poorly with the persistent nullspace (Supplementary Fig. 7a, green, 'persistent nullspace'). Subspaces (dimensionally matched) spanned by *random* modes showed the opposite pattern: they had small overlaps with the persistent subspace and large overlaps with the persistent nullspace, simply due to the higher dimensionality of the latter (Supplementary Fig. 7a, black, 'persistent subspace' and 'persistent nullspace'). Critically, for both locally most persistent and random modes, these overlaps were essentially identical between symmetric and unconstrained networks (Supplementary Fig. 7a, compare top and bottom panels). In contrast, the overlap of the locally most *amplifying* modes with the persistent subspace and nullspace of the nonlinear dynamics clearly differentiated between symmetric and unconstrained networks. Specifically, in symmetric networks, the most amplifying modes overlapped strongly with the persistent subspace and poorly with the persistent nullspace—as they were identical to the most persistent modes in this case (Supplementary Fig. 7a, top, red, 'persistent subspace' and 'persistent nullspace'). In unconstrained networks, however, they showed a similarly strong overlap with the persistent nullspace as with the persistent subspace (Supplementary Fig. 7a, bottom, red, 'persistent subspace' and 'persistent nullspace').

S7.2 Locally most amplifying modes predict optimal inputs, analogous to linear networks

The results above suggested that our original findings, showing a double dissociation in the efficiency of persistent subspace and nullspace inputs (initial conditions) between symmetric and unconstrained networks (Fig. 2f and l, red and green), are likely explained by how much these inputs align with the locally most amplifying inputs. Indeed, we found that the subspace of numerically optimized inputs (studied in Fig. 2b–e, h–k, and f and l, black) had a substantially larger overlap with the locally most amplifying mode in both classes of networks than with random subspaces, or—in unconstrained networks, in which persistent and amplifying modes are distinguishable—than with the locally most persistent modes (Supplementary Fig. 7a, 'optimal', compare red to black and green). This pattern of overlaps of optimal inputs with the locally most amplifying, persistent and random subspaces was closely analogous to those that we found for the optimized initial conditions in the purely linear networks of Supplementary Fig. 5c,d,g,h (Supplementary Fig. 7a, 'optimal (lin. model)'). We also found a similar pattern of results when performing local linearizations in optimized ring attractor networks (Supplementary Fig. 3e). In sum, these results generalise the results of Section S6 to high dimensional nonlinear neural networks with multiple attractors. They indicate that the locally most amplifying and persistent directions that can be extracted from nonlinear attractor networks around the ground state play functionally similar roles in information loading to the corresponding modes of linear networks of which we have a firm analytical understanding.

S7.3 Overlap with most persistent mode in linear networks predicts decoding accuracy in nonlinear networks

In deterministic linear networks, to allow analytical insights, we used the 'overlap with persistent modes' as a measure of performance (Fig. 3c–e, Supplementary Fig. 5a,b,e,f). In contrast, the measure of performance in stochastic nonlinear networks that we regarded as ultimately relevant, and directly applicable to experimental data, was based on the accuracy of a linear decoder (Fig. 2f,l, Fig. 5b,c, Fig. 6c,d). To study the relationship between these two measures, we simulated both the original stochastic nonlinear networks of Fig. 2 and the deterministic linear dynamics obtained from their local linearization (introduced above), and measured the time evolution of each of these metrics in the corresponding networks when their dynamics were started from initial conditions that were optimized for the decoding accuracy of the nonlinear dynamics (Methods S2.3.1 and S2.7.2) while constrained to be within the locally most persistent, most amplifying, or a

random subspace, as determined by the local linearization, or without any subspace constraints (i.e. as originally done in Fig. 2b–e, h–k, and f and l, black). For a fair comparison, each of the subspaces used for constraining initial conditions were 5-dimensional.

In general, the overlap with the most persistent modes in the locally linearized networks showed somewhat richer dynamics that in the *de novo* linear networks, in which it remained exactly constant or at most decayed exponentially (Fig. 3c,e). Specifically, the overlap measure we used here showed a brief initial decrease for symmetric networks (Supplementary Fig. 7b, top) and mildly non-monotonic time courses for unconstrained networks (Supplementary Fig. 7b, bottom). These were due to measuring overlap here with a 5-dimensional most persistent subspace (appropriate for networks distinguishing between 6 cue conditions), including some directions that were associated with eigenvalues that could have slightly negative real parts and non-zero complex parts (besides the one that was defined to have a zero eigenvalue, see Methods S2.4.2). In contrast, in Fig. 3, we measured overlap with the single most persistent direction of the dynamics (distinguishing between only two cue conditions) that was constructed to be exactly persistent, i.e. with a zero eigenvalue.

Despite these differences, we found that the overlap measure for the locally linearized networks showed qualitatively similar patterns as in the *de novo* large linear networks of Fig. 3e. For symmetric networks (Supplementary Fig. 7b, top), the overlap was largely constant in time for each initial condition, with most persistent and amplifying inputs achieving identical performances Supplementary Fig. 7b, top, red and green), substantially above that of random inputs (Supplementary Fig. 7b, top, black), and on par—and by this measure, even somewhat better—than that of optimal inputs (Supplementary Fig. 7b, top, blue). For unconstrained networks (Supplementary Fig. 7b, bottom), the overlap for most persistent inputs again remained roughly constant in time (Supplementary Fig. 7b, bottom, green). For most amplifying inputs, overlap started from a lower level initially, but it eventually overtook the level achieved by most persistent inputs (Supplementary Fig. 7b, bottom, red). Random inputs performed worst (Supplementary Fig. 7b, bottom, black), and optimal inputs showed a similar trajectory to that of most amplifying inputs (initially low overlap which then increased to above that achieved by most persistent inputs), though at a somewhat lower performance by this measure (Supplementary Fig. 7b, bottom, blue vs. red).

Not only did these overlap measures of linearized networks retain some important qualitative characteristics of the overlap measures used in *de novo* linear networks, they also showed a clear relationship with our ultimate measure of interest: decoding accuracy in the corresponding stochastic nonlinear networks (using a 'noise-matched' scenario—see Fig. 4a—in which all networks had the same level of noise in their dynamics; Methods S2.4.1). In symmetric networks, most persistent and amplifying inputs achieved maximal performance (as did the optimal inputs), with random inputs performing substantially more poorly (Supplementary Fig. 7c, top). In unconstrained networks, most amplifying (and optimal) inputs outperformed most persistent inputs, with random inputs performing worst again (Supplementary Fig. 7c, bottom). Thus, the overall rank ordering of different inputs for information loading, as measured by eventual decoding accuracy, is well predicted by their rank ordering on the overlap measure.

S7.4 Fitting linear dynamics reveals information loading strategies in nonlinear networks

We have shown so far that the same principles (and measures) determining the efficiency of different information loading strategies that we analytically identified in linear networks also apply to nonlinear networks. However, our approach for this was based on a local linearization of the original nonlinear dynamics, which required knowledge of the true equations governing the dynamics of these networks. This is obviously not available for experimental data. Thus, in order to be able to apply our theoretically derived measures of optimal information loading without having access to the true dynamics of the system, we analyzed experimental data by fitting neural responses by linear neural networks (Fig. 5d–f; see also Methods S2.4.3). As a consequence, we wanted to validate that this fitting-based approach provides meaningful results, and has the capacity to reveal information loading strategies in nonlinear networks even when

1. we do not have access to the true dynamics but only to samples of activities generated by those dynamics; and
2. we also cannot assume that the true dynamics are linear.

For this, we repeated the same analyses with our simulated nonlinear networks while using 'ground truth' information loading strategies, i.e. inputs confined to the most persistent, or most amplifying, or random, or optimal subspaces as defined above (Section S7.3).

As in the previous section (Section S7.3), we used a 'noise-matched' scenario—see Fig. 4a—in which all networks had the same level of noise in their dynamics; Methods S2.4.1). A more realistic comparison might require 'performance matched' simulations, as in Fig. 3c, in which different networks are allowed to have different amounts of noise in their dynamics such that it is instead the asymptotic level of (decoding) performance that is matched between them. However, we found that our main metric of subspace overlap was robust to changes in noise levels, so we expect that all our results hold to high precision in the performance matched regime as well.

For symmetric networks, we found that linear dynamical systems fitted to responses that resulted from using optimal inputs yielded similar dynamics to those following either most persistent or most amplifying inputs (Supplementary Fig. 7d; top, compare ‘persistent’, ‘amplifying’, and ‘optimal’). Initial overlap was well above chance with both the most amplifying and persistent modes (though higher for the former), such that the overlap with most amplifying modes remained constant, while the overlap with most persistent modes increased over time. In contrast, the initial overlap resulting from random inputs with either persistent or amplifying modes was close to chance (with overlap with amplifying once again remaining constant, while overlap with persistent increasing over time). Note that all of these were unlike the patterns we saw in experimental data (Fig. 5f).

For unconstrained networks, most amplifying and optimal inputs again resulted in similar dynamics (Supplementary Fig. 7d; bottom, compare ‘amplifying’ and ‘optimal’). Initial overlap was high with most amplifying modes but low (at chance) with persistent modes before the dynamics ultimately overlapped strongly with the persistent modes and weakly with the amplifying modes. This was in contrast to dynamics following persistent or random inputs, in which there was no clear difference in initial overlaps with most amplifying vs. most persistent modes, neither was there a clear decrease in overlap with the most amplifying modes over time (Supplementary Fig. 7d, compare ‘persistent’ and ‘random’). Note that experimental data was only consistent with the former but not the latter pattern of results (Fig. 5f).

S8 Fisher information in task-optimized ring attractor networks

Here, we note that the cosine term in the cost function (Eq. S4) for the ring attractor networks that we optimize in Methods S2.3.4, quantifying the precision of the decoded angle, is closely related to a cost measuring the population Fisher information about angle. Intuitively, both cost functions quantify the ability of the network to perform fine discrimination of stimulus angles. To see this more formally, recall that the Fisher information in this case is

$$\mathcal{I}_{\theta^{(c)}} = -\int \mathcal{P}\left(\mathbf{r}|\theta^{(c)}\right) \left.\frac{\partial^2}{\partial\theta^2}\right|_{\theta\,=\,\theta^{(c)}} \ln \mathcal{P}\left(\mathbf{r}|\theta\right) \mathrm{d}\mathbf{r} \tag{S79}$$

where, as in Methods S2.3.4, $\theta^{(c)}$ is the true stimulus angle on a trial (the target for the decoder), $\mathbf{r}$ is the population response, and $\mathcal{P}\left(\mathbf{r}|\theta\right)$ is the distribution of responses the network generates to some stimulus angle θ. In the limit of a sufficiently large population, the maximum likelihood estimator, $\hat{\theta}_{\mathrm{ML}}$ achieves the same Fisher information as the full population vector, and is distributed as a (circular) Gaussian (von Mises) distribution centered on the true orientation, $\theta^{(c)}$, with some constant (circular) concentration, κ, so we can write

$$\mathcal{I}_{\theta^{(c)}} \simeq -\int \mathcal{P}\left(\hat{\theta}_{\mathrm{ML}}|\theta^{(c)}\right) \left.\frac{\partial^2}{\partial\theta^2}\right|_{\theta\,=\,\theta^{(c)}} \ln \mathcal{P}\left(\hat{\theta}_{\mathrm{ML}}|\theta\right) \mathrm{d}\hat{\theta}_{\mathrm{ML}} \tag{S80}$$

$$= \kappa \int \mathcal{P}\left(\hat{\theta}_{\mathrm{ML}}|\theta^{(c)}\right) \cos\left(\hat{\theta}_{\mathrm{ML}} - \theta^{(c)}\right) \mathrm{d}\hat{\theta}_{\mathrm{ML}} \tag{S81}$$

Assuming that the population vector-based decoder we use for training networks, $\hat{\theta}$ (Eq. S5 in Methods S2.3.4), is an efficient estimator that approximates the maximum likelihood decoder, $\hat{\theta} \simeq \hat{\theta}_{\mathrm{ML}}$[2], and substituting the integral over the distribution of the estimate with an empirical average over its stochastic realizations, we can further rewrite the Fisher information as

$$\mathcal{I}_{\theta^{(c)}} \simeq \kappa \left\langle \cos\left(\hat{\theta}_{\mathrm{ML}} - \theta^{(c)}\right) \right\rangle \tag{S82}$$

Eq. S82 is thus identical to the integrand of the first term in Eq. S4 up to a multiplicative constant (which can be incorporated into $\alpha^{(1)}_{\mathrm{nonlin}}$ in Eq. S4), an additive constant (the 1 inside the square bracket in Eq. S4), which does not matter for optimization, and a sign-flip, because we are maximizing Fisher information in Eq. S82 but minimizing the cost in Eq. S4. Therefore, minimizing (the first term in) Eq. S4 also (approximately) maximizes Eq. S79 (averaged over time points at which the network is decoded and over true stimulus angles), and vice versa.

[2] We also checked empirically that, in line with these assumptions, the empirical distribution of $\hat{\theta}$ was well approximated by a von Mises distribution centered on $\theta^{(c)}$ with a constant concentration across target angles.

References

[1] Wimmer K, Nykamp DQ, Constantinidis C, Compte A (2014) Bump attractor dynamics in prefrontal cortex explains behavioral precision in spatial working memory. *Nature Neuroscience* 17(3):431–439.

[2] Brunel N (2003) Dynamics and Plasticity of Stimulus-selective Persistent Activity in Cortical Network Models. *Cerebral Cortex* 13:1151–1161.

[3] Brody CD, Romo R, Kepecs A (2003) Basic mechanisms for graded persistent activity: Discrete attractors, continuous attractors, and dynamic representations. *Current Opinion in Neurobiology* 13(2):204–211.

[4] Murray JD, et al. (2017) Stable population coding for working memory coexists with heterogeneous neural dynamics in prefrontal cortex. *Proceedings of the National Academy of Sciences* 114(2):394–399.

[5] Goldman MS (2009) Memory without Feedback in a Neural Network. *Neuron* 61(4):621–634.

[6] Ganguli S, Huh D, Sompolinsky H (2008) Memory traces in dynamical systems. *Proceedings of the National Academy of Sciences of the United States of America* 105(48):18970–18975.

[7] Elsayed GF, Lara AH, Kaufman MT, Churchland MM, Cunningham JP (2016) Reorganization between preparatory and movement population responses in motor cortex. *Nature Communications* 7(1):13239.

[8] Wasmuht DF (2019) Ph.D. thesis (University of Oxford).

[9] Watanabe K, Funahashi S (2014) Neural mechanisms of dual-task interference and cognitive capacity limitation in the prefrontal cortex. *Nature Neuroscience* 17(4):601–611.

[10] Watanabe K, Funahashi S (2015) A dual-task paradigm for behavioral and neurobiological studies in nonhuman primates. *Journal of Neuroscience Methods* 246:1–12.

[11] Spaak E, Watanabe K, Funahashi S, Stokes MG (2017) Stable and Dynamic Coding for Working Memory in Primate Prefrontal Cortex. *The Journal of Neuroscience* 37(27):6503–6516.

[12] Watanabe K, Kadohisa M, Kusunoki M, Buckley M, Duncan J (2023) Cycles of goal silencing and reactivation underlie complex problem-solving in primate frontal and parietal cortex. *Nature Communications* p. (In Press).

[13] Drucker CB, Carlson ML, Toda K, DeWind NK, Platt ML (2015) Non-invasive primate head restraint using thermoplastic masks. *Journal of Neuroscience Methods* 253:90–100.

[14] Ninomiya T, Dougherty K, Godlove DC, Schall JD, Maier A (2015) Microcircuitry of agranular frontal cortex: contrasting laminar connectivity between occipital and frontal areas. *Journal of Neurophysiology* 113(9):3242–3255.

[15] Hennequin G, Ahmadian Y, Rubin DB, Lengyel M, Miller KD (2018) The Dynamical Regime of Sensory Cortex: Stable Dynamics around a Single Stimulus-Tuned Attractor Account for Patterns of Noise Variability. *Neuron* 98(4):846–860.e5.

[16] Song HF, Yang GR, Wang XJ (2016) Training Excitatory-Inhibitory Recurrent Neural Networks for Cognitive Tasks: A Simple and Flexible Framework. *PLoS Computational Biology* 12(2):1–30.

[17] Orhan AE, Ma WJ (2019) A diverse range of factors affect the nature of neural representations underlying short-term memory. *Nature Neuroscience* 22(2):275–283.

[18] Yang GR, Joglekar MR, Song HF, Newsome WT, Wang XJ (2019) Task representations in neural networks trained to perform many cognitive tasks. *Nat. Neurosci.* 22(2):297–306.

[19] Sussillo D, Churchland MM, Kaufman MT, Shenoy KV (2015) A neural network that finds a naturalistic solution for the production of muscle activity. *Nature Neuroscience* 18(7):1025–1033.

[20] Kingma DP, Ba JL (2014) Adam: A method for stochastic optimization. *arXiv* p. 1412.6980.

[21] Mante V, Sussillo D, Shenoy KV, Newsome WT (2013) Context-dependent computation by recurrent dynamics in prefrontal cortex. *Nature* 503(7474):78–84.

[22] Cueva CJ, et al. (2020) Low-dimensional dynamics for working memory and time encoding. *Proceedings of the National Academy of Sciences of the United States of America* 117(37):23021–23032.

[23] Barak O, Sussillo D, Romo R, Tsodyks M, Abbott LF (2013) From fixed points to chaos: Three models of delayed discrimination. *Progress in Neurobiology* 103:214–222.

[24] Seung HS (1996) How the brain keeps the eyes still. *Proceedings of the National Academy of Sciences of the United States of America* 93(23):13339–13344.

[25] Druckmann S, Chklovskii DB (2012) Neuronal circuits underlying persistent representations despite time varying activity. *Current Biology* 22(22):2095–2103.

[26] Cannon SC, Robinson DA, Shamma S (1983) A proposed neural network for the integrator of the oculomotor system. *Biological Cybernetics* 49(2):127–136.

[27] Dayan P, Abbott L (2001) *Theoretical Neuroscience: Computational and Mathematical Modeling of Neural Systems.* (MIT Press).

[28] Churchland MM, et al. (2012) Neural population dynamics during reaching. *Nature* 487(7405):51–6.

[29] Hennequin G, Vogels TP, Gerstner W (2014) Optimal control of transient dynamics in balanced networks supports generation of complex movements. *Neuron* 82(6):1394–1406.

[30] Kao TC, Sadabadi MS, Hennequin G (2021) Optimal anticipatory control as a theory of motor preparation: A thalamocortical circuit model. *Neuron* 109(9):1567–1581.

[31] Chadwick A, et al. (2023) Learning shapes cortical dynamics to enhance integration of relevant sensory input. *Neuron* 111(1):106–120.

[32] Hopfield JJ (1982) Neural networks and physical systems with emergent collective computational abilities. *Proceedings of the National Academy of Sciences of the United States of America* 79(8):2554–2558.

[33] Kao TC, Hennequin G (2019) Neuroscience out of control: control-theoretic perspectives on neural circuit dynamics. *Current Opinion in Neurobiology* 58:122–129.

[34] Stroud JP, Porter MA, Hennequin G, Vogels TP (2018) Motor primitives in space and time via targeted gain modulation in cortical networks. *Nature Neuroscience* 21(12):1774–1783.

[35] Wiggins S (2003) *Introduction to Applied Nonlinear Dynamical Systems and Chaos.* (Springer), p. 255.

[36] Sussillo D, Barak O (2013) Opening the black box: Low-dimensional dynamics in high-dimensional recurrent neural networks. *Neural Computation* 25(3):626–649.
